# Supplementary material for: Protein language model-based prediction for plant miRNA encoded peptides
Source: PeerJ Comput Sci. 2025 Mar 18;11:e2733. doi: 10.7717/peerj-cs.2733 (PMC11935769; doi:10.7717/peerj-cs.2733)
Supplement: Supplemental Information 2 [file peerj-cs-11-2733-s002.docx]

**Table S1.** The ranges of hyperparameters for different machine learning models used in this study.

| **Learning algorithms** | **Hyperparameters and their ranges** |
| --- | --- |
| LR | C (15, 10, 5, 1, 0.1, 0.01, 0.001, 0.0001) |
| MLP (ANN) | alpha (10, 9, 8, 7, 6, 5, 4, 3, 2, 1, 1e-1, 1e-2, 1e-3, 1e-4, 1e-5, 1e-6) |
| SVM | C (1500, 1000, 500, 250, 100, 50, 25, 10, 1, 0.5);  gamma (0.005) |
| AdaBoost | learning_rate (0.05, 0.1, 0.5);  n_estimators (Increases from 1000 to 2200 in steps of 100) |
| XGBoost | learning_rate (0.01, 0.05, 0.1);  n_estimators (Increases from 3300 to 3800 in steps of 100); max_depth (Increases from 18 to 22 in steps of 1) |
| ERT | n_estimators (Increases from 2000 to 3500 in steps of 100); max_features (Increases from 25 to 36 in steps of 1) |
| RF | n_estimators (Increases from 2000 to 3100 in steps of 100); max_depth (Increases from 20 to 31 in steps of 1) |
| KNN | n_neighbors (Increases from 5 to 15 in steps of 1) |
