# Supplementary material for: Protein language model-based prediction for plant miRNA encoded peptides
Source: PeerJ Comput Sci. 2025 Mar 18;11:e2733. doi: 10.7717/peerj-cs.2733 (PMC11935769; doi:10.7717/peerj-cs.2733)
Supplement: Supplemental Information 3 — NOTE: - indicates that the evaluation of this metric was not conducted in the corresponding work. [file peerj-cs-11-2733-s003.docx]

**Table S2.** Performance comparison with SOTA models on bioactive peptides datasets.

| **Dataset** | **Model** | **Sp** | **ACC** | **Pre** | **Recall** | **F1** | **AUPR** |
| --- | --- | --- | --- | --- | --- | --- | --- |
| Neuropeptides dataset | PredNeuroP | 0.907 | **0.897** | - | **0.886** | - | - |
|  | UniDL4BioPep | 0.909 | 0.892 | - | 0.875 | - | - |
|  | pLM4PEP | **0.998** | 0.732 | 0.996 | 0.466 | 0.635 | 0.978 |
| Blood-brain barrier peptides dataset | BBPpred | 0.947 | 0.790 | 0.923 | 0.632 | **0.750** | 0.932 |
|  | UniDL4BioPep | 0.809 | **0.842** | - | **0.882** | - | - |
|  | pLM4PEP | **1.000** | 0.658 | **1.000** | 0.316 | 0.480 | **0.935** |
| Anti-parasitic peptides dataset | PredAPP | 0.783 | 0.880 | - | **0.978** | 0.891 | 0.903 |
|  | UniDL4BioPep | **1.000** | 0.891 | - | 0.821 | - | - |
|  | pLM4PEP | **1.000** | **0.902** | 1.000 | 0.804 | **0.892** | **0.988** |

**NOTE:** - indicates that the evaluation of this metric was not conducted in the corresponding work.
