# Supplementary material for: Protein language model-based prediction for plant miRNA encoded peptides
Source: PeerJ Comput Sci. 2025 Mar 18;11:e2733. doi: 10.7717/peerj-cs.2733 (PMC11935769; doi:10.7717/peerj-cs.2733)
Supplement: Supplemental Information 4 [file peerj-cs-11-2733-s004.docx]

**Table S3.** Performance comparison with SOTA model on plant peptide datasets.

| **AUROC** | **Antimicrobial** | **Antibacterial** | **Antifungal** | **Antiviral** |
| --- | --- | --- | --- | --- |
| pLM4PEP | 0.956 | 0.872 | 0.831 | 0.767 |
| PTPAMP | 0.930 | 0.900 | 0.900 | 0.820 |
