# Supplementary material for: Protein language model-based prediction for plant miRNA encoded peptides
Source: PeerJ Comput Sci. 2025 Mar 18;11:e2733. doi: 10.7717/peerj-cs.2733 (PMC11935769; doi:10.7717/peerj-cs.2733)
Supplement: Supplemental Information 5 [file peerj-cs-11-2733-s005.doc]

Translations for the non-English text in the original raw data file:

**1. main.py**

（1）切换模型：switch models

（2）参数寻优：parameter optimization

（3）选取LR作为分类器：select LR as the classifier

**2. ML_grid_search_model.py**

（1）gamma为核函数系数：gamma is the kernel function coefficient

**3. predictor.py**

（1）拟合数据：fit the data
