# Supplementary material for: Protein language model-based prediction for plant miRNA encoded peptides
Source: PeerJ Comput Sci. 2025 Mar 18;11:e2733. doi: 10.7717/peerj-cs.2733 (PMC11935769; doi:10.7717/peerj-cs.2733)
Supplement: Supplemental Information 1 [file peerj-cs-11-2733-s001.zip › pLM4PEP-master/pydpi/drug/basak.html]

Python: module basak

|  |  |
| --- | --- |
| **basak** | index /home/orient/pydpi/src/pydpi/drug/basak.py |

##############################################################################  
The calculation of some commonly used basak information index  based on its   
   
topological structure. You can get 21 molecular connectivity descriptors.   
   
You can freely use and distribute it. If you hava  any problem, you could   
   
contact with us timely!  
   
Authors: Dongsheng Cao and Yizeng Liang.  
   
Date: 2012.11.15  
   
Email: oriental-cds@163.com  
##############################################################################

**Modules**

|  |  |  |  |
| --- | --- | --- | --- |
| rdkit.Chem | copy | numpy |  |

|  |  |  |
| --- | --- | --- |
| **Functions** | | |
|  |  | **CalculateBasakCIC0**(mol)  #################################################################  Obtain the complementary information content with order 0      proposed by Basak     ---->CIC0  #################################################################  **CalculateBasakCIC1**(mol)  #################################################################  Obtain the complementary information content with order 1 proposed      by Basak.     ---->CIC1  #################################################################  **CalculateBasakCIC2**(mol)  #################################################################  Obtain the complementary information content with order 2 proposed      by Basak.     ---->CIC2  #################################################################  **CalculateBasakCIC3**(mol)  #################################################################  Obtain the complementary information content with order 3 proposed      by Basak.     ---->CIC3  #################################################################  **CalculateBasakCIC4**(mol)  #################################################################  Obtain the complementary information content with order 4 proposed      by Basak.     ---->CIC4  #################################################################  **CalculateBasakCIC5**(mol)  #################################################################  Obtain the complementary information content with order 5 proposed      by Basak.     ---->CIC5  #################################################################  **CalculateBasakCIC6**(mol)  #################################################################  Obtain the complementary information content with order 6 proposed      by Basak.     ---->CIC6  #################################################################  **CalculateBasakIC0**(mol)  #################################################################  Obtain the information content with order 0 proposed by Basak     ---->IC0  #################################################################  **CalculateBasakIC1**(mol)  #################################################################  Obtain the information content with order 1 proposed by Basak     ---->IC1  #################################################################  **CalculateBasakIC2**(mol)  #################################################################  Obtain the information content with order 2 proposed by Basak     ---->IC2  #################################################################  **CalculateBasakIC3**(mol)  #################################################################  Obtain the information content with order 3 proposed by Basak     ---->IC3  #################################################################  **CalculateBasakIC4**(mol)  #################################################################  Obtain the information content with order 4 proposed by Basak     ---->IC4  #################################################################  **CalculateBasakIC5**(mol)  #################################################################  Obtain the information content with order 5 proposed by Basak     ---->IC5  #################################################################  **CalculateBasakIC6**(mol)  #################################################################  Obtain the information content with order 6 proposed by Basak     ---->IC6  #################################################################  **CalculateBasakSIC0**(mol)  #################################################################  Obtain the structural information content with order 0      proposed by Basak     ---->SIC0  #################################################################  **CalculateBasakSIC1**(mol)  #################################################################  Obtain the structural information content with order 1      proposed by Basak.     ---->SIC1  #################################################################  **CalculateBasakSIC2**(mol)  #################################################################  Obtain the structural information content with order 2 proposed      by Basak.     ---->SIC2  #################################################################  **CalculateBasakSIC3**(mol)  #################################################################  Obtain the structural information content with order 3 proposed      by Basak.     ---->SIC3  #################################################################  **CalculateBasakSIC4**(mol)  #################################################################  Obtain the structural information content with order 4 proposed      by Basak.     ---->SIC4  #################################################################  **CalculateBasakSIC5**(mol)  #################################################################  Obtain the structural information content with order 5 proposed      by Basak.     ---->SIC5  #################################################################  **CalculateBasakSIC6**(mol)  #################################################################  Obtain the structural information content with order 6 proposed      by Basak.     ---->SIC6  #################################################################  **Getbasak**(mol)  #################################################################  Get the dictionary of basak descriptors for given moelcule mol  ################################################################# |

|  |  |  |
| --- | --- | --- |
| **Data** | | |
|  |  | **Version** = 1.0 |
