# Supplementary material for: Protein language model-based prediction for plant miRNA encoded peptides
Source: PeerJ Comput Sci. 2025 Mar 18;11:e2733. doi: 10.7717/peerj-cs.2733 (PMC11935769; doi:10.7717/peerj-cs.2733)
Supplement: Supplemental Information 1 [file peerj-cs-11-2733-s001.zip › pLM4PEP-master/pydpi/html/AAComposition.html]

Python: module AAComposition

|  |  |
| --- | --- |
| **AAComposition** | index /home/orient/ProPy/AAComposition.py |

###############################################################################  
   
The module is used for computing the composition of amino acids, dipetide and   
   
3-mers (tri-peptide) for a given protein sequence. You can get 8420 descriptors   
   
for a given protein sequence. You can freely use and distribute it. If you hava   
   
any problem, you could contact with us timely!  
   
References:  
   
[1]: Reczko, M. and Bohr, H. (1994) The DEF data base of sequence based protein  
   
fold class predictions. Nucleic Acids Res, 22, 3616-3619.  
   
[2]: Hua, S. and Sun, Z. (2001) Support vector machine approach for protein  
   
subcellular localization prediction. Bioinformatics, 17, 721-728.  
   
   
[3]:Grassmann, J., Reczko, M., Suhai, S. and Edler, L. (1999) Protein fold class  
   
prediction: new methods of statistical classification. Proc Int Conf Intell Syst Mol  
   
Biol, 106-112.  
   
Authors: Dongsheng Cao and Yizeng Liang.  
   
Date: 2012.3.27  
   
Email: oriental-cds@163.com  
   
###############################################################################

**Modules**

|  |  |  |  |
| --- | --- | --- | --- |
| re |  |  |  |

|  |  |  |
| --- | --- | --- |
| **Functions** | | |
|  |  | **CalculateAAComposition**(ProteinSequence)  ########################################################################  Calculate the composition of Amino acids      for a given protein sequence.     Usage:     result=CalculateAAComposition(protein)     Input: protein is a pure protein sequence.     Output: result is a dict form containing the composition of      20 amino acids.  ########################################################################  **CalculateAADipeptideComposition**(ProteinSequence)  ########################################################################  Calculate the composition of AADs, dipeptide and 3-mers for a      given protein sequence.     Usage:     result=CalculateAADipeptideComposition(protein)     Input: protein is a pure protein sequence.     Output: result is a dict form containing all composition values of      AADs, dipeptide and 3-mers (8420).  ########################################################################  **CalculateDipeptideComposition**(ProteinSequence)  ########################################################################  Calculate the composition of dipeptidefor a given protein sequence.     Usage:      result=CalculateDipeptideComposition(protein)     Input: protein is a pure protein sequence.     Output: result is a dict form containing the composition of      400 dipeptides.  ########################################################################  **GetSpectrumDict**(proteinsequence)  ########################################################################  Calcualte the spectrum descriptors of 3-mers for a given protein.     Usage:      result=GetSpectrumDict(protein)     Input: protein is a pure protein sequence.     Output: result is a dict form containing the composition values of 8000     3-mers.  ########################################################################  **Getkmers**()  ########################################################################  Get the amino acid list of 3-mers.      Usage:      result=Getkmers()     Output: result is a list form containing 8000 tri-peptides.     ######################################################################## |

|  |  |  |
| --- | --- | --- |
| **Data** | | |
|  |  | **AALetter** = ['A', 'R', 'N', 'D', 'C', 'E', 'Q', 'G', 'H', 'I', 'L', 'K', 'M', 'F', 'P', 'S', 'T', 'W', 'Y', 'V'] |
