# Supplementary material for: Protein language model-based prediction for plant miRNA encoded peptides
Source: PeerJ Comput Sci. 2025 Mar 18;11:e2733. doi: 10.7717/peerj-cs.2733 (PMC11935769; doi:10.7717/peerj-cs.2733)
Supplement: Supplemental Information 1 [file peerj-cs-11-2733-s001.zip › pLM4PEP-master/pydpi/html/Autocorrelation.html]

Python: module Autocorrelation

|  |  |
| --- | --- |
| **Autocorrelation** | index /home/orient/ProPy/Autocorrelation.py |

##########################################################################################  
   
This module is used for computing the Autocorrelation descriptors based different  
   
 properties of AADs.You can also input your properties of AADs, then it can help you  
   
to compute Autocorrelation descriptors based on the property of AADs. Currently, You   
   
can get 720 descriptors for a given protein sequence based on our provided physicochemical  
   
properties of AADs. You can freely use and distribute it. If you hava  any problem,   
   
you could contact with us timely!  
   
References:  
   
[1]: http://www.genome.ad.jp/dbget/aaindex.html  
   
[2]:Feng, Z.P. and Zhang, C.T. (2000) Prediction of membrane protein types based on  
   
the hydrophobic index of amino acids. J Protein Chem, 19, 269-275.  
   
[3]:Horne, D.S. (1988) Prediction of protein helix content from an autocorrelation  
   
analysis of sequence hydrophobicities. Biopolymers, 27, 451-477.  
   
[4]:Sokal, R.R. and Thomson, B.A. (2006) Population structure inferred by local  
   
spatial autocorrelation: an Usage from an Amerindian tribal population. Am J  
   
Phys Anthropol, 129, 121-131.  
   
Authors: Dongsheng Cao and Yizeng Liang.  
   
Date: 2010.11.22  
   
Email: oriental-cds@163.com  
   
##########################################################################################

**Modules**

|  |  |  |  |
| --- | --- | --- | --- |
| math | string |  |  |

|  |  |  |
| --- | --- | --- |
| **Functions** | | |
|  |  | **CalculateAutoTotal**(ProteinSequence)  ####################################################################################  A method used for computing all autocorrelation descriptors based on 8 properties of AADs.     Usage:     result=CalculateGearyAutoTotal(protein)     Input: protein is a pure protein sequence.     Output: result is a dict form containing 30\*8\*3=720 normalized Moreau Broto, Moran, and Geary     autocorrelation descriptors based on the given properties(i.e., \_AAPropert).  ####################################################################################  **CalculateEachGearyAuto**(ProteinSequence, AAP, AAPName)  ####################################################################################  you can use the function to compute GearyAuto     descriptors for different properties based on AADs.     Usage:     result=CalculateEachGearyAuto(protein,AAP,AAPName)     Input: protein is a pure protein sequence.     AAP is a dict form containing the properties of 20 amino acids (e.g., \_AvFlexibility).     AAPName is a string used for indicating the property (e.g., '\_AvFlexibility').      Output: result is a dict form containing 30 Geary autocorrelation     descriptors based on the given property.  ####################################################################################  **CalculateEachMoranAuto**(ProteinSequence, AAP, AAPName)  ####################################################################################  you can use the function to compute MoranAuto     descriptors for different properties based on AADs.     Usage:     result=CalculateEachMoranAuto(protein,AAP,AAPName)     Input: protein is a pure protein sequence.     AAP is a dict form containing the properties of 20 amino acids (e.g., \_AvFlexibility).     AAPName is a string used for indicating the property (e.g., '\_AvFlexibility').      Output: result is a dict form containing 30 Moran autocorrelation     descriptors based on the given property.  ####################################################################################  **CalculateEachNormalizedMoreauBrotoAuto**(ProteinSequence, AAP, AAPName)  ####################################################################################  you can use the function to compute MoreauBrotoAuto     descriptors for different properties based on AADs.     Usage:     result=CalculateEachNormalizedMoreauBrotoAuto(protein,AAP,AAPName)     Input: protein is a pure protein sequence.     AAP is a dict form containing the properties of 20 amino acids (e.g., \_AvFlexibility).     AAPName is a string used for indicating the property (e.g., '\_AvFlexibility').      Output: result is a dict form containing 30 Normalized Moreau-Broto autocorrelation     descriptors based on the given property.  ####################################################################################  **CalculateGearyAuto**(ProteinSequence, AAProperty, AAPropertyName)  ####################################################################################  A method used for computing GearyAuto for all properties     Usage:     result=CalculateGearyAuto(protein,AAP,AAPName)     Input: protein is a pure protein sequence.     AAProperty is a list or tuple form containing the properties of 20 amino acids (e.g., \_AAProperty).     AAPName is a list or tuple form used for indicating the property (e.g., '\_AAPropertyName').      Output: result is a dict form containing 30\*p Geary autocorrelation     descriptors based on the given properties.  ####################################################################################  **CalculateGearyAutoAvFlexibility**(ProteinSequence)  ####################################################################################  Calculte the GearyAuto Autocorrelation descriptors based on     AvFlexibility.     Usage:  result=CalculateGearyAutoAvFlexibility(protein)     Input: protein is a pure protein sequence.     Output: result is a dict form containing 30 Geary Autocorrelation     descriptors based on AvFlexibility.  ####################################################################################  **CalculateGearyAutoFreeEnergy**(ProteinSequence)  ####################################################################################  Calculte the GearyAuto Autocorrelation descriptors based on     FreeEnergy.     Usage:     result=CalculateGearyAutoFreeEnergy(protein)     Input: protein is a pure protein sequence.     Output: result is a dict form containing 30 Geary Autocorrelation     descriptors based on FreeEnergy.  ####################################################################################  **CalculateGearyAutoHydrophobicity**(ProteinSequence)  ####################################################################################  Calculte the GearyAuto Autocorrelation descriptors based on     hydrophobicity.     Usage:     result=CalculateGearyAutoHydrophobicity(protein)     Input: protein is a pure protein sequence.     Output: result is a dict form containing 30 Geary Autocorrelation     descriptors based on hydrophobicity.  ####################################################################################  **CalculateGearyAutoMutability**(ProteinSequence)  ####################################################################################  Calculte the GearyAuto Autocorrelation descriptors based on     Mutability.     Usage:     result=CalculateGearyAutoMutability(protein)     Input: protein is a pure protein sequence.     Output: result is a dict form containing 30 Geary Autocorrelation     descriptors based on Mutability.  ####################################################################################  **CalculateGearyAutoPolarizability**(ProteinSequence)  ####################################################################################  Calculte the GearyAuto Autocorrelation descriptors based on     Polarizability.     Usage:     result=CalculateGearyAutoPolarizability(protein)     Input: protein is a pure protein sequence.     Output: result is a dict form containing 30 Geary Autocorrelation     descriptors based on Polarizability.  ####################################################################################  **CalculateGearyAutoResidueASA**(ProteinSequence)  ####################################################################################  Calculte the GearyAuto Autocorrelation descriptors based on     ResidueASA.     Usage:     result=CalculateGearyAutoResidueASA(protein)     Input: protein is a pure protein sequence.     Output: result is a dict form containing 30 Geary Autocorrelation     descriptors based on ResidueASA.  ####################################################################################  **CalculateGearyAutoResidueVol**(ProteinSequence)  ####################################################################################  Calculte the GearyAuto Autocorrelation descriptors based on     ResidueVol.     Usage:     result=CalculateGearyAutoResidueVol(protein)     Input: protein is a pure protein sequence.     Output: result is a dict form containing 30 Geary Autocorrelation     descriptors based on ResidueVol.  ####################################################################################  **CalculateGearyAutoSteric**(ProteinSequence)  ####################################################################################  Calculte the GearyAuto Autocorrelation descriptors based on     Steric.     Usage:     result=CalculateGearyAutoSteric(protein)     Input: protein is a pure protein sequence.     Output: result is a dict form containing 30 Geary Autocorrelation     descriptors based on Steric.  ####################################################################################  **CalculateGearyAutoTotal**(ProteinSequence)  ####################################################################################  A method used for computing Geary autocorrelation descriptors based on 8 properties of AADs.     Usage:     result=CalculateGearyAutoTotal(protein)     Input: protein is a pure protein sequence.     Output: result is a dict form containing 30\*8=240 Geary     autocorrelation descriptors based on the given properties(i.e., \_AAPropert).  ####################################################################################  **CalculateMoranAuto**(ProteinSequence, AAProperty, AAPropertyName)  ####################################################################################  A method used for computing MoranAuto for all properties     Usage:     result=CalculateMoranAuto(protein,AAP,AAPName)     Input: protein is a pure protein sequence.     AAProperty is a list or tuple form containing the properties of 20 amino acids (e.g., \_AAProperty).     AAPName is a list or tuple form used for indicating the property (e.g., '\_AAPropertyName').      Output: result is a dict form containing 30\*p Moran autocorrelation     descriptors based on the given properties.  ####################################################################################  **CalculateMoranAutoAvFlexibility**(ProteinSequence)  ####################################################################################  Calculte the MoranAuto Autocorrelation descriptors based on     AvFlexibility.     Usage:     result=CalculateMoranAutoAvFlexibility(protein)     Input: protein is a pure protein sequence.     Output: result is a dict form containing 30 Moran Autocorrelation     descriptors based on AvFlexibility.  ####################################################################################  **CalculateMoranAutoFreeEnergy**(ProteinSequence)  ####################################################################################  Calculte the MoranAuto Autocorrelation descriptors based on     FreeEnergy.     Usage:     result=CalculateMoranAutoFreeEnergy(protein)     Input: protein is a pure protein sequence.     Output: result is a dict form containing 30 Moran Autocorrelation     descriptors based on FreeEnergy.  ####################################################################################  **CalculateMoranAutoHydrophobicity**(ProteinSequence)  ####################################################################################  Calculte the MoranAuto Autocorrelation descriptors based on hydrophobicity.     Usage:     result=CalculateMoranAutoHydrophobicity(protein)     Input: protein is a pure protein sequence.     Output: result is a dict form containing 30 Moran Autocorrelation     descriptors based on hydrophobicity.  ####################################################################################  **CalculateMoranAutoMutability**(ProteinSequence)  ####################################################################################  Calculte the MoranAuto Autocorrelation descriptors based on     Mutability.     Usage:     result=CalculateMoranAutoMutability(protein)     Input: protein is a pure protein sequence.     Output: result is a dict form containing 30 Moran Autocorrelation     descriptors based on Mutability.  ####################################################################################  **CalculateMoranAutoPolarizability**(ProteinSequence)  ####################################################################################  Calculte the MoranAuto Autocorrelation descriptors based on     Polarizability.     Usage:     result=CalculateMoranAutoPolarizability(protein)     Input: protein is a pure protein sequence.     Output: result is a dict form containing 30 Moran Autocorrelation     descriptors based on Polarizability.  ####################################################################################  **CalculateMoranAutoResidueASA**(ProteinSequence)  ####################################################################################  Calculte the MoranAuto Autocorrelation descriptors based on     ResidueASA.     Usage:     result=CalculateMoranAutoResidueASA(protein)     Input: protein is a pure protein sequence.     Output: result is a dict form containing 30 Moran Autocorrelation     descriptors based on ResidueASA.  ####################################################################################  **CalculateMoranAutoResidueVol**(ProteinSequence)  ####################################################################################  Calculte the MoranAuto Autocorrelation descriptors based on     ResidueVol.     Usage:     result=CalculateMoranAutoResidueVol(protein)     Input: protein is a pure protein sequence.     Output: result is a dict form containing 30 Moran Autocorrelation     descriptors based on ResidueVol.  ####################################################################################  **CalculateMoranAutoSteric**(ProteinSequence)  ####################################################################################  Calculte the MoranAuto Autocorrelation descriptors based on     AutoSteric.     Usage:     result=CalculateMoranAutoSteric(protein)     Input: protein is a pure protein sequence.     Output: result is a dict form containing 30 Moran Autocorrelation     descriptors based on AutoSteric.  ####################################################################################  **CalculateMoranAutoTotal**(ProteinSequence)  ####################################################################################  A method used for computing Moran autocorrelation descriptors based on 8 properties of AADs.     Usage:     result=CalculateMoranAutoTotal(protein)     Input: protein is a pure protein sequence.     Output: result is a dict form containing 30\*8=240 Moran     autocorrelation descriptors based on the given properties(i.e., \_AAPropert).  ####################################################################################  **CalculateNormalizedMoreauBrotoAuto**(ProteinSequence, AAProperty, AAPropertyName)  ####################################################################################  A method used for computing MoreauBrotoAuto for all properties.     Usage:     result=CalculateNormalizedMoreauBrotoAuto(protein,AAP,AAPName)     Input: protein is a pure protein sequence.     AAProperty is a list or tuple form containing the properties of 20 amino acids (e.g., \_AAProperty).     AAPName is a list or tuple form used for indicating the property (e.g., '\_AAPropertyName').      Output: result is a dict form containing 30\*p Normalized Moreau-Broto autocorrelation     descriptors based on the given properties.  ####################################################################################  **CalculateNormalizedMoreauBrotoAutoAvFlexibility**(ProteinSequence)  ####################################################################################  Calculte the NormalizedMoreauBorto Autocorrelation descriptors based on     AvFlexibility.     Usage:     result=CalculateNormalizedMoreauBrotoAutoAvFlexibility(protein)     Input: protein is a pure protein sequence.     Output: result is a dict form containing 30 Normalized Moreau-Broto Autocorrelation     descriptors based on AvFlexibility.  ####################################################################################  **CalculateNormalizedMoreauBrotoAutoFreeEnergy**(ProteinSequence)  ####################################################################################  Calculte the NormalizedMoreauBorto Autocorrelation descriptors based on     FreeEnergy.     Usage:     result=CalculateNormalizedMoreauBrotoAutoFreeEnergy(protein)     Input: protein is a pure protein sequence.     Output: result is a dict form containing 30 Normalized Moreau-Broto Autocorrelation     descriptors based on FreeEnergy.  ####################################################################################  **CalculateNormalizedMoreauBrotoAutoHydrophobicity**(ProteinSequence)  ####################################################################################  Calculte the NormalizedMoreauBorto Autocorrelation descriptors based on     hydrophobicity.     Usage:     result=CalculateNormalizedMoreauBrotoAutoHydrophobicity(protein)     Input: protein is a pure protein sequence.     Output: result is a dict form containing 30 Normalized Moreau-Broto Autocorrelation     descriptors based on Hydrophobicity.  ####################################################################################  **CalculateNormalizedMoreauBrotoAutoMutability**(ProteinSequence)  ####################################################################################  Calculte the NormalizedMoreauBorto Autocorrelation descriptors based on Mutability.     Usage:     result=CalculateNormalizedMoreauBrotoAutoMutability(protein)     Input: protein is a pure protein sequence.     Output: result is a dict form containing 30 Normalized Moreau-Broto Autocorrelation     descriptors based on Mutability.  ####################################################################################  **CalculateNormalizedMoreauBrotoAutoPolarizability**(ProteinSequence)  ####################################################################################  Calculte the NormalizedMoreauBorto Autocorrelation descriptors based on     Polarizability.     Usage:     result=CalculateNormalizedMoreauBrotoAutoPolarizability(protein)     Input: protein is a pure protein sequence.     Output: result is a dict form containing 30 Normalized Moreau-Broto Autocorrelation     descriptors based on Polarizability.  ####################################################################################  **CalculateNormalizedMoreauBrotoAutoResidueASA**(ProteinSequence)  ####################################################################################  Calculte the NormalizedMoreauBorto Autocorrelation descriptors based on     ResidueASA.     Usage:     result=CalculateNormalizedMoreauBrotoAutoResidueASA(protein)     Input: protein is a pure protein sequence.     Output: result is a dict form containing 30 Normalized Moreau-Broto Autocorrelation     descriptors based on ResidueASA.  ####################################################################################  **CalculateNormalizedMoreauBrotoAutoResidueVol**(ProteinSequence)  ####################################################################################  Calculte the NormalizedMoreauBorto Autocorrelation descriptors based on     ResidueVol.     Usage:     result=CalculateNormalizedMoreauBrotoAutoResidueVol(protein)     Input: protein is a pure protein sequence.     Output: result is a dict form containing 30 Normalized Moreau-Broto Autocorrelation     descriptors based on ResidueVol.  ####################################################################################  **CalculateNormalizedMoreauBrotoAutoSteric**(ProteinSequence)  ####################################################################################  Calculte the NormalizedMoreauBorto Autocorrelation descriptors based on Steric.     Usage:     result=CalculateNormalizedMoreauBrotoAutoSteric(protein)     Input: protein is a pure protein sequence.     Output: result is a dict form containing 30 Normalized Moreau-Broto Autocorrelation     descriptors based on Steric.  ####################################################################################  **CalculateNormalizedMoreauBrotoAutoTotal**(ProteinSequence)  ####################################################################################  A method used for computing normalized Moreau Broto autocorrelation descriptors based      on 8 proterties of AADs.     Usage:     result=CalculateNormalizedMoreauBrotoAutoTotal(protein)     Input: protein is a pure protein sequence.     Output: result is a dict form containing 30\*8=240 normalized Moreau Broto      autocorrelation descriptors based on the given properties(i.e., \_AAPropert).  ####################################################################################  **NormalizeEachAAP**(AAP)  ####################################################################################  All of the amino acid indices are centralized and      standardized before the calculation.     Usage:     result=NormalizeEachAAP(AAP)     Input: AAP is a dict form containing the properties of 20 amino acids.     Output: result is the a dict form containing the normalized properties      of 20 amino acids.  #################################################################################### |

|  |  |  |
| --- | --- | --- |
| **Data** | | |
|  |  | **AALetter** = ['A', 'R', 'N', 'D', 'C', 'Q', 'E', 'G', 'H', 'I', 'L', 'K', 'M', 'F', 'P', 'S', 'T', 'W', 'Y', 'V'] |
