# Supplementary material for: Protein language model-based prediction for plant miRNA encoded peptides
Source: PeerJ Comput Sci. 2025 Mar 18;11:e2733. doi: 10.7717/peerj-cs.2733 (PMC11935769; doi:10.7717/peerj-cs.2733)
Supplement: Supplemental Information 1 [file peerj-cs-11-2733-s001.zip › pLM4PEP-master/pydpi/html/CTD.html]

Python: module CTD

|  |  |
| --- | --- |
| **CTD** | index /home/orient/ProPy/CTD.py |

#####################################################################################  
   
This module is used for computing the composition, transition and distribution   
   
descriptors based on the different properties of AADs. The AADs with the same   
   
properties is marked as the same number. You can get 147 descriptors for a given  
   
protein sequence. You can freely use and distribute it. If you hava  any problem,   
   
you could contact with us timely!  
   
References:  
   
[1]: Inna Dubchak, Ilya Muchink, Stephen R.Holbrook and Sung-Hou Kim. Prediction   
   
of protein folding class using global description of amino acid sequence. Proc.Natl.  
   
Acad.Sci.USA, 1995, 92, 8700-8704.  
   
[2]:Inna Dubchak, Ilya Muchink, Christopher Mayor, Igor Dralyuk and Sung-Hou Kim.   
   
Recognition of a Protein Fold in the Context of the SCOP classification. Proteins:   
   
Structure, Function and Genetics,1999,35,401-407.  
   
Authors: Dongsheng Cao and Yizeng Liang.  
   
Date: 2010.11.22  
   
Email: oriental-cds@163.com  
   
#####################################################################################

**Modules**

|  |  |  |  |
| --- | --- | --- | --- |
| copy | math | string |  |

|  |  |  |
| --- | --- | --- |
| **Functions** | | |
|  |  | **CalculateC**(ProteinSequence)  ###############################################################################################  Calculate all composition descriptors based seven different properties of AADs.     Usage:     result=CalculateC(protein)     Input:protein is a pure protein sequence.     Output:result is a dict form containing all composition descriptors.  ###############################################################################################  **CalculateCTD**(ProteinSequence)  ###############################################################################################  Calculate all CTD descriptors based seven different properties of AADs.     Usage:     result=CalculateCTD(protein)     Input:protein is a pure protein sequence.     Output:result is a dict form containing all CTD descriptors.  ###############################################################################################  **CalculateComposition**(ProteinSequence, AAProperty, AAPName)  ###############################################################################################  A method used for computing composition descriptors.     Usage:     result=CalculateComposition(protein,AAProperty,AAPName)     Input: protein is a pure protein sequence.     AAProperty is a dict form containing classifciation of amino acids such as \_Polarizability.     AAPName is a string used for indicating a AAP name.     Output: result is a dict form containing composition descriptors based on the given property.  ###############################################################################################  **CalculateCompositionCharge**(ProteinSequence)  ###############################################################################################  A method used for calculating composition descriptors based on Charge of      AADs.     Usage:      result=CalculateCompositionCharge(protein)     Input:protein is a pure protein sequence.     Output:result is a dict form containing Composition descriptors based on Charge.  ###############################################################################################  **CalculateCompositionHydrophobicity**(ProteinSequence)  ###############################################################################################  A method used for calculating composition descriptors based on Hydrophobicity of      AADs.     Usage:      result=CalculateCompositionHydrophobicity(protein)     Input:protein is a pure protein sequence.     Output:result is a dict form containing Composition descriptors based on Hydrophobicity.  ###############################################################################################  **CalculateCompositionNormalizedVDWV**(ProteinSequence)  ###############################################################################################  A method used for calculating composition descriptors based on NormalizedVDWV of      AADs.     Usage:      result=CalculateCompositionNormalizedVDWV(protein)     Input:protein is a pure protein sequence.     Output:result is a dict form containing Composition descriptors based on NormalizedVDWV.  ###############################################################################################  **CalculateCompositionPolarity**(ProteinSequence)  ###############################################################################################  A method used for calculating composition descriptors based on Polarity of      AADs.     Usage:      result=CalculateCompositionPolarity(protein)     Input:protein is a pure protein sequence.     Output:result is a dict form containing Composition descriptors based on Polarity.  ###############################################################################################  **CalculateCompositionPolarizability**(ProteinSequence)  ###############################################################################################  A method used for calculating composition descriptors based on Polarizability of      AADs.     Usage:      result=CalculateCompositionPolarizability(protein)     Input:protein is a pure protein sequence.     Output:result is a dict form containing Composition descriptors based on Polarizability.  ###############################################################################################  **CalculateCompositionSecondaryStr**(ProteinSequence)  ###############################################################################################  A method used for calculating composition descriptors based on SecondaryStr of      AADs.     Usage:      result=CalculateCompositionSecondaryStr(protein)     Input:protein is a pure protein sequence.     Output:result is a dict form containing Composition descriptors based on SecondaryStr.  ###############################################################################################  **CalculateCompositionSolventAccessibility**(ProteinSequence)  ###############################################################################################  A method used for calculating composition descriptors based on SolventAccessibility     of  AADs.     Usage:      result=CalculateCompositionSolventAccessibility(protein)     Input:protein is a pure protein sequence.     Output:result is a dict form containing Composition descriptors based on SolventAccessibility.  ###############################################################################################  **CalculateD**(ProteinSequence)  ###############################################################################################  Calculate all distribution descriptors based seven different properties of AADs.     Usage:     result=CalculateD(protein)     Input:protein is a pure protein sequence.     Output:result is a dict form containing all distribution descriptors.  ###############################################################################################  **CalculateDistribution**(ProteinSequence, AAProperty, AAPName)  ###############################################################################################  A method used for computing distribution descriptors.     Usage:     result=CalculateDistribution(protein,AAProperty,AAPName)     Input:protein is a pure protein sequence.     AAProperty is a dict form containing classifciation of amino acids such as \_Polarizability.     AAPName is a string used for indicating a AAP name.     Output:result is a dict form containing Distribution descriptors based on the given property.  ###############################################################################################  **CalculateDistributionCharge**(ProteinSequence)  ###############################################################################################  A method used for calculating Distribution descriptors based on Charge of      AADs.     Usage:      result=CalculateDistributionCharge(protein)     Input:protein is a pure protein sequence.     Output:result is a dict form containing Distribution descriptors based on Charge.  ###############################################################################################  **CalculateDistributionHydrophobicity**(ProteinSequence)  ###############################################################################################  A method used for calculating Distribution descriptors based on Hydrophobicity of      AADs.     Usage:      result=CalculateDistributionHydrophobicity(protein)     Input:protein is a pure protein sequence.     Output:result is a dict form containing Distribution descriptors based on Hydrophobicity.  ###############################################################################################  **CalculateDistributionNormalizedVDWV**(ProteinSequence)  ###############################################################################################  A method used for calculating Distribution descriptors based on NormalizedVDWV of      AADs.     Usage:      result=CalculateDistributionNormalizedVDWV(protein)     Input:protein is a pure protein sequence.     Output:result is a dict form containing Distribution descriptors based on NormalizedVDWV.  ###############################################################################################  **CalculateDistributionPolarity**(ProteinSequence)  ###############################################################################################  A method used for calculating Distribution descriptors based on Polarity of      AADs.     Usage:      result=CalculateDistributionPolarity(protein)     Input:protein is a pure protein sequence.     Output:result is a dict form containing Distribution descriptors based on Polarity.  ###############################################################################################  **CalculateDistributionPolarizability**(ProteinSequence)  ###############################################################################################  A method used for calculating Distribution descriptors based on Polarizability of      AADs.     Usage:      result=CalculateDistributionPolarizability(protein)     Input:protein is a pure protein sequence.     Output:result is a dict form containing Distribution descriptors based on Polarizability.  ###############################################################################################  **CalculateDistributionSecondaryStr**(ProteinSequence)  ###############################################################################################  A method used for calculating Distribution descriptors based on SecondaryStr of      AADs.     Usage:      result=CalculateDistributionSecondaryStr(protein)     Input:protein is a pure protein sequence.     Output:result is a dict form containing Distribution descriptors based on SecondaryStr.  ###############################################################################################  **CalculateDistributionSolventAccessibility**(ProteinSequence)  ###############################################################################################  A method used for calculating Distribution descriptors based on SolventAccessibility     of  AADs.     Usage:      result=CalculateDistributionSolventAccessibility(protein)     Input:protein is a pure protein sequence.     Output:result is a dict form containing Distribution descriptors based on SolventAccessibility.  ###############################################################################################  **CalculateT**(ProteinSequence)  ###############################################################################################  Calculate all transition descriptors based seven different properties of AADs.     Usage:     result=CalculateT(protein)     Input:protein is a pure protein sequence.     Output:result is a dict form containing all transition descriptors.  ###############################################################################################  **CalculateTransition**(ProteinSequence, AAProperty, AAPName)  ###############################################################################################  A method used for computing transition descriptors     Usage:     result=CalculateTransition(protein,AAProperty,AAPName)     Input:protein is a pure protein sequence.     AAProperty is a dict form containing classifciation of amino acids such as \_Polarizability.     AAPName is a string used for indicating a AAP name.     Output:result is a dict form containing transition descriptors based on the given property.  ###############################################################################################  **CalculateTransitionCharge**(ProteinSequence)  ###############################################################################################  A method used for calculating Transition descriptors based on Charge of      AADs.     Usage:      result=CalculateTransitionCharge(protein)     Input:protein is a pure protein sequence.     Output:result is a dict form containing Transition descriptors based on Charge.  ###############################################################################################  **CalculateTransitionHydrophobicity**(ProteinSequence)  ###############################################################################################  A method used for calculating Transition descriptors based on Hydrophobicity of      AADs.     Usage:      result=CalculateTransitionHydrophobicity(protein)     Input:protein is a pure protein sequence.     Output:result is a dict form containing Transition descriptors based on Hydrophobicity.  ###############################################################################################  **CalculateTransitionNormalizedVDWV**(ProteinSequence)  ###############################################################################################  A method used for calculating Transition descriptors based on NormalizedVDWV of      AADs.     Usage:      result=CalculateTransitionNormalizedVDWV(protein)     Input:protein is a pure protein sequence.     Output:result is a dict form containing Transition descriptors based on NormalizedVDWV.  ###############################################################################################  **CalculateTransitionPolarity**(ProteinSequence)  ###############################################################################################  A method used for calculating Transition descriptors based on Polarity of      AADs.     Usage:      result=CalculateTransitionPolarity(protein)     Input:protein is a pure protein sequence.     Output:result is a dict form containing Transition descriptors based on Polarity.  ###############################################################################################  **CalculateTransitionPolarizability**(ProteinSequence)  ###############################################################################################  A method used for calculating Transition descriptors based on Polarizability of      AADs.     Usage:      result=CalculateTransitionPolarizability(protein)     Input:protein is a pure protein sequence.     Output:result is a dict form containing Transition descriptors based on Polarizability.  ###############################################################################################  **CalculateTransitionSecondaryStr**(ProteinSequence)  ###############################################################################################  A method used for calculating Transition descriptors based on SecondaryStr of      AADs.     Usage:      result=CalculateTransitionSecondaryStr(protein)     Input:protein is a pure protein sequence.     Output:result is a dict form containing Transition descriptors based on SecondaryStr.  ###############################################################################################  **CalculateTransitionSolventAccessibility**(ProteinSequence)  ###############################################################################################  A method used for calculating Transition descriptors based on SolventAccessibility     of  AADs.     Usage:      result=CalculateTransitionSolventAccessibility(protein)     Input:protein is a pure protein sequence.     Output:result is a dict form containing Transition descriptors based on SolventAccessibility.  ###############################################################################################  **StringtoNum**(ProteinSequence, AAProperty)  ###############################################################################################  Tranform the protein sequence into the string form such as 32123223132121123.     Usage:     result=StringtoNum(protein,AAProperty)     Input: protein is a pure protein sequence.     AAProperty is a dict form containing classifciation of amino acids such as \_Polarizability.     Output: result is a string such as 123321222132111123222  ############################################################################################### |

|  |  |  |
| --- | --- | --- |
| **Data** | | |
|  |  | **AALetter** = ['A', 'R', 'N', 'D', 'C', 'E', 'Q', 'G', 'H', 'I', 'L', 'K', 'M', 'F', 'P', 'S', 'T', 'W', 'Y', 'V'] |
