# Supplementary material for: Protein language model-based prediction for plant miRNA encoded peptides
Source: PeerJ Comput Sci. 2025 Mar 18;11:e2733. doi: 10.7717/peerj-cs.2733 (PMC11935769; doi:10.7717/peerj-cs.2733)
Supplement: Supplemental Information 1 [file peerj-cs-11-2733-s001.zip › pLM4PEP-master/pydpi/html/ConjointTriad.html]

Python: module ConjointTriad

|  |  |
| --- | --- |
| **ConjointTriad** | index f:\pydpi\protein\conjointtriad.py |

###############################################################################  
This module is used for calculating the conjoint triad features only from the   
   
protein sequence information. You can get 7\*7\*7=343 features.You can freely   
   
use and distribute it. If you hava any problem, you could contact with us timely!  
   
Reference:  
   
Juwen Shen, Jian Zhang, Xiaomin Luo, Weiliang Zhu, Kunqian Yu, Kaixian Chen,   
   
Yixue Li, Huanliang Jiang. Predicting proten-protein interactions based only   
   
on sequences inforamtion. PNAS. 2007 (104) 4337-4341.  
   
Authors: Dongsheng Cao and Yizeng Liang.  
   
Date: 2012.09.18  
   
Email: oriental-cds@163.com  
   
###############################################################################

**Modules**

|  |  |  |  |
| --- | --- | --- | --- |
| string |  |  |  |

|  |  |  |
| --- | --- | --- |
| **Functions** | | |
|  |  | **CalculateConjointTriad**(proteinsequence)  Calculate the conjoint triad features from protein sequence.     Useage:     res = CalculateConjointTriad(protein)     Input: protein is a pure protein sequence.     Output is a dict form containing all 343 conjoint triad features. |

|  |  |  |
| --- | --- | --- |
| **Data** | | |
|  |  | **AALetter** = ['A', 'R', 'N', 'D', 'C', 'E', 'Q', 'G', 'H', 'I', 'L', 'K', 'M', 'F', 'P', 'S', 'T', 'W', 'Y', 'V'] |
