# Supplementary material for: Protein language model-based prediction for plant miRNA encoded peptides
Source: PeerJ Comput Sci. 2025 Mar 18;11:e2733. doi: 10.7717/peerj-cs.2733 (PMC11935769; doi:10.7717/peerj-cs.2733)
Supplement: Supplemental Information 1 [file peerj-cs-11-2733-s001.zip › pLM4PEP-master/pydpi/html/GetProteinFromUniprot.html]

Python: module GetProteinFromUniprot

|  |  |
| --- | --- |
| **GetProteinFromUniprot** | index /home/orient/ProPy/GetProteinFromUniprot.py |

################################################################################################  
   
This module is used to download the protein sequence from the uniprot (http://www.uniprot.org/)   
   
website. You can only need input a protein ID or prepare a file (ID.txt) related to ID. You can  
   
 obtain a .txt (ProteinSequence.txt) file saving protein sequence you need.  You can freely use   
   
 and distribute it. If you hava  any problem, you could contact with us timely!  
   
Authors: Dongsheng Cao and Yizeng Liang.  
   
Date: 2012.9.3  
   
Email: oriental-cds@163.com  
   
################################################################################################

**Modules**

|  |  |  |  |
| --- | --- | --- | --- |
| string | urllib |  |  |

|  |  |  |
| --- | --- | --- |
| **Functions** | | |
|  |  | **GetProteinSequence**(ProteinID)  #########################################################################################  Get the protein sequence from the uniprot website by ID.     Usage:     result=GetProteinSequence(ProteinID)     Input: ProteinID is a string indicating ID such as "P48039".     Output: result is a protein sequence.  #########################################################################################  **GetProteinSequenceFromTxt**(path, openfile, savefile)  #########################################################################################  Get the protein sequence from the uniprot website by the file containing ID.     Usage:      result=GetProteinSequenceFromTxt(path,openfile,savefile)     Input: path is a directory path containing the ID file such as "/home/orient/protein/"      openfile is the ID file such as "proteinID.txt"     savefile is the file saving the obtained protein sequences such as "protein.txt"  ######################################################################################### |
