# Supplementary material for: Protein language model-based prediction for plant miRNA encoded peptides
Source: PeerJ Comput Sci. 2025 Mar 18;11:e2733. doi: 10.7717/peerj-cs.2733 (PMC11935769; doi:10.7717/peerj-cs.2733)
Supplement: Supplemental Information 1 [file peerj-cs-11-2733-s001.zip › pLM4PEP-master/pydpi/html/GetSubSeq.html]

Python: module GetSubSeq

|  |  |
| --- | --- |
| **GetSubSeq** | index /home/orient/ProPy/GetSubSeq.py |

#####################################################################################  
   
The prediction of functional sites (e.g.,methylation) of proteins usually needs to   
   
split the total protein into a set of segments around specific amino acid. Given a   
   
specific window size p, we can obtain all segments of length equal to (2\*p+1) very   
   
easily. Note that the output of the method is a list form. You can freely use and   
   
distribute it. If you have any problem, you could contact with us timely.  
   
Authors: Dongsheng Cao and Yizeng Liang.  
   
Date: 2012.9.2  
   
Email: oriental-cds@163.com  
   
#####################################################################################

**Modules**

|  |  |  |  |
| --- | --- | --- | --- |
| re | string |  |  |

|  |  |  |
| --- | --- | --- |
| **Functions** | | |
|  |  | **GetSubSequence**(ProteinSequence, ToAA='S', window=3)  #######################################################################  Get all 2\*window+1 sub-sequences whose cener is ToAA in a protein.     Usage:     result=GetSubSequence(protein,ToAA,window)     Input:protein is a pure problem sequence.     ToAA is the central (query point) amino acid in the sub-sequence.     window is the span.     result is a list form containing all satisfied sub-sequences.  ####################################################################### |

|  |  |  |
| --- | --- | --- |
| **Data** | | |
|  |  | **AALetter** = ['A', 'R', 'N', 'D', 'C', 'E', 'Q', 'G', 'H', 'I', 'L', 'K', 'M', 'F', 'P', 'S', 'T', 'W', 'Y', 'V'] |
