# Supplementary material for: Protein language model-based prediction for plant miRNA encoded peptides
Source: PeerJ Comput Sci. 2025 Mar 18;11:e2733. doi: 10.7717/peerj-cs.2733 (PMC11935769; doi:10.7717/peerj-cs.2733)
Supplement: Supplemental Information 1 [file peerj-cs-11-2733-s001.zip › pLM4PEP-master/pydpi/html/PseudoAAC.html]

Python: module PseudoAAC

|  |  |
| --- | --- |
| **PseudoAAC** | index /home/orient/ProPy/PseudoAAC.py |

#########################################################################################  
   
Instead of using the conventional 20-D amino acid composition to represent the sample  
   
of a protein, Prof. Kuo-Chen Chou proposed the pseudo amino acid (PseAA) composition   
   
in order for inluding the sequence-order information. Based on the concept of Chou's   
   
pseudo amino acid composition, the server PseAA was designed in a flexible way, allowing   
   
users to generate various kinds of pseudo amino acid composition for a given protein  
   
sequence by selecting different parameters and their combinations. This module aims at   
   
computing two types of PseAA descriptors: Type I and Type II.   
   
You can freely use and distribute it. If you have any problem, you could contact   
   
with us timely.  
   
References:  
   
[1]: Kuo-Chen Chou. Prediction of Protein Cellular Attributes Using Pseudo-Amino Acid   
   
Composition. PROTEINS: Structure, Function, and Genetics, 2001, 43: 246-255.  
   
[2]: http://www.csbio.sjtu.edu.cn/bioinf/PseAAC/  
   
[3]: http://www.csbio.sjtu.edu.cn/bioinf/PseAAC/type2.htm  
   
[4]: Kuo-Chen Chou. Using amphiphilic pseudo amino acid composition to predict enzyme   
   
subfamily classes. Bioinformatics, 2005,21,10-19.  
   
Authors: Dongsheng Cao and Yizeng Liang.  
   
Date: 2012.9.2  
   
Email: oriental-cds@163.com  
   
   
The hydrophobicity values are from JACS, 1962, 84: 4240-4246. (C. Tanford).  
   
The hydrophilicity values are from PNAS, 1981, 78:3824-3828 (T.P.Hopp & K.R.Woods).  
   
The side-chain mass for each of the 20 amino acids.  
   
CRC Handbook of Chemistry and Physics, 66th ed., CRC Press, Boca Raton, Florida (1985).  
   
R.M.C. Dawson, D.C. Elliott, W.H. Elliott, K.M. Jones, Data for Biochemical Research 3rd ed.,   
   
Clarendon Press Oxford (1986).  
   
#########################################################################################

**Modules**

|  |  |  |  |
| --- | --- | --- | --- |
| math | string |  |  |

|  |  |  |
| --- | --- | --- |
| **Functions** | | |
|  |  | **GetAAComposition**(ProteinSequence)  ########################################################################################  Calculate the composition of Amino acids      for a given protein sequence.     Usage:     result=CalculateAAComposition(protein)     Input: protein is a pure protein sequence.     Output: result is a dict form containing the composition of      20 amino acids.  ########################################################################################  **GetAPseudoAAC**(ProteinSequence, lamda=10, weight=0.5)  #######################################################################################  Computing all of type II pseudo-amino acid compostion descriptors based on the given      properties. Note that the number of PAAC strongly depends on the lamda value. if lamda      = 20, we can obtain 20+20=40 PAAC descriptors. The size of these values depends on the      choice of lamda and weight simultaneously.     Usage:     result=GetAPseudoAAC(protein,lamda,weight)     Input: protein is a pure protein sequence.     lamda factor reflects the rank of correlation and is a non-Negative integer, such as 15.     Note that (1)lamda should NOT be larger than the length of input protein sequence;     (2) lamda must be non-Negative integer, such as 0, 1, 2, ...; (3) when lamda =0, the      output of PseAA server is the 20-D amino acid composition.     weight factor is designed for the users to put weight on the additional PseAA components      with respect to the conventional AA components. The user can select any value within the      region from 0.05 to 0.7 for the weight factor.     Output: result is a dict form containing calculated 20+lamda PAAC descriptors.  #######################################################################################  **GetAPseudoAAC1**(ProteinSequence, lamda=10, weight=0.5)  ########################################################################################  Computing the first 20 of type II pseudo-amino acid compostion descriptors based on     [\_Hydrophobicity,\_hydrophilicity].  ########################################################################################  **GetAPseudoAAC2**(ProteinSequence, lamda=10, weight=0.5)  #######################################################################################  Computing the last lamda of type II pseudo-amino acid compostion descriptors based on     [\_Hydrophobicity,\_hydrophilicity].  #######################################################################################  **GetCorrelationFunction**(Ri='S', Rj='D', AAP=[])  ########################################################################################  Computing the correlation between two given amino acids using the given     properties.     Usage:     result=GetCorrelationFunction(Ri,Rj,AAP)     Input: Ri and Rj are the amino acids, respectively.     AAP is a list form containing the properties, each of which is a dict form.     Output: result is the correlation value between two amino acids.  ########################################################################################  **GetPseudoAAC**(ProteinSequence, lamda=10, weight=0.05, AAP=[])  #######################################################################################  Computing all of type I pseudo-amino acid compostion descriptors based on the given      properties. Note that the number of PAAC strongly depends on the lamda value. if lamda      = 20, we can obtain 20+20=40 PAAC descriptors. The size of these values depends on the      choice of lamda and weight simultaneously. You must specify some properties into AAP.     Usage:     result=GetPseudoAAC(protein,lamda,weight)     Input: protein is a pure protein sequence.     lamda factor reflects the rank of correlation and is a non-Negative integer, such as 15.     Note that (1)lamda should NOT be larger than the length of input protein sequence;     (2) lamda must be non-Negative integer, such as 0, 1, 2, ...; (3) when lamda =0, the      output of PseAA server is the 20-D amino acid composition.     weight factor is designed for the users to put weight on the additional PseAA components      with respect to the conventional AA components. The user can select any value within the      region from 0.05 to 0.7 for the weight factor.     AAP is a list form containing the properties, each of which is a dict form.     Output: result is a dict form containing calculated 20+lamda PAAC descriptors.  ########################################################################################  **GetPseudoAAC1**(ProteinSequence, lamda=10, weight=0.05, AAP=[])  #######################################################################################  Computing the first 20 of type I pseudo-amino acid compostion descriptors based on the given      properties.  ########################################################################################  **GetPseudoAAC2**(ProteinSequence, lamda=10, weight=0.05, AAP=[])  #######################################################################################  Computing the last lamda of type I pseudo-amino acid compostion descriptors based on the given      properties.  ########################################################################################  **GetSequenceOrderCorrelationFactor**(ProteinSequence, k=1, AAP=[])  ########################################################################################  Computing the Sequence order correlation factor with gap equal to k based on      the given properities.     Usage:     result=GetSequenceOrderCorrelationFactor(protein,k,AAP)     Input: protein is a pure protein sequence.     k is the gap.     AAP is a list form containing the properties, each of which is a dict form.     Output: result is the correlation factor value with the gap equal to k.  ########################################################################################  **GetSequenceOrderCorrelationFactorForAPAAC**(ProteinSequence, k=1)  ########################################################################################  Computing the Sequence order correlation factor with gap equal to k based on      [\_Hydrophobicity,\_hydrophilicity] for APAAC (type II PseAAC) .     Usage:     result=GetSequenceOrderCorrelationFactorForAPAAC(protein,k)     Input: protein is a pure protein sequence.     k is the gap.     Output: result is the correlation factor value with the gap equal to k.  ########################################################################################  **NormalizeEachAAP**(AAP)  ########################################################################################  All of the amino acid indices are centralized and      standardized before the calculation.     Usage:     result=NormalizeEachAAP(AAP)     Input: AAP is a dict form containing the properties of 20 amino acids.     Output: result is the a dict form containing the normalized properties      of 20 amino acids.  ######################################################################################## |

|  |  |  |
| --- | --- | --- |
| **Data** | | |
|  |  | **AALetter** = ['A', 'R', 'N', 'D', 'C', 'E', 'Q', 'G', 'H', 'I', 'L', 'K', 'M', 'F', 'P', 'S', 'T', 'W', 'Y', 'V'] |
