# Supplementary material for: Protein language model-based prediction for plant miRNA encoded peptides
Source: PeerJ Comput Sci. 2025 Mar 18;11:e2733. doi: 10.7717/peerj-cs.2733 (PMC11935769; doi:10.7717/peerj-cs.2733)
Supplement: Supplemental Information 1 [file peerj-cs-11-2733-s001.zip › pLM4PEP-master/pydpi/html/QuasiSequenceOrder.html]

Python: module QuasiSequenceOrder

|  |  |
| --- | --- |
| **QuasiSequenceOrder** | index /home/orient/ProPy/propy/QuasiSequenceOrder.py |

##############################################################################################  
This module is used for computing the quasi sequence order descriptors based on the   
   
given protein sequence. We can obtain two types of descriptors: Sequence-order-coupling  
   
number and quasi-sequence-order descriptors. Two distance matrixes between 20 amino acids  
   
are employed. You can freely use and distribute it. If you have any problem, please contact  
   
us immediately.  
   
References:  
   
[1]:Kuo-Chen Chou. Prediction of Protein Subcellar Locations by Incorporating  
   
Quasi-Sequence-Order Effect. Biochemical and Biophysical Research Communications   
   
2000, 278, 477-483.  
   
[2]: Kuo-Chen Chou and Yu-Dong Cai. Prediction of Protein sucellular locations by  
   
GO-FunD-PseAA predictor, Biochemical and Biophysical Research Communications,  
   
2004, 320, 1236-1239.  
   
[3]:Gisbert Schneider and Paul wrede. The Rational Design of Amino Acid  
   
Sequences by Artifical Neural Networks and Simulated Molecular Evolution: Do  
   
Novo Design of an Idealized Leader Cleavge Site. Biophys Journal, 1994, 66,  
   
335-344.  
   
Authors: Dongsheng Cao and Yizeng Liang.  
   
Date: 2012.09.03  
   
Email: oriental-cds@163.com  
   
##############################################################################################

**Modules**

|  |  |  |  |
| --- | --- | --- | --- |
| math | string |  |  |

|  |  |  |
| --- | --- | --- |
| **Functions** | | |
|  |  | **GetAAComposition**(ProteinSequence)  ###############################################################################  Calculate the composition of Amino acids      for a given protein sequence.     Usage:     result=CalculateAAComposition(protein)     Input: protein is a pure protein sequence.     Output: result is a dict form containing the composition of      20 amino acids.  ###############################################################################  **GetQuasiSequenceOrder**(ProteinSequence, maxlag=30, weight=0.1)  ###############################################################################  Computing quasi-sequence-order descriptors for a given protein.     [1]:Kuo-Chen Chou. Prediction of Protein Subcellar Locations by      Incorporating Quasi-Sequence-Order Effect. Biochemical and Biophysical     Research Communications 2000, 278, 477-483.     Usage:     result = GetQuasiSequenceOrder(protein,maxlag,weight)     Input: protein is a pure protein sequence     maxlag is the maximum lag and the length of the protein should be larger     than maxlag. default is 30.     weight is a weight factor.  please see reference 1 for its choice.     Output: result is a dict form containing all quasi-sequence-order descriptors  ###############################################################################  **GetQuasiSequenceOrder1**(ProteinSequence, maxlag=30, weight=0.1)  ###############################################################################  Computing the first 20 quasi-sequence-order descriptors for      a given protein sequence.     Usage:     result = GetQuasiSequenceOrder1(protein,maxlag,weigt)     see method GetQuasiSequenceOrder for the choice of parameters.  ###############################################################################  **GetQuasiSequenceOrder2**(ProteinSequence, maxlag=30, weight=0.1)  ###############################################################################  Computing the last maxlag quasi-sequence-order descriptors for      a given protein sequence.     Usage:     result = GetQuasiSequenceOrder2(protein,maxlag,weigt)     see method GetQuasiSequenceOrder for the choice of parameters.  ###############################################################################  **GetSequenceOrderCouplingNumber**(ProteinSequence, d=1)  ###############################################################################  Computing the dth-rank sequence order coupling number for a protein.     Usage:     result = GetSequenceOrderCouplingNumber(protein,d)     Input: protein is a pure protein sequence.     d is the gap between two amino acids.     Output: result is numeric value.     ###############################################################################  **GetSequenceOrderCouplingNumberTotal**(ProteinSequence, maxlag=30)  ###############################################################################  Computing the sequence order coupling numbers from 1 to maxlag     for a given protein sequence.     Usage:     result = GetSequenceOrderCouplingNumberTotal(protein, maxlag)     Input: protein is a pure protein sequence     maxlag is the maximum lag and the length of the protein should be larger     than maxlag. default is 30.     Output: result is a dict form containing all sequence order coupling numbers  ############################################################################### |

|  |  |  |
| --- | --- | --- |
| **Data** | | |
|  |  | **AALetter** = ['A', 'R', 'N', 'D', 'C', 'E', 'Q', 'G', 'H', 'I', 'L', 'K', 'M', 'F', 'P', 'S', 'T', 'W', 'Y', 'V'] |
