# Supplementary material for: Protein language model-based prediction for plant miRNA encoded peptides
Source: PeerJ Comput Sci. 2025 Mar 18;11:e2733. doi: 10.7717/peerj-cs.2733 (PMC11935769; doi:10.7717/peerj-cs.2733)
Supplement: Supplemental Information 1 [file peerj-cs-11-2733-s001.zip › pLM4PEP-master/pydpi/html/bcut.html]

Python: module bcut

|  |  |
| --- | --- |
| **bcut** | index /home/orient/pydpi/src/pydpi/drug/bcut.py |

##############################################################################  
   
The calculation of Burden eigvenvalue descriptors. You can get 64  
   
molecular decriptors. You can freely use and distribute it. If you hava    
   
any problem, you could contact with us timely!  
   
Authors: Dongsheng Cao and Yizeng Liang.  
   
Date: 2012.09.18  
   
Email: oriental-cds@163.com  
   
##############################################################################

**Modules**

|  |  |  |  |
| --- | --- | --- | --- |
| rdkit.Chem | numpy |  |  |

|  |  |  |
| --- | --- | --- |
| **Functions** | | |
|  |  | **CalculateBurdenElectronegativity**(mol)  #################################################################  Calculate Burden descriptors based on atomic electronegativity.     res-->dict type with 16 descriptors  #################################################################  **CalculateBurdenMass**(mol)  #################################################################  Calculate Burden descriptors based on atomic mass.     res--->dict type with 16 descriptors  #################################################################  **CalculateBurdenPolarizability**(mol)  #################################################################  Calculate Burden descriptors based on polarizability.     res-->dict type with 16 descriptors  #################################################################  **CalculateBurdenVDW**(mol)  #################################################################  Calculate Burden descriptors based on atomic vloumes     res-->dict type with 16 descriptors  #################################################################  **GetBurden**(mol)  #################################################################  Calculate all 64 Burden descriptors     res-->dict type  ################################################################# |

|  |  |  |
| --- | --- | --- |
| **Data** | | |
|  |  | **Version** = 1.0 |
