# Supplementary material for: Protein language model-based prediction for plant miRNA encoded peptides
Source: PeerJ Comput Sci. 2025 Mar 18;11:e2733. doi: 10.7717/peerj-cs.2733 (PMC11935769; doi:10.7717/peerj-cs.2733)
Supplement: Supplemental Information 1 [file peerj-cs-11-2733-s001.zip › pLM4PEP-master/pydpi/html/charge.html]

Python: module charge

|  |  |
| --- | --- |
| **charge** | index /home/orient/pydpi/drug/charge.py |

##############################################################################  
   
The calculation of Charge descriptors based on Gasteiger/Marseli partial   
   
charges(25). You can freely use and distribute it. If you hava  any problem,   
   
you could contact with us timely!  
   
Authors: Dongsheng Cao and Yizeng Liang.  
   
Date: 2012.09.18  
   
Email: oriental-cds@163.com  
   
##############################################################################

**Modules**

|  |  |  |  |
| --- | --- | --- | --- |
| rdkit.Chem | rdkit.Chem.rdPartialCharges | numpy |  |

|  |  |  |
| --- | --- | --- |
| **Functions** | | |
|  |  | **CalculateAllMaxNCharge**(mol)  #################################################################  Most negative charge on all atoms     -->Qmin     Usage:         result=CalculateAllMaxNCharge(mol)         Input: mol is a molecule object.         Output: result is a numeric value.  #################################################################  **CalculateAllMaxPCharge**(mol)  #################################################################  Most positive charge on ALL atoms     -->Qmax     Usage:         result=CalculateAllMaxPCharge(mol)         Input: mol is a molecule object.         Output: result is a numeric value.  #################################################################  **CalculateAllSumSquareCharge**(mol)  #################################################################  The sum of square charges on all atoms     -->Qass     Usage:         result=CalculateAllSumSquareCharge(mol)         Input: mol is a molecule object.         Output: result is a numeric value.  #################################################################  **CalculateCMaxNCharge**(mol)  #################################################################  Most negative charge on C atoms     -->QCmin     Usage:         result=CalculateCMaxNCharge(mol)         Input: mol is a molecule object.         Output: result is a numeric value.  #################################################################  **CalculateCMaxPCharge**(mol)  #################################################################  Most positive charge on C atoms     -->QCmax     Usage:         result=CalculateCMaxPCharge(mol)         Input: mol is a molecule object.         Output: result is a numeric value.  #################################################################  **CalculateCSumSquareCharge**(mol)  #################################################################  The sum of square charges on all C atoms     -->QCss     Usage:         result=CalculateCSumSquareCharge(mol)         Input: mol is a molecule object.         Output: result is a numeric value.  #################################################################  **CalculateHMaxNCharge**(mol)  #################################################################  Most negative charge on H atoms     -->QHmin     Usage:         result=CalculateHMaxNCharge(mol)         Input: mol is a molecule object.         Output: result is a numeric value.  #################################################################  **CalculateHMaxPCharge**(mol)  #################################################################  Most positive charge on H atoms     -->QHmax     Usage:         result=CalculateHMaxPCharge(mol)         Input: mol is a molecule object.         Output: result is a numeric value.  #################################################################  **CalculateHSumSquareCharge**(mol)  #################################################################  The sum of square charges on all H atoms     -->QHss     Usage:         result=CalculateHSumSquareCharge(mol)         Input: mol is a molecule object.         Output: result is a numeric value.  #################################################################  **CalculateLocalDipoleIndex**(mol)  #################################################################  Calculation of local dipole index (D)     -->LDI     Usage:         result=CalculateLocalDipoleIndex(mol)         Input: mol is a molecule object.         Output: result is a numeric value.  #################################################################  **CalculateMeanAbsoulteCharge**(mol)  #################################################################  The average absolute charge     -->Mac     Usage:         result=CalculateMeanAbsoulteCharge(mol)         Input: mol is a molecule object.         Output: result is a numeric value.  #################################################################  **CalculateMeanNCharge**(mol)  #################################################################  The average negative charge     -->Mnc     Usage:         result=CalculateMeanNCharge(mol)         Input: mol is a molecule object.         Output: result is a numeric value.  #################################################################  **CalculateMeanPCharge**(mol)  #################################################################  The average postive charge     -->Mpc     Usage:         result=CalculateMeanPCharge(mol)         Input: mol is a molecule object.         Output: result is a numeric value.  #################################################################  **CalculateNMaxNCharge**(mol)  #################################################################  Most negative charge on N atoms     -->QNmin     Usage:         result=CalculateNMaxNCharge(mol)         Input: mol is a molecule object.         Output: result is a numeric value.  #################################################################  **CalculateNMaxPCharge**(mol)  #################################################################  Most positive charge on N atoms     -->QNmax     Usage:         result=CalculateNMaxPCharge(mol)         Input: mol is a molecule object.         Output: result is a numeric value.  #################################################################  **CalculateNSumSquareCharge**(mol)  #################################################################  The sum of square charges on all N atoms     -->QNss     Usage:         result=CalculateNSumSquareCharge(mol)         Input: mol is a molecule object.         Output: result is a numeric value.  #################################################################  **CalculateOMaxNCharge**(mol)  #################################################################  Most negative charge on O atoms     -->QOmin     Usage:         result=CalculateOMaxNCharge(mol)         Input: mol is a molecule object.         Output: result is a numeric value.  #################################################################  **CalculateOMaxPCharge**(mol)  #################################################################  Most positive charge on O atoms     -->QOmax     Usage:         result=CalculateOMaxPCharge(mol)         Input: mol is a molecule object.         Output: result is a numeric value.  #################################################################  **CalculateOSumSquareCharge**(mol)  #################################################################  The sum of square charges on all O atoms     -->QOss     Usage:         result=CalculateOSumSquareCharge(mol)         Input: mol is a molecule object.         Output: result is a numeric value.  #################################################################  **CalculateRelativeNCharge**(mol)  #################################################################  The partial charge of the most negative atom divided     by the total negative charge.     -->Rnc     Usage:         result=CalculateRelativeNCharge(mol)         Input: mol is a molecule object.         Output: result is a numeric value.  #################################################################  **CalculateRelativePCharge**(mol)  #################################################################  The partial charge of the most positive atom divided by     the total positive charge.     -->Rpc     Usage:         result=CalculateRelativePCharge(mol)         Input: mol is a molecule object.         Output: result is a numeric value.  #################################################################  **CalculateSubmolPolarityPara**(mol)  #################################################################  Calculation of submolecular polarity parameter(SPP)     -->SPP     Usage:         result=CalculateSubmolPolarityPara(mol)         Input: mol is a molecule object.         Output: result is a numeric value.  #################################################################  **CalculateTotalAbsoulteCharge**(mol)  #################################################################  The total absolute charge     -->Tac     Usage:         result=CalculateTotalAbsoulteCharge(mol)         Input: mol is a molecule object.         Output: result is a numeric value.  #################################################################  **CalculateTotalNCharge**(mol)  #################################################################  The total negative charge     -->Tnc     Usage:         result=CalculateTotalNCharge(mol)         Input: mol is a molecule object.         Output: result is a numeric value.  #################################################################  **CalculateTotalPCharge**(mol)  #################################################################  The total postive charge     -->Tpc     Usage:         result=CalculateTotalPCharge(mol)         Input: mol is a molecule object.         Output: result is a numeric value.  #################################################################  **GetCharge**(mol)  #################################################################  Get the dictionary of constitutional descriptors for given moelcule mol     Usage:         result=GetCharge(mol)         Input: mol is a molecule object.         Output: result is a dict form containing all charge descriptors.  ################################################################# |

|  |  |  |
| --- | --- | --- |
| **Data** | | |
|  |  | **Version** = 1.0  **iter\_step** = 12 |
