# Supplementary material for: Protein language model-based prediction for plant miRNA encoded peptides
Source: PeerJ Comput Sci. 2025 Mar 18;11:e2733. doi: 10.7717/peerj-cs.2733 (PMC11935769; doi:10.7717/peerj-cs.2733)
Supplement: Supplemental Information 1 [file peerj-cs-11-2733-s001.zip › pLM4PEP-master/pydpi/html/connectivity.html]

Python: module connectivity

|  |  |
| --- | --- |
| **connectivity** | index /home/orient/pydpi/drug/connectivity.py |

##############################################################################  
The calculation of molecular connectivity indices based on its topological  
   
structure. You can get 44 molecular connectivity descriptors. You can freely  
   
use and distribute it. If you hava  any problem, you could contact with us timely!  
   
Authors: Dongsheng Cao and Yizeng Liang.  
   
Date: 2012.09.18  
   
Email: oriental-cds@163.com  
##############################################################################

**Modules**

|  |  |  |  |
| --- | --- | --- | --- |
| rdkit.Chem | numpy | rdkit.Chem.rdchem |  |

|  |  |  |
| --- | --- | --- |
| **Functions** | | |
|  |  | **CalculateChi0**(mol)  #################################################################  Calculation of molecular connectivity chi index for path order 0     ---->Chi0     Usage:            result=CalculateChi0(mol)            Input: mol is a molecule object.            Output: result is a numeric value  #################################################################  **CalculateChi1**(mol)  #################################################################  Calculation of molecular connectivity chi index for path order 1      (i.e.,Radich)     ---->Chi1     Usage:            result=CalculateChi1(mol)            Input: mol is a molecule object.            Output: result is a numeric value  #################################################################  **CalculateChi10p**(mol)  #################################################################  Calculation of molecular connectivity chi index for path order 10     ---->Chi10     Usage:            result=CalculateChi10p(mol)            Input: mol is a molecule object.            Output: result is a numeric value  #################################################################  **CalculateChi2**(mol)  #################################################################  Calculation of molecular connectivity chi index for path order 2     ---->Chi2     Usage:            result=CalculateChi2(mol)            Input: mol is a molecule object.            Output: result is a numeric value  #################################################################  **CalculateChi3c**(mol)  #################################################################  Calculation of molecular connectivity chi index for cluster     ---->Chi3c     Usage:            result=CalculateChi3c(mol)            Input: mol is a molecule object.            Output: result is a numeric value  #################################################################  **CalculateChi3ch**(mol)  #################################################################  Calculation of molecular connectivity chi index for cycles of 3     ---->Chi3ch     Usage:            result=CalculateChi3ch(mol)            Input: mol is a molecule object.            Output: result is a numeric value  #################################################################  **CalculateChi3p**(mol)  #################################################################  Calculation of molecular connectivity chi index for path order 3     ---->Chi3     Usage:            result=CalculateChi3p(mol)            Input: mol is a molecule object.            Output: result is a numeric value  #################################################################  **CalculateChi4c**(mol)  #################################################################  Calculation of molecular connectivity chi index for cluster     ---->Chi4c     Usage:            result=CalculateChi4c(mol)            Input: mol is a molecule object.            Output: result is a numeric value  #################################################################  **CalculateChi4ch**(mol)  #################################################################  Calculation of molecular connectivity chi index for cycles of 4     ---->Chi4ch     Usage:            result=CalculateChi4ch(mol)            Input: mol is a molecule object.            Output: result is a numeric value  #################################################################  **CalculateChi4p**(mol)  #################################################################  Calculation of molecular connectivity chi index for path order 4     ---->Chi4     Usage:            result=CalculateChi4p(mol)            Input: mol is a molecule object.            Output: result is a numeric value  #################################################################  **CalculateChi4pc**(mol)  #################################################################  Calculation of molecular connectivity chi index for path/cluster     ---->Chi4pc     Usage:            result=CalculateChi4pc(mol)            Input: mol is a molecule object.            Output: result is a numeric value  #################################################################  **CalculateChi5ch**(mol)  #################################################################  Calculation of molecular connectivity chi index for cycles of 5     ---->Chi5ch     Usage:            result=CalculateChi5ch(mol)            Input: mol is a molecule object.            Output: result is a numeric value  #################################################################  **CalculateChi5p**(mol)  #################################################################  Calculation of molecular connectivity chi index for path order 5     ---->Chi5     Usage:            result=CalculateChi5p(mol)            Input: mol is a molecule object.            Output: result is a numeric value  #################################################################  **CalculateChi6ch**(mol)  #################################################################  Calculation of molecular connectivity chi index for cycles of 6     ---->Chi6ch     Usage:            result=CalculateChi6ch(mol)            Input: mol is a molecule object.            Output: result is a numeric value  #################################################################  **CalculateChi6p**(mol)  #################################################################  Calculation of molecular connectivity chi index for path order 6     ---->Chi6     Usage:            result=CalculateChi6p(mol)            Input: mol is a molecule object.            Output: result is a numeric value  #################################################################  **CalculateChi7p**(mol)  #################################################################  Calculation of molecular connectivity chi index for path order 7     ---->Chi7     Usage:            result=CalculateChi7p(mol)            Input: mol is a molecule object.            Output: result is a numeric value  #################################################################  **CalculateChi8p**(mol)  #################################################################  Calculation of molecular connectivity chi index for path order 8     ---->Chi8     Usage:            result=CalculateChi8p(mol)            Input: mol is a molecule object.            Output: result is a numeric value  #################################################################  **CalculateChi9p**(mol)  #################################################################  Calculation of molecular connectivity chi index for path order 9     ---->Chi9     Usage:            result=CalculateChi9p(mol)            Input: mol is a molecule object.            Output: result is a numeric value  #################################################################  **CalculateChiv0**(mol)  #################################################################  Calculation of valence molecular connectivity chi index for     path order 0     ---->Chiv0     Usage:            result=CalculateChiv0(mol)            Input: mol is a molecule object.            Output: result is a numeric value  #################################################################  **CalculateChiv1**(mol)  #################################################################  Calculation of valence molecular connectivity chi index for     path order 1     ---->Chiv1     Usage:            result=CalculateChiv1(mol)            Input: mol is a molecule object.            Output: result is a numeric value  #################################################################  **CalculateChiv10p**(mol)  #################################################################  Calculation of valence molecular connectivity chi index for      path order 10     ---->Chiv10     Usage:            result=CalculateChiv10p(mol)            Input: mol is a molecule object.            Output: result is a numeric value  #################################################################  **CalculateChiv2**(mol)  #################################################################  Calculation of valence molecular connectivity chi index for      path order 2     ---->Chiv2     Usage:            result=CalculateChiv2(mol)            Input: mol is a molecule object.            Output: result is a numeric value  #################################################################  **CalculateChiv3c**(mol)  #################################################################  Calculation of valence molecular connectivity chi index for cluster     ---->Chiv3c     Usage:            result=CalculateChiv3c(mol)            Input: mol is a molecule object.            Output: result is a numeric value  #################################################################  **CalculateChiv3ch**(mol)  #################################################################  Calculation of valence molecular connectivity chi index      for cycles of 3     ---->Chiv3ch     Usage:            result=CalculateChiv3ch(mol)            Input: mol is a molecule object.            Output: result is a numeric value  #################################################################  **CalculateChiv3p**(mol)  #################################################################  Calculation of valence molecular connectivity chi index for      path order 3     ---->Chiv3     Usage:            result=CalculateChiv3p(mol)            Input: mol is a molecule object.            Output: result is a numeric value  #################################################################  **CalculateChiv4c**(mol)  #################################################################  Calculation of valence molecular connectivity chi index for cluster     ---->Chiv4c     Usage:            result=CalculateChiv4c(mol)            Input: mol is a molecule object.            Output: result is a numeric value  #################################################################  **CalculateChiv4ch**(mol)  #################################################################  Calculation of valence molecular connectivity chi index for      cycles of 4     ---->Chiv4ch     Usage:            result=CalculateChiv4ch(mol)            Input: mol is a molecule object.            Output: result is a numeric value  #################################################################  **CalculateChiv4p**(mol)  #################################################################  Calculation of valence molecular connectivity chi index for     path order 4     ---->Chiv4     Usage:            result=CalculateChiv4p(mol)            Input: mol is a molecule object.            Output: result is a numeric value  #################################################################  **CalculateChiv4pc**(mol)  #################################################################  Calculation of valence molecular connectivity chi index for      path/cluster     ---->Chiv4pc     Usage:            result=CalculateChiv4pc(mol)            Input: mol is a molecule object.            Output: result is a numeric value  #################################################################  **CalculateChiv5ch**(mol)  #################################################################  Calculation of valence molecular connectivity chi index for      cycles of 5     ---->Chiv5ch     Usage:            result=CalculateChiv5ch(mol)            Input: mol is a molecule object.            Output: result is a numeric value  #################################################################  **CalculateChiv5p**(mol)  #################################################################  Calculation of valence molecular connectivity chi index for     path order 5     ---->Chiv5     Usage:            result=CalculateChiv5p(mol)            Input: mol is a molecule object.            Output: result is a numeric value  #################################################################  **CalculateChiv6ch**(mol)  #################################################################  Calculation of valence molecular connectivity chi index for     cycles of 6     ---->Chiv6ch     Usage:            result=CalculateChiv6ch(mol)            Input: mol is a molecule object.            Output: result is a numeric value  #################################################################  **CalculateChiv6p**(mol)  #################################################################  Calculation of valence molecular connectivity chi index for     path order 6     ---->Chiv6     Usage:            result=CalculateChiv6p(mol)            Input: mol is a molecule object.            Output: result is a numeric value  #################################################################  **CalculateChiv7p**(mol)  #################################################################  Calculation of valence molecular connectivity chi index for     path order 7     ---->Chiv7     Usage:            result=CalculateChiv7p(mol)            Input: mol is a molecule object.            Output: result is a numeric value  #################################################################  **CalculateChiv8p**(mol)  #################################################################  Calculation of valence molecular connectivity chi index for      path order 8     ---->Chiv8     Usage:            result=CalculateChiv8p(mol)            Input: mol is a molecule object.            Output: result is a numeric value  #################################################################  **CalculateChiv9p**(mol)  #################################################################  Calculation of valence molecular connectivity chi index for      path order 9     ---->Chiv9     Usage:            result=CalculateChiv9p(mol)            Input: mol is a molecule object.            Output: result is a numeric value  #################################################################  **CalculateDeltaChi0**(mol)  #################################################################  Calculation of the difference between chi0v and chi0     ---->dchi0     Usage:            result=CalculateDeltaChi0(mol)            Input: mol is a molecule object.            Output: result is a numeric value  #################################################################  **CalculateDeltaChi1**(mol)  #################################################################  Calculation of the difference between chi1v and chi1     ---->dchi1     Usage:            result=CalculateDeltaChi1(mol)            Input: mol is a molecule object.            Output: result is a numeric value  #################################################################  **CalculateDeltaChi2**(mol)  #################################################################  Calculation of the difference between chi2v and chi2     ---->dchi2     Usage:            result=CalculateDeltaChi2(mol)            Input: mol is a molecule object.            Output: result is a numeric value  #################################################################  **CalculateDeltaChi3**(mol)  #################################################################  Calculation of the difference between chi3v and chi3     ---->dchi3     Usage:            result=CalculateDeltaChi3(mol)            Input: mol is a molecule object.            Output: result is a numeric value  #################################################################  **CalculateDeltaChi3c4pc**(mol)  #################################################################  Calculation of the difference between chi3c and chi4pc     ---->knotp     Usage:            result=CalculateDeltaChi3c4pc(mol)            Input: mol is a molecule object.            Output: result is a numeric value  #################################################################  **CalculateDeltaChi4**(mol)  #################################################################  Calculation of the difference between chi4v and chi4     ---->dchi4     Usage:            result=CalculateDeltaChi4(mol)            Input: mol is a molecule object.            Output: result is a numeric value  #################################################################  **CalculateDeltaChiv3c4pc**(mol)  #################################################################  Calculation of the difference between chiv3c and chiv4pc     ---->knotpv     Usage:            result=CalculateDeltaChiv3c4pc(mol)            Input: mol is a molecule object.            Output: result is a numeric value  #################################################################  **CalculateMeanRandic**(mol)  #################################################################  Calculation of mean chi1 (Randic) connectivity index.     ---->mchi1     Usage:            result=CalculateMeanRandic(mol)            Input: mol is a molecule object.            Output: result is a numeric value  #################################################################  **GetConnectivity**(mol)  #################################################################  Get the dictionary of connectivity descriptors for given moelcule mol     Usage:            result=GetConnectivity(mol)            Input: mol is a molecule object.            Output: result is a dict form containing all connectivity indices  ################################################################# |

|  |  |  |
| --- | --- | --- |
| **Data** | | |
|  |  | **Version** = 1.0  **periodicTable** = <rdkit.Chem.rdchem.PeriodicTable object> |
