# Supplementary material for: Protein language model-based prediction for plant miRNA encoded peptides
Source: PeerJ Comput Sci. 2025 Mar 18;11:e2733. doi: 10.7717/peerj-cs.2733 (PMC11935769; doi:10.7717/peerj-cs.2733)
Supplement: Supplemental Information 1 [file peerj-cs-11-2733-s001.zip › pLM4PEP-master/pydpi/html/constitution.html]

Python: module constitution

|  |  |
| --- | --- |
| **constitution** | index /home/orient/pydpi/drug/constitution.py |

##############################################################################  
The calculation of molecular constitutional indices based on its topological  
   
structure. You can get 30 molecular connectivity descriptors. You can freely  
   
use and distribute it. If you hava  any problem, you could contact with us timely!  
   
Authors: Dongsheng Cao and Yizeng Liang.  
   
Date: 2012.09.18  
   
Email: oriental-cds@163.com  
##############################################################################

**Modules**

|  |  |  |  |
| --- | --- | --- | --- |
| rdkit.Chem | rdkit.Chem.Lipinski |  |  |

|  |  |  |
| --- | --- | --- |
| **Functions** | | |
|  |  | **CalculateAllAtomNumber**(mol)  #################################################################  Calculation of all atom counts in a molecule     ---->nta     Usage:            result=CalculateAllAtomNumber(mol)            Input: mol is a molecule object.            Output: result is a numeric value.  #################################################################  **CalculateAromaticBondNumber**(mol)  #################################################################  Calculation of aromatic bond counts in a molecule     ---->naro     Usage:            result=CalculateAromaticBondNumber(mol)            Input: mol is a molecule object.            Output: result is a numeric value.  #################################################################  **CalculateAverageMolWeight**(mol)  #################################################################  Calcualtion of average molecular weight     Note that not including H     ---->AWeight     Usage:            result=CalculateAverageMolWeight(mol)            Input: mol is a molecule object.            Output: result is a numeric value.  #################################################################  **CalculateBromineNumber**(mol)  #################################################################  Calculation of Bromine counts in a molecule     ---->ncobr     Usage:            result=CalculateBromineNumber(mol)            Input: mol is a molecule object.            Output: result is a numeric value.  #################################################################  **CalculateCarbonNumber**(mol)  #################################################################  Calculation of Carbon number in a molecule     ---->ncarb     Usage:            result=CalculateCarbonNumber(mol)            Input: mol is a molecule object.            Output: result is a numeric value.  #################################################################  **CalculateChlorinNumber**(mol)  #################################################################  Calculation of Chlorin counts in a molecule     ---->ncocl     Usage:            result=CalculateChlorinNumber(mol)            Input: mol is a molecule object.            Output: result is a numeric value.  #################################################################  **CalculateDoubleBondNumber**(mol)  #################################################################  Calculation of double bond counts in a molecule     ---->ndb     Usage:            result=CalculateDoubleBondNumber(mol)            Input: mol is a molecule object.            Output: result is a numeric value.  #################################################################  **CalculateFluorinNumber**(mol)  #################################################################  Calculation of Fluorin counts in a molecule     ---->ncof     Usage:            result=CalculateFluorinNumber(mol)            Input: mol is a molecule object.            Output: result is a numeric value.  #################################################################  **CalculateHacceptorNumber**(mol)  #################################################################  Calculation of Hydrogen bond acceptor counts in a molecule     ---->naccr     Usage:            result=CalculateHacceptorNumber(mol)            Input: mol is a molecule object.            Output: result is a numeric value.  #################################################################  **CalculateHalogenNumber**(mol)  #################################################################  Calculation of Halogen counts in a molecule     ---->nhal     Usage:            result=CalculateHalogenNumber(mol)            Input: mol is a molecule object.            Output: result is a numeric value.  #################################################################  **CalculateHdonorNumber**(mol)  #################################################################  Calculation of Hydrongen bond donor counts in a molecule     ---->ndonr     Usage:            result=CalculateHdonorNumber(mol)            Input: mol is a molecule object.            Output: result is a numeric value.  #################################################################  **CalculateHeavyAtomNumber**(mol)  #################################################################  Calculation of Heavy atom counts in a molecule     ---->nhev     Usage:            result=CalculateHeavyAtomNumber(mol)            Input: mol is a molecule object.            Output: result is a numeric value.  #################################################################  **CalculateHeteroNumber**(mol)  #################################################################  Calculation of Hetero counts in a molecule     ---->nhet     Usage:            result=CalculateHeteroNumber(mol)            Input: mol is a molecule object.            Output: result is a numeric value.  #################################################################  **CalculateHydrogenNumber**(mol)  #################################################################  Calculation of Number of Hydrogen in a molecule     ---->nhyd     Usage:            result=CalculateHydrogenNumber(mol)            Input: mol is a molecule object.            Output: result is a numeric value.  #################################################################  **CalculateIodineNumber**(mol)  #################################################################  Calculation of Iodine counts in a molecule     ---->ncoi     Usage:            result=CalculateIodineNumber(mol)            Input: mol is a molecule object.            Output: result is a numeric value.  #################################################################  **CalculateMolWeight**(mol)  #################################################################  Calculation of molecular weight     Note that not including H     ---->Weight       Usage:            result=CalculateMolWeight(mol)            Input: mol is a molecule object.            Output: result is a numeric value.        #################################################################  **CalculateNitrogenNumber**(mol)  #################################################################  Calculation of Nitrogen counts in a molecule     ---->nnitro     Usage:            result=CalculateNitrogenNumber(mol)            Input: mol is a molecule object.            Output: result is a numeric value.  #################################################################  **CalculateOxygenNumber**(mol)  #################################################################  Calculation of Oxygen counts in a molecule     ---->noxy     Usage:            result=CalculateOxygenNumber(mol)            Input: mol is a molecule object.            Output: result is a numeric value.  #################################################################  **CalculatePath1**(mol)  #################################################################  Calculation of the counts of path length 1 for a molecule  #################################################################  **CalculatePath2**(mol)  #################################################################  Calculation of the counts of path length 2 for a molecule  #################################################################  **CalculatePath3**(mol)  #################################################################  Calculation of the counts of path length 3 for a molecule  #################################################################  **CalculatePath4**(mol)  #################################################################  Calculation of the counts of path length 4 for a molecule  #################################################################  **CalculatePath5**(mol)  #################################################################  Calculation of the counts of path length 5 for a molecule  #################################################################  **CalculatePath6**(mol)  #################################################################  Calculation of the counts of path length 6 for a molecule  #################################################################  **CalculatePhosphorNumber**(mol)  #################################################################  Calcualtion of Phosphor number in a molecule     ---->nphos     Usage:            result=CalculatePhosphorNumber(mol)            Input: mol is a molecule object.            Output: result is a numeric value.  #################################################################  **CalculateRingNumber**(mol)  #################################################################  Calculation of ring counts in a molecule     ---->nring     Usage:            result=CalculateRingNumber(mol)            Input: mol is a molecule object.            Output: result is a numeric value.  #################################################################  **CalculateRotationBondNumber**(mol)  #################################################################  Calculation of rotation bonds counts in a molecule     ---->nrot     Note that this is the same as calculation of single bond     counts in a molecule.     Usage:            result=CalculateRotationBondNumber(mol)            Input: mol is a molecule object.            Output: result is a numeric value.  #################################################################  **CalculateSingleBondNumber**(mol)  #################################################################  Calculation of single bond counts in a molecule     ---->nsb     Usage:            result=CalculateSingleBondNumber(mol)            Input: mol is a molecule object.            Output: result is a numeric value.  #################################################################  **CalculateSulfurNumber**(mol)  #################################################################  Calculation of Sulfur counts in a molecule     ---->nsulph     Usage:            result=CalculateSulfurNumber(mol)            Input: mol is a molecule object.            Output: result is a numeric value.  #################################################################  **CalculateTripleBondNumber**(mol)  #################################################################  Calculation of triple bond counts in a molecule     ---->ntb     Usage:            result=CalculateTripleBondNumber(mol)            Input: mol is a molecule object.            Output: result is a numeric value.  #################################################################  **GetConstitutional**(mol)  #################################################################  Get the dictionary of constitutional descriptors for given moelcule mol     Usage:            result=GetConstitutional(mol)            Input: mol is a molecule object.            Output: result is a dict form containing all constitutional values.  ################################################################# |

|  |  |  |
| --- | --- | --- |
| **Data** | | |
|  |  | **Version** = 1.0 |
