# Supplementary material for: Protein language model-based prediction for plant miRNA encoded peptides
Source: PeerJ Comput Sci. 2025 Mar 18;11:e2733. doi: 10.7717/peerj-cs.2733 (PMC11935769; doi:10.7717/peerj-cs.2733)
Supplement: Supplemental Information 1 [file peerj-cs-11-2733-s001.zip › pLM4PEP-master/pydpi/html/estate.html]

Python: module estate

|  |  |
| --- | --- |
| **estate** | index /home/orient/pydpi/drug/estate.py |

##############################################################################  
This module is to compute the estate fingerprints and values based on Kier   
   
and Hall's paper. If you have any question please contact me via email.  
   
My email adress is : orientalcds@gmail.com  
   
Created on Tue May 24 14:32:52 2011  
   
@author: Dongsheng Cao  
##############################################################################

**Modules**

|  |  |  |  |
| --- | --- | --- | --- |
| AtomTypes | rdkit.Chem | rdkit.Chem.EState.Fingerprinter | numpy |

|  |  |  |
| --- | --- | --- |
| **Functions** | | |
|  |  | **CalculateEstateFingerprint**(mol)  #################################################################  The Calculation of EState Fingerprints.     It is the number of times each possible atom type is hit.     Usage:            result=CalculateEstateFingerprint(mol)            Input: mol is a molecule object.            Output: result is a dict form containing 79 estate fragments.  #################################################################  **CalculateEstateValue**(mol)  #################################################################  The Calculate of EState Values.     It is the sum of the Estate indices for atoms of each type.     Usage:            result=CalculateEstateValue(mol)            Input: mol is a molecule object.            Output: result is a dict form containing 79 estate values.  #################################################################  **CalculateMaxAtomTypeEState**(mol)  #################################################################  Calculation of maximum of E-State value of specified atom type     res---->dict type     Usage:            result=CalculateMaxAtomTypeEState(mol)            Input: mol is a molecule object.            Output: result is a dict form containing 79 max estate values.  #################################################################  **CalculateMinAtomTypeEState**(mol)  #################################################################  Calculation of minimum of E-State value of specified atom type     res---->dict type     Usage:            result=CalculateMinAtomTypeEState(mol)            Input: mol is a molecule object.            Output: result is a dict form containing 79 min estate values.  #################################################################  **GetEstate**(mol)  #################################################################  Obtain all descriptors related to Estate.     Usage:            result=GetEstate(mol)            Input: mol is a molecule object.            Output: result is a dict form containing all estate values.  ################################################################# |

|  |  |  |
| --- | --- | --- |
| **Data** | | |
|  |  | **Version** = 1.0 |
