# Supplementary material for: Protein language model-based prediction for plant miRNA encoded peptides
Source: PeerJ Comput Sci. 2025 Mar 18;11:e2733. doi: 10.7717/peerj-cs.2733 (PMC11935769; doi:10.7717/peerj-cs.2733)
Supplement: Supplemental Information 1 [file peerj-cs-11-2733-s001.zip › pLM4PEP-master/pydpi/html/fingerprint.html]

Python: module fingerprint

|  |  |
| --- | --- |
| **fingerprint** | index /home/orient/pydpi/src/pydpi/drug/fingerprint.py |

##############################################################################  
This module is to compute the various fingerprints  based on the provided   
   
fingerprint system. If you have any question please contact me via email.  
   
My email adress is : orientalcds@gmail.com  
   
2012.09.25  
   
@author: Dongsheng Cao  
##############################################################################

**Modules**

|  |  |  |  |
| --- | --- | --- | --- |
| rdkit.Chem.AllChem  rdkit.Chem | rdkit.DataStructs  rdkit.Chem.Fingerprints.FingerprintMols | rdkit.Chem.MACCSkeys  rdkit.Chem.AtomPairs.Pairs | rdkit.Chem.AtomPairs.Torsions |

|  |  |  |
| --- | --- | --- |
| **Functions** | | |
|  |  | **CalculateAtomPairsFingerprint**(mol)  #################################################################  Calculate atom pairs fingerprints     Usage:            result=CalculateAtomPairsFingerprint(mol)            Input: mol is a molecule object.            Output: result is a tuple form. The first is the number of             fingerprints. The second is a dict form whose keys are the             position which this molecule has some substructure. The third            is the DataStructs which is used for calculating the similarity.  #################################################################  **CalculateDaylightFingerprint**(mol)  #################################################################  Calculate Daylight-like fingerprint or topological fingerprint     (2048 bits).     Usage:            result=CalculateDaylightFingerprint(mol)            Input: mol is a molecule object.            Output: result is a tuple form. The first is the number of             fingerprints. The second is a dict form whose keys are the             position which this molecule has some substructure. The third            is the DataStructs which is used for calculating the similarity.  #################################################################  **CalculateEstateFingerprint**(mol)  #################################################################  Calculate E-state fingerprints (79 bits).     Usage:            result=CalculateEstateFingerprint(mol)            Input: mol is a molecule object.            Output: result is a tuple form. The first is the number of             fingerprints. The second is a dict form whose keys are the             position which this molecule has some substructure. The third            is the DataStructs which is used for calculating the similarity.  #################################################################  **CalculateFP4Fingerprint**(mol)  #################################################################  Calculate FP4 fingerprints (307 bits).     Usage:            result=CalculateFP4Fingerprint(mol)            Input: mol is a molecule object.            Output: result is a tuple form. The first is the number of             fingerprints. The second is a dict form whose keys are the             position which this molecule has some substructure. The third            is the DataStructs which is used for calculating the similarity.  #################################################################  **CalculateMACCSFingerprint**(mol)  #################################################################  Calculate MACCS keys (166 bits).     Usage:            result=CalculateMACCSFingerprint(mol)            Input: mol is a molecule object.            Output: result is a tuple form. The first is the number of             fingerprints. The second is a dict form whose keys are the             position which this molecule has some substructure. The third            is the DataStructs which is used for calculating the similarity.  #################################################################  **CalculateMorganFingerprint**(mol, radius=2)  #################################################################  Calculate Morgan     Usage:            result=CalculateMorganFingerprint(mol)            Input: mol is a molecule object.            radius is a radius.            Output: result is a tuple form. The first is the number of             fingerprints. The second is a dict form whose keys are the             position which this molecule has some substructure. The third            is the DataStructs which is used for calculating the similarity.  #################################################################  **CalculateSimilarity**(fp1, fp2, similarity='Tanimoto')  #################################################################  Calculate similarity between two molecules.     Usage:            result=CalculateSimilarity(fp1,fp2)            Input: fp1 and fp2 are two DataStructs.            Output: result is a similarity value.  #################################################################  **CalculateTopologicalTorsionFingerprint**(mol)  #################################################################  Calculate Topological Torsion Fingerprints     Usage:            result=CalculateTopologicalTorsionFingerprint(mol)            Input: mol is a molecule object.            Output: result is a tuple form. The first is the number of             fingerprints. The second is a dict form whose keys are the             position which this molecule has some substructure. The third            is the DataStructs which is used for calculating the similarity.  ################################################################# |

|  |  |  |
| --- | --- | --- |
| **Data** | | |
|  |  | **Version** = 1.0  **similaritymeasure** = ['Tanimoto', 'Dice', 'Cosine', 'Sokal', 'Russel', 'Kulczynski', 'McConnaughey', 'Asymmetric', 'BraunBlanquet'] |
