# Supplementary material for: Protein language model-based prediction for plant miRNA encoded peptides
Source: PeerJ Comput Sci. 2025 Mar 18;11:e2733. doi: 10.7717/peerj-cs.2733 (PMC11935769; doi:10.7717/peerj-cs.2733)
Supplement: Supplemental Information 1 [file peerj-cs-11-2733-s001.zip › pLM4PEP-master/pydpi/html/geary.html]

Python: module geary

|  |  |
| --- | --- |
| **geary** | index /home/orient/pydpi/drug/geary.py |

##############################################################################  
The calculation of Geary autocorrelation indices based on its topological  
   
structure. You can get 32 molecular autocorrelation descriptors. You can   
   
freely use and distribute it. If you hava  any problem, you could contact   
   
with us timely!  
   
Authors: Dongsheng Cao and Yizeng Liang.  
   
Date: 2012.09.18  
   
Email: oriental-cds@163.com  
##############################################################################

**Modules**

|  |  |  |  |
| --- | --- | --- | --- |
| rdkit.Chem | numpy |  |  |

|  |  |  |
| --- | --- | --- |
| **Functions** | | |
|  |  | **CalculateGearyAutoElectronegativity**(mol)  #################################################################  Calculation of Geary autocorrelation descriptors based on      carbon-scaled atomic Sanderson electronegativity.     Usage:     res=CalculateGearyAutoElectronegativity(mol)     Input: mol is a molecule object.     Output: res is a dict form containing eight geary autocorrealtion     descriptors.  #################################################################  **CalculateGearyAutoMass**(mol)  #################################################################  Calculation of Geary autocorrelation descriptors based on      carbon-scaled atomic mass.     Usage:     res=CalculateMoranAutoMass(mol)     Input: mol is a molecule object.     Output: res is a dict form containing eight geary autocorrealtion     descriptors.  #################################################################  **CalculateGearyAutoPolarizability**(mol)  #################################################################  Calculation of Geary autocorrelation descriptors based on      carbon-scaled atomic polarizability.     Usage:     res=CalculateGearyAutoPolarizability(mol)     Input: mol is a molecule object.     Output: res is a dict form containing eight geary autocorrealtion     descriptors.  #################################################################  **CalculateGearyAutoVolume**(mol)  #################################################################  Calculation of Geary autocorrelation descriptors based on      carbon-scaled atomic van der Waals volume.     Usage:     res=CalculateGearyAutoVolume(mol)     Input: mol is a molecule object.     Output: res is a dict form containing eight geary autocorrealtion     descriptors.  #################################################################  **GetGearyAuto**(mol)  #################################################################  Calcualate all Geary autocorrelation descriptors.     (carbon-scaled atomic mass, carbon-scaled atomic van der Waals volume,     carbon-scaled atomic Sanderson electronegativity,     carbon-scaled atomic polarizability)     Usage:     res=GetGearyAuto(mol)     Input: mol is a molecule object.     Output: res is a dict form containing all geary autocorrealtion     descriptors.  ################################################################# |

|  |  |  |
| --- | --- | --- |
| **Data** | | |
|  |  | **Version** = 1.0 |
