# Supplementary material for: Protein language model-based prediction for plant miRNA encoded peptides
Source: PeerJ Comput Sci. 2025 Mar 18;11:e2733. doi: 10.7717/peerj-cs.2733 (PMC11935769; doi:10.7717/peerj-cs.2733)
Supplement: Supplemental Information 1 [file peerj-cs-11-2733-s001.zip › pLM4PEP-master/pydpi/html/getpdb.html]

Python: module getpdb

|  |  |
| --- | --- |
| **getpdb** | index /home/orient/pydpi/src/pydpi/protein/getpdb.py |

Created on Sat Jul 13 11:18:26 2013  
   
This module is used for downloading the PDB file from RCSB PDB web and   
   
extract its amino acid sequence.  
   
This is written by Dongsheng Cao  
   
@author: Dongsheng Cao

**Modules**

|  |  |  |  |
| --- | --- | --- | --- |
| ftplib | gzip | os |  |

|  |  |  |
| --- | --- | --- |
| **Functions** | | |
|  |  | **GetPDB**(pdbidlist=[])  Download the PDB file from PDB FTP server by providing a list of pdb id.  **GetSeqFromPDB**(pdbfile='')  Get the amino acids sequences from pdb file.  **pdbDownload**(file\_list, hostname='ftp.wwpdb.org', directory='/pub/pdb/data/structures/all/pdb/', prefix='pdb', suffix='.ent.gz')  Download all pdb files in file\_list and unzip them.  **pdbSeq**(pdb, use\_atoms=False)  Parse the SEQRES entries in a pdb file.  If this fails, use the ATOM  entries.  Return dictionary of sequences keyed to chain and type of  sequence used.  **unZip**(some\_file, some\_output)  Unzip some\_file using the gzip library and write to some\_output. |

|  |  |  |
| --- | --- | --- |
| **Data** | | |
|  |  | **DIRECTORY** = '/pub/pdb/data/structures/all/pdb/'  **HOSTNAME** = 'ftp.wwpdb.org'  **PREFIX** = 'pdb'  **SUFFIX** = '.ent.gz' |
