# Supplementary material for: Protein language model-based prediction for plant miRNA encoded peptides
Source: PeerJ Comput Sci. 2025 Mar 18;11:e2733. doi: 10.7717/peerj-cs.2733 (PMC11935769; doi:10.7717/peerj-cs.2733)
Supplement: Supplemental Information 1 [file peerj-cs-11-2733-s001.zip › pLM4PEP-master/pydpi/html/kappa.html]

Python: module kappa

|  |  |
| --- | --- |
| **kappa** | index /home/orient/pydpi/drug/kappa.py |

##############################################################################  
The calculation of Kier and Hall's kappa indices based on its topological  
   
structure. You can get 7 molecular kappa descriptors. You can   
   
freely use and distribute it. If you hava  any problem, you could contact   
   
with us timely!  
   
Authors: Dongsheng Cao and Yizeng Liang.  
   
Date: 2012.09.18  
   
Email: oriental-cds@163.com  
##############################################################################

**Modules**

|  |  |  |  |
| --- | --- | --- | --- |
| rdkit.Chem | rdkit.Chem.PeriodicTable | rdkit.Chem.rdchem |  |

|  |  |  |
| --- | --- | --- |
| **Functions** | | |
|  |  | **CalculateFlexibility**(mol)  #################################################################  Calculation of Kier molecular flexibility index     ---->phi     Usage:            result=CalculateFlexibility(mol)            Input: mol is a molecule object.            Output: result is a numeric value.  #################################################################  **CalculateKappa1**(mol)  #################################################################  Calculation of molecular shape index for one bonded fragment     ---->kappa1     Usage:            result=CalculateKappa1(mol)            Input: mol is a molecule object.            Output: result is a numeric value.  #################################################################  **CalculateKappa2**(mol)  #################################################################  Calculation of molecular shape index for two bonded fragment     ---->kappa2     Usage:            result=CalculateKappa2(mol)            Input: mol is a molecule object.            Output: result is a numeric value.  #################################################################  **CalculateKappa3**(mol)  #################################################################  Calculation of molecular shape index for three bonded fragment     ---->kappa3     Usage:            result=CalculateKappa3(mol)            Input: mol is a molecule object.            Output: result is a numeric value.  #################################################################  **CalculateKappaAlapha1**(mol)  #################################################################  Calculation of molecular shape index for one bonded fragment      with Alapha     ---->kappam1     Usage:            result=CalculateKappaAlapha1(mol)            Input: mol is a molecule object.            Output: result is a numeric value.  #################################################################  **CalculateKappaAlapha2**(mol)  #################################################################  Calculation of molecular shape index for two bonded fragment      with Alapha     ---->kappam2     Usage:            result=CalculateKappaAlapha2(mol)            Input: mol is a molecule object.            Output: result is a numeric value.  #################################################################  **CalculateKappaAlapha3**(mol)  #################################################################  Calculation of molecular shape index for three bonded fragment      with Alapha     ---->kappam3     Usage:            result=CalculateKappaAlapha3(mol)            Input: mol is a molecule object.            Output: result is a numeric value.  #################################################################  **GetKappa**(mol)  #################################################################  Calculation of all kappa values.     Usage:            result=GetKappa(mol)            Input: mol is a molecule object.            Output: result is a dcit form containing 6 kappa values.  ################################################################# |

|  |  |  |
| --- | --- | --- |
| **Data** | | |
|  |  | **Version** = 1.0  **periodicTable** = <rdkit.Chem.rdchem.PeriodicTable object> |
