# Supplementary material for: Protein language model-based prediction for plant miRNA encoded peptides
Source: PeerJ Comput Sci. 2025 Mar 18;11:e2733. doi: 10.7717/peerj-cs.2733 (PMC11935769; doi:10.7717/peerj-cs.2733)
Supplement: Supplemental Information 1 [file peerj-cs-11-2733-s001.zip › pLM4PEP-master/pydpi/html/moe.html]

Python: module moe

|  |  |
| --- | --- |
| **moe** | index /home/orient/pydpi/drug/moe.py |

##############################################################################  
This module mainly implements the calculation of MOE-type descriptors, which   
   
include LabuteASA, TPSA, slogPVSA, MRVSA, PEOEVSA, EstateVSA and VSAEstate,   
   
respectively (59).  
   
If you have any question about these indices please contact me via email.  
   
My email adress is orientalcds@gmail.com   
   
Created on Tue May 24 10:29:47 2011  
   
@author: Dongsheng Cao  
##############################################################################

**Modules**

|  |  |  |  |
| --- | --- | --- | --- |
| rdkit.Chem | rdkit.Chem.EState.EState\_VSA | rdkit.Chem.MolSurf |  |

|  |  |  |
| --- | --- | --- |
| **Functions** | | |
|  |  | **CalculateEstateVSA**(mol, bins=None)  #################################################################  MOE-type descriptors using Estate indices and surface area      contributions.     estateBins=[-0.390,0.290,0.717,1.165,1.540,1.807,2.05,4.69,9.17,15.0]      You can specify your own bins to compute some descriptors     Usage:            result=CalculateEstateVSA(mol)            Input: mol is a molecule object            Output: result is a dict form   #################################################################  **CalculateLabuteASA**(mol)  #################################################################  Calculation of Labute's Approximate Surface Area (ASA from MOE)     Usage:            result=CalculateLabuteASA(mol)            Input: mol is a molecule object            Output: result is a dict form   #################################################################  **CalculatePEOEVSA**(mol, bins=None)  #################################################################  MOE-type descriptors using partial charges and surface      area contributions.     chgBins=[-.3,-.25,-.20,-.15,-.10,-.05,0,.05,.10,.15,.20,.25,.30]     You can specify your own bins to compute some descriptors     Usage:            result=CalculatePEOEVSA(mol)            Input: mol is a molecule object            Output: result is a dict form   #################################################################  **CalculateSLOGPVSA**(mol, bins=None)  #################################################################  MOE-type descriptors using LogP contributions and surface      area contributions.     logpBins=[-0.4,-0.2,0,0.1,0.15,0.2,0.25,0.3,0.4,0.5,0.6]     You can specify your own bins to compute some descriptors.     Usage:            result=CalculateSLOGPVSA(mol)            Input: mol is a molecule object            Output: result is a dict form   #################################################################  **CalculateSMRVSA**(mol, bins=None)  #################################################################  MOE-type descriptors using MR contributions and surface      area contributions.     mrBins=[1.29, 1.82, 2.24, 2.45, 2.75, 3.05, 3.63,3.8,4.0]     You can specify your own bins to compute some descriptors.     Usage:            result=CalculateSMRVSA(mol)            Input: mol is a molecule object            Output: result is a dict form   #################################################################  **CalculateTPSA**(mol)  #################################################################  Calculation of topological polar surface area based on fragments.     Implementation based on the Daylight contrib program tpsa.     Usage:            result=CalculateTPSA(mol)            Input: mol is a molecule object            Output: result is a dict form   #################################################################  **CalculateVSAEstate**(mol, bins=None)  #################################################################  MOE-type descriptors using Estate indices and surface      area contributions.     vsaBins=[4.78,5.00,5.410,5.740,6.00,6.07,6.45,7.00,11.0]      You can specify your own bins to compute some descriptors     Usage:            result=CalculateVSAEstate(mol)            Input: mol is a molecule object            Output: result is a dict form   #################################################################  **GetMOE**(mol)  #################################################################  The calculation of MOE-type descriptors (ALL).     Usage:            result=GetMOE(mol)            Input: mol is a molecule object            Output: result is a dict form   ################################################################# |

|  |  |  |
| --- | --- | --- |
| **Data** | | |
|  |  | **Version** = 1.0 |
