# Supplementary material for: Protein language model-based prediction for plant miRNA encoded peptides
Source: PeerJ Comput Sci. 2025 Mar 18;11:e2733. doi: 10.7717/peerj-cs.2733 (PMC11935769; doi:10.7717/peerj-cs.2733)
Supplement: Supplemental Information 1 [file peerj-cs-11-2733-s001.zip › pLM4PEP-master/pydpi/html/molproperty.html]

Python: module molproperty

|  |  |
| --- | --- |
| **molproperty** | index /home/orient/pydpi/drug/molproperty.py |

##############################################################################  
Calculation of Molecular physical/chemical properties based on some special   
   
type of approaches(6), including: LogP; LogP2; MR; TPSA, UI and Hy.You can   
   
freely use and distribute it. If you hava  any problem, you could contact   
   
with us timely!  
   
Authors: Dongsheng Cao and Yizeng Liang.  
   
Date: 2012.09.18  
   
Email: oriental-cds@163.com  
##############################################################################

**Modules**

|  |  |  |  |
| --- | --- | --- | --- |
| rdkit.Chem | rdkit.Chem.Crippen | rdkit.Chem.MolSurf | math |

|  |  |  |
| --- | --- | --- |
| **Functions** | | |
|  |  | **CalculateHydrophilicityFactor**(mol)  #################################################################  Calculation of hydrophilicity factor. The hydrophilicity      index is described in more detail on page 225 of the      Handbook of Molecular Descriptors (Todeschini and Consonni 2000).     ---->Hy     Usage:            result=CalculateHydrophilicityFactor(mol)            Input: mol is a molecule object.            Output: result is a numeric value.  #################################################################  **CalculateMolLogP**(mol)  #################################################################  Cacluation of LogP value based on Crippen method     ---->LogP     Usage:            result=CalculateMolLogP(mol)            Input: mol is a molecule object.            Output: result is a numeric value.  #################################################################  **CalculateMolLogP2**(mol)  #################################################################  Cacluation of LogP^2 value based on Crippen method     ---->LogP2     Usage:            result=CalculateMolLogP2(mol)            Input: mol is a molecule object.            Output: result is a numeric value.  #################################################################  **CalculateMolMR**(mol)  #################################################################  Cacluation of molecular refraction value based on Crippen method     ---->MR     Usage:            result=CalculateMolMR(mol)            Input: mol is a molecule object.            Output: result is a numeric value.  #################################################################  **CalculateTPSA**(mol)  #################################################################  calculates the polar surface area of a molecule based upon fragments     Algorithm in:        P. Ertl, B. Rohde, P. Selzer     Fast Calculation of Molecular Polar Surface Area as a Sum of      Fragment-based Contributions and Its Application to the Prediction     of Drug Transport Properties, J.Med.Chem. 43, 3714-3717, 2000     Implementation based on the Daylight contrib program tpsa.     ---->TPSA     Usage:            result=CalculateTPSA(mol)            Input: mol is a molecule object.            Output: result is a numeric value.  #################################################################  **CalculateUnsaturationIndex**(mol)  #################################################################  Calculation of unsaturation index.     ---->UI     Usage:            result=CalculateUnsaturationIndex(mol)            Input: mol is a molecule object.            Output: result is a numeric value.  #################################################################  **CalculateXlogP**(mol)  #################################################################  Calculation of Wang octanol water partition coefficient.     ---->XLogP     Usage:            result=CalculateXlogP(mol)            Input: mol is a molecule object.            Output: result is a numeric value.  #################################################################  **CalculateXlogP2**(mol)  #################################################################  Calculation of Wang octanol water partition coefficient (XLogP^2).     ---->XLogP2     Usage:            result=CalculateMolLogP(mol)            Input: mol is a molecule object.            Output: result is a numeric value.  #################################################################  **GetMolecularProperty**(mol)  #################################################################  Get the dictionary of constitutional descriptors for      given moelcule mol     Usage:            result=GetMolecularProperty(mol)            Input: mol is a molecule object.            Output: result is a dict form containing 6 molecular properties.  ################################################################# |

|  |  |  |
| --- | --- | --- |
| **Data** | | |
|  |  | **MolecularProperty** = {'Hy': <function CalculateHydrophilicityFactor>, 'LogP': <function CalculateMolLogP>, 'LogP2': <function CalculateMolLogP2>, 'MR': <function CalculateMolMR>, 'TPSA': <function CalculateTPSA>, 'UI': <function CalculateUnsaturationIndex>}  **Version** = 1.0 |
