# Supplementary material for: Protein language model-based prediction for plant miRNA encoded peptides
Source: PeerJ Comput Sci. 2025 Mar 18;11:e2733. doi: 10.7717/peerj-cs.2733 (PMC11935769; doi:10.7717/peerj-cs.2733)
Supplement: Supplemental Information 1 [file peerj-cs-11-2733-s001.zip › pLM4PEP-master/pydpi/html/moran.html]

Python: module moran

|  |  |
| --- | --- |
| **moran** | index /home/orient/pydpi/drug/moran.py |

##############################################################################  
   
The calculation of Moran autocorrelation descriptors. You can get 32 molecular  
   
decriptors. You can freely use and distribute it. If you hava  any problem,   
   
you could contact with us timely!  
   
Authors: Dongsheng Cao and Yizeng Liang.  
   
Date: 2012.09.18  
   
Email: oriental-cds@163.com  
   
##############################################################################

**Modules**

|  |  |  |  |
| --- | --- | --- | --- |
| rdkit.Chem | numpy |  |  |

|  |  |  |
| --- | --- | --- |
| **Functions** | | |
|  |  | **CalculateMoranAutoElectronegativity**(mol)  #################################################################  Calculation of Moran autocorrelation descriptors based on      carbon-scaled atomic Sanderson electronegativity.     Usage:     res=CalculateMoranAutoElectronegativity(mol)     Input: mol is a molecule object.     Output: res is a dict form containing eight moran autocorrealtion     descriptors.  #################################################################  **CalculateMoranAutoMass**(mol)  #################################################################  Calculation of Moran autocorrelation descriptors based on      carbon-scaled atomic mass.     Usage:     res=CalculateMoranAutoMass(mol)     Input: mol is a molecule object.     Output: res is a dict form containing eight moran autocorrealtion     descriptors.  #################################################################  **CalculateMoranAutoPolarizability**(mol)  #################################################################  Calculation of Moran autocorrelation descriptors based on      carbon-scaled atomic polarizability.     Usage:     res=CalculateMoranAutoPolarizability(mol)     Input: mol is a molecule object.     Output: res is a dict form containing eight moran autocorrealtion     descriptors.  #################################################################  **CalculateMoranAutoVolume**(mol)  #################################################################  Calculation of Moran autocorrelation descriptors based on      carbon-scaled atomic van der Waals volume.     Usage:     res=CalculateMoranAutoVolume(mol)     Input: mol is a molecule object.     Output: res is a dict form containing eight moran autocorrealtion     descriptors.  #################################################################  **GetMoranAuto**(mol)  #################################################################  Calcualate all Moran autocorrelation descriptors.     (carbon-scaled atomic mass, carbon-scaled atomic van der Waals volume,     carbon-scaled atomic Sanderson electronegativity,     carbon-scaled atomic polarizability)     Usage:     res=GetMoranAuto(mol)     Input: mol is a molecule object.     Output: res is a dict form containing all moran autocorrealtion     descriptors.  ################################################################# |

|  |  |  |
| --- | --- | --- |
| **Data** | | |
|  |  | **Version** = 1.0 |
