# Supplementary material for: Protein language model-based prediction for plant miRNA encoded peptides
Source: PeerJ Comput Sci. 2025 Mar 18;11:e2733. doi: 10.7717/peerj-cs.2733 (PMC11935769; doi:10.7717/peerj-cs.2733)
Supplement: Supplemental Information 1 [file peerj-cs-11-2733-s001.zip › pLM4PEP-master/pydpi/html/moreaubroto.html]

Python: module moreaubroto

|  |  |
| --- | --- |
| **moreaubroto** | index /home/orient/pydpi/drug/moreaubroto.py |

##############################################################################  
   
The calculation of Moreau-Broto autocorrelation descriptors. You can get 32  
   
molecular decriptors. You can freely use and distribute it. If you hava    
   
any problem, you could contact with us timely!  
   
Authors: Dongsheng Cao and Yizeng Liang.  
   
Date: 2012.09.18  
   
Email: oriental-cds@163.com  
   
##############################################################################

**Modules**

|  |  |  |  |
| --- | --- | --- | --- |
| rdkit.Chem | numpy |  |  |

|  |  |  |
| --- | --- | --- |
| **Functions** | | |
|  |  | **CalculateMoreauBrotoAutoElectronegativity**(mol)  #################################################################  Calculation of Moreau-Broto autocorrelation descriptors based on      carbon-scaled atomic Sanderson electronegativity.     Usage:      res=CalculateMoreauBrotoAutoElectronegativity(mol)     Input: mol is a molcule object.     Output: res is a dict form containing eight moreau broto autocorrealtion     descriptors.  #################################################################  **CalculateMoreauBrotoAutoMass**(mol)  #################################################################  Calculation of Moreau-Broto autocorrelation descriptors based on      carbon-scaled atomic mass.     Usage:     res=CalculateMoreauBrotoAutoMass(mol)     Input: mol is a molecule object.     Output: res is a dict form containing eight moreau broto autocorrealtion     descriptors.  #################################################################  **CalculateMoreauBrotoAutoPolarizability**(mol)  #################################################################  Calculation of Moreau-Broto autocorrelation descriptors based on      carbon-scaled atomic polarizability.     res=CalculateMoreauBrotoAutoPolarizability(mol)     Input: mol is a molcule object.     Output: res is a dict form containing eight moreau broto autocorrealtion     descriptors.  #################################################################  **CalculateMoreauBrotoAutoVolume**(mol)  #################################################################  Calculation of Moreau-Broto autocorrelation descriptors based on      carbon-scaled atomic van der Waals volume.     Usage:      res=CalculateMoreauBrotoAutoVolume(mol)     Input: mol is a molcule object.     Output: res is a dict form containing eight moreau broto autocorrealtion     descriptors.  #################################################################  **GetMoreauBrotoAuto**(mol)  #################################################################  Calcualate all Moreau-Broto autocorrelation descriptors.      (carbon-scaled atomic mass, carbon-scaled atomic van der Waals volume,     carbon-scaled atomic Sanderson electronegativity,     carbon-scaled atomic polarizability)     Usage:     res=GetMoreauBrotoAuto(mol)     Input: mol is a molecule object.     Output: res is a dict form containing all moreau broto autocorrelation     descriptors.  ################################################################# |

|  |  |  |
| --- | --- | --- |
| **Data** | | |
|  |  | **Version** = 1.0 |
