# Supplementary material for: Protein language model-based prediction for plant miRNA encoded peptides
Source: PeerJ Comput Sci. 2025 Mar 18;11:e2733. doi: 10.7717/peerj-cs.2733 (PMC11935769; doi:10.7717/peerj-cs.2733)
Supplement: Supplemental Information 1 [file peerj-cs-11-2733-s001.zip › pLM4PEP-master/pydpi/html/pydpi.html]

Python: module pydpi

|  |  |
| --- | --- |
| **pydpi** | index /home/orient/pydpi/src/pydpi/pydpi.py |

##############################################################################  
   
A class used for computing drug-target interaction, protein-protein interaction  
   
features. The PyDPI class inherits the PyDrug and PyPro classes, So you can easily  
   
use them. You can freely use and distribute it. If you hava  any problem,   
   
you could contact with us timely!  
   
Authors: Dongsheng Cao and Yizeng Liang.  
   
Date: 2012.09.24  
   
Email: oriental-cds@163.com  
   
##############################################################################

**Modules**

|  |  |  |  |
| --- | --- | --- | --- |
| protein.AAComposition  protein.Autocorrelation  protein.CTD  rdkit.Chem  protein.ConjointTriad  protein.PseudoAAC | protein.QuasiSequenceOrder  drug.charge  drug.connectivity  drug.constitution  drug.estate  drug.fingerprint | drug.geary  drug.getmol  drug.kappa  drug.moe  drug.molproperty  drug.moran | drug.moreaubroto  string  drug.topology |

**Classes**

pydrug.PyDrug
:   PyDPI(pydrug.PyDrug, pypro.PyPro)

pypro.PyPro
:   PyDPI(pydrug.PyDrug, pypro.PyPro)

    PyPPI

|  |  |  |
| --- | --- | --- |
| class **PyDPI**(pydrug.PyDrug, pypro.PyPro) | | |
|  | #################################################################     A PyDPI class used for generating drug-target interaction features.     ################################################################# | |
|  |  | Method resolution order:  PyDPI  pydrug.PyDrug  pypro.PyPro   ---  Methods defined here:  **GetDPIFeature1**(self, ddict={}, pdict={})  #################################################################     Calculate the drug-target interaction features by combining drug      features and protein features.(nd+np)     Usage:            res=GetDPIFeature1(ddict,pdict)            Input: ddict is a dict form containing drug features.                   pdict is a dict form containing protein features.                   Output: res is a dict form containing drug-target interaction            features.        #################################################################  **GetDPIFeature2**(self, ddict={}, pdict={})  #################################################################  Calculate the drug-target interaction features by  the tensor product.     (nd\*np)     Usage:            res=GetDPIFeature2(ddict,pdict)            Input: ddict is a dict form containing drug features.                   pdict is a dict form containing protein features.                   Output: res is a dict form containing drug-target interaction            features.  #################################################################  **\_\_init\_\_**(self)  #################################################################     constructor of PyDPI.     #################################################################   ---  Methods inherited from pydrug.PyDrug:  **GetAllDescriptor**(self)  #################################################################  Calculate all descriptors (608).     Usage:            res=GetAllDescriptor()            res is a dict form.  #################################################################  **GetCharge**(self)  #################################################################  Calculate all charge descriptors (25).     Usage:            res=GetCharge()            res is a dict form.  #################################################################  **GetConnectivity**(self)  #################################################################  Calculate all conenctivity descriptors (44).     Usage:            res=GetConnectivity()            res is a dict form.  #################################################################  **GetConstitution**(self)  #################################################################  Calculate all constitutional descriptors (30).     Usage:            res=GetConstitution()            res is a dict form.  #################################################################  **GetEstate**(self)  #################################################################  Calculate estate descriptors (316).     Usage:            res=GetEstate()            res is a dict form.  #################################################################  **GetFingerprint**(self, FPName='topological')  #################################################################  Calculate all fingerprint descriptors.     see the fingerprint type in FingerprintName     Usage:            res=GetFingerprint(FPName='topological')            res is a tuple form.  #################################################################  **GetGeary**(self)  #################################################################  Calculate all Geary autocorrelation descriptors (32).     Usage:            res=GetGeary()            res is a dict form.  #################################################################  **GetKappa**(self)  #################################################################  Calculate all kappa descriptors (7).     Usage:            res=GetKappa()            res is a dict form.  #################################################################  **GetMOE**(self)  #################################################################  Calculate all MOE-type descriptors (60).     Usage:            res=GetMOE()            res is a dict form.  #################################################################  **GetMolFromCAS**(self, ID='')  #################################################################  Get a molecule by kegg id (e.g., 50-29-3).     Usage:            res=GetMolFromCAS(ID)            Input: ID is a CAS identifier.            Output: res is a SMILES string.  #################################################################  **GetMolFromDrugbank**(self, ID='')  #################################################################  Get a molecule by drugbank id (e.g.,DB00133).     Usage:            res=GetMolFromDrugbank(ID)            Input: ID is a compound identifier in Drugbank.            Output: res is a SMILES string.  #################################################################  **GetMolFromEBI**(self, ID='')  #################################################################  Get a molecule by EBI id.     Usage:            res=GetMolFromEBI(ID)            Input: ID is a compound identifier in EBI.            Output: res is a SMILES string.  #################################################################  **GetMolFromKegg**(self, ID='')  #################################################################  Get a molecule by kegg id (e.g., D02176).     Usage:            res=GetMolFromKegg(ID)            Input: ID is a compound identifier in KEGG.            Output: res is a SMILES string.  #################################################################  **GetMolFromNCBI**(self, ID='')  #################################################################  Get a molecule by NCBI id (e.g., 2244).     Usage:            res=GetMolFromNCBI(ID)            Input: ID is a compound ID (CID) in NCBI.            Output: res is a SMILES string.  #################################################################  **GetMolProperty**(self)  #################################################################  Calculate all molecular properties (6).     Usage:            res=GetMolProperty()            res is a dict form.  #################################################################  **GetMoran**(self)  #################################################################  Calculate all Moran autocorrealtion descriptors (32).     Usage:            res=GetMoran()            res is a dict form.  #################################################################  **GetMoreauBroto**(self)  #################################################################  Calculate all Moreau-Broto autocorrelation descriptors(32).     Usage:            res=GetMoreauBroto()            res is a dict form.  #################################################################  **GetTopology**(self)  #################################################################  Calculate all topological descriptors (25).     Usage:            res=GetTopology()            res is a dict form.  #################################################################  **ReadMolFromFile**(self, filename='')  #################################################################  Read a molecule by SDF or MOL file format.     Usage:            res=ReadMolFromFile(filename)            Input: filename is a file name.            Output: res is a molecule object.  #################################################################  **ReadMolFromInchi**(self, inchi='')  #################################################################  Read a molecule by Inchi string.     Usage:            res=ReadMolFromInchi(inchi)            Input: inchi is a InChi string.            Output: res is a molecule object.  #################################################################  **ReadMolFromMol**(self, filename='')  #################################################################  Read a molecule with mol file format.     Usage:            res=ReadMolFromMol(filename)            Input: filename is a file name.            Output: res is a molecule object.  #################################################################  **ReadMolFromSmile**(self, smi='')  #################################################################  Read a molecule by SMILES string.     Usage:            res=ReadMolFromSmile(smi)            Input: smi is a SMILES string.            Output: res is a molecule object.  #################################################################   ---  Methods inherited from pypro.PyPro:  **GetAAComp**(self)  amino acid compositon descriptors (20)     Usage:     result = GetAAComp()  **GetAAindex1**(self, name, path='.')  Get the amino acid property values from aaindex1     Usage:     result=GetAAIndex1(name)     Input: name is the name of amino acid property (e.g., KRIW790103)     Output: result is a dict form containing the properties of 20 amino acids  **GetAAindex23**(self, name, path='.')  Get the amino acid property values from aaindex2 and aaindex3     Usage:     result=GetAAIndex23(name)     Input: name is the name of amino acid property (e.g.,TANS760101,GRAR740104)     Output: result is a dict form containing the properties of 400 amino acid pairs  **GetALL**(self)  Calcualte all descriptors except tri-peptide descriptors  **GetAPAAC**(self, lamda=10, weight=0.5)  Amphiphilic (Type II) Pseudo amino acid composition descriptors      default is 30     Usage:     result = GetAPAAC(lamda=10,weight=0.5)     lamda factor reflects the rank of correlation and is a non-Negative integer, such as 15.     Note that (1)lamda should NOT be larger than the length of input protein sequence;     (2) lamda must be non-Negative integer, such as 0, 1, 2, ...; (3) when lamda =0, the      output of PseAA server is the 20-D amino acid composition.     weight factor is designed for the users to put weight on the additional PseAA components      with respect to the conventional AA components. The user can select any value within the      region from 0.05 to 0.7 for the weight factor.  **GetCTD**(self)  Composition Transition Distribution descriptors (147)     Usage:     result = GetCTD()  **GetDPComp**(self)  dipeptide composition descriptors (400)      Usage:     result = GetDPComp()  **GetGearyAuto**(self)  Geary autocorrelation descriptors (240)     Usage:     result = GetGearyAuto()  **GetGearyAutop**(self, AAP={}, AAPName='p')  Geary autocorrelation descriptors for the given property (30)     Usage:     result = GetGearyAutop(AAP={},AAPName='p')     AAP is a dict containing physicochemical properities of 20 amino acids  **GetMoranAuto**(self)  Moran autocorrelation descriptors (240)     Usage:     result = GetMoranAuto()  **GetMoranAutop**(self, AAP={}, AAPName='p')  Moran autocorrelation descriptors for the given property (30)     Usage:     result = GetMoranAutop(AAP={},AAPName='p')     AAP is a dict containing physicochemical properities of 20 amino acids  **GetMoreauBrotoAuto**(self)  Normalized Moreau-Broto autocorrelation descriptors (240)     Usage:     result = GetMoreauBrotoAuto()  **GetMoreauBrotoAutop**(self, AAP={}, AAPName='p')  Normalized Moreau-Broto autocorrelation descriptors for the given property (30)     Usage:     result = GetMoreauBrotoAutop(AAP={},AAPName='p')     AAP is a dict containing physicochemical properities of 20 amino acids  **GetPAAC**(self, lamda=10, weight=0.05)  Type I Pseudo amino acid composition descriptors (default is 30)     Usage:     result = GetPAAC(lamda=10,weight=0.05)     lamda factor reflects the rank of correlation and is a non-Negative integer, such as 15.     Note that (1)lamda should NOT be larger than the length of input protein sequence;     (2) lamda must be non-Negative integer, such as 0, 1, 2, ...; (3) when lamda =0, the      output of PseAA server is the 20-D amino acid composition.     weight factor is designed for the users to put weight on the additional PseAA components      with respect to the conventional AA components. The user can select any value within the      region from 0.05 to 0.7 for the weight factor.  **GetPAACp**(self, lamda=10, weight=0.05, AAP=[])  Type I Pseudo amino acid composition descriptors for the given properties (default is 30)     Usage:     result = GetPAACp(lamda=10,weight=0.05,AAP=[])     lamda factor reflects the rank of correlation and is a non-Negative integer, such as 15.     Note that (1)lamda should NOT be larger than the length of input protein sequence;     (2) lamda must be non-Negative integer, such as 0, 1, 2, ...; (3) when lamda =0, the      output of PseAA server is the 20-D amino acid composition.     weight factor is designed for the users to put weight on the additional PseAA components      with respect to the conventional AA components. The user can select any value within the      region from 0.05 to 0.7 for the weight factor.     AAP is a list form containing the properties, each of which is a dict form.  **GetProteinSequenceFromID**(self, uniprotid='')  Downloading a protein sequence by uniprot id.  **GetQSO**(self, maxlag=30, weight=0.1)  Quasi sequence order descriptors  default is 50     result = GetQSO(maxlag=30, weight=0.1)     maxlag is the maximum lag and the length of the protein should be larger     than maxlag. default is 45.  **GetQSOp**(self, maxlag=30, weight=0.1, distancematrix={})  Quasi sequence order descriptors  default is 50     result = GetQSO(maxlag=30, weight=0.1)     maxlag is the maximum lag and the length of the protein should be larger     than maxlag. default is 45.     distancematrix is a dict form containing 400 distance values  **GetSOCN**(self, maxlag=45)  Sequence order coupling numbers  default is 45     Usage:     result = GetSOCN(maxlag=45)     maxlag is the maximum lag and the length of the protein should be larger     than maxlag. default is 45.  **GetSOCNp**(self, maxlag=45, distancematrix={})  Sequence order coupling numbers  default is 45     Usage:     result = GetSOCN(maxlag=45)     maxlag is the maximum lag and the length of the protein should be larger     than maxlag. default is 45.     distancematrix is a dict form containing 400 distance values  **GetSubSeq**(self, ToAA='S', window=3)  obtain the sub sequences wit length 2\*window+1, whose central point is ToAA     Usage:     result = GetSubSeq(ToAA='S',window=3)     ToAA is the central (query point) amino acid in the sub-sequence.     window is the span.  **GetTPComp**(self)  tri-peptide composition descriptors (8000)     Usage:     result = GetTPComp()  **GetTriad**(self)  Calculate the conjoint triad features from protein sequence.     Useage:     res = GetTriad()     Output is a dict form containing all 343 conjoint triad features.  **ReadProteinSequence**(self, ProteinSequence='')  Read a protein sequence.   ---  Data and other attributes inherited from pypro.PyPro:  **AALetter** = ['A', 'R', 'N', 'D', 'C', 'E', 'Q', 'G', 'H', 'I', 'L', 'K', 'M', 'F', 'P', 'S', 'T', 'W', 'Y', 'V']  **Version** = 1.0 |

|  |  |  |
| --- | --- | --- |
| class **PyPPI**(pypro.PyPro) | | |
|  | #################################################################     A PyPPI class used for generating protein-protein interaction features.     ################################################################# | |
|  |  | Methods defined here:  **GetPPIFeature1**(self, pdict={})  #################################################################  Calculate the protein-protein interaction features by      F=[Fa(i)+Fb(i)),Fa(i)\*Fb(i)] (2n)     Usage:            res=GetPPIFeature1(pdict)            Input: pdict is a dict form containing protein features.                   Output: res is a dict form containing protein-protein interaction            features.  #################################################################  **GetPPIFeature2**(self, pdict={})  #################################################################  Calculate the protein-protein interaction features by the tensor product.     (n^2)     Usage:            res=GetPPIFeature2(pdict)            Input: pdict is a dict form containing protein features.                   Output: res is a dict form containing protein-protein interaction            features.  #################################################################  **\_\_init\_\_**(self)  #################################################################     constructor of PyPPI.     #################################################################   ---  Methods inherited from pypro.PyPro:  **GetAAComp**(self)  amino acid compositon descriptors (20)     Usage:     result = GetAAComp()  **GetAAindex1**(self, name, path='.')  Get the amino acid property values from aaindex1     Usage:     result=GetAAIndex1(name)     Input: name is the name of amino acid property (e.g., KRIW790103)     Output: result is a dict form containing the properties of 20 amino acids  **GetAAindex23**(self, name, path='.')  Get the amino acid property values from aaindex2 and aaindex3     Usage:     result=GetAAIndex23(name)     Input: name is the name of amino acid property (e.g.,TANS760101,GRAR740104)     Output: result is a dict form containing the properties of 400 amino acid pairs  **GetALL**(self)  Calcualte all descriptors except tri-peptide descriptors  **GetAPAAC**(self, lamda=10, weight=0.5)  Amphiphilic (Type II) Pseudo amino acid composition descriptors      default is 30     Usage:     result = GetAPAAC(lamda=10,weight=0.5)     lamda factor reflects the rank of correlation and is a non-Negative integer, such as 15.     Note that (1)lamda should NOT be larger than the length of input protein sequence;     (2) lamda must be non-Negative integer, such as 0, 1, 2, ...; (3) when lamda =0, the      output of PseAA server is the 20-D amino acid composition.     weight factor is designed for the users to put weight on the additional PseAA components      with respect to the conventional AA components. The user can select any value within the      region from 0.05 to 0.7 for the weight factor.  **GetCTD**(self)  Composition Transition Distribution descriptors (147)     Usage:     result = GetCTD()  **GetDPComp**(self)  dipeptide composition descriptors (400)      Usage:     result = GetDPComp()  **GetGearyAuto**(self)  Geary autocorrelation descriptors (240)     Usage:     result = GetGearyAuto()  **GetGearyAutop**(self, AAP={}, AAPName='p')  Geary autocorrelation descriptors for the given property (30)     Usage:     result = GetGearyAutop(AAP={},AAPName='p')     AAP is a dict containing physicochemical properities of 20 amino acids  **GetMoranAuto**(self)  Moran autocorrelation descriptors (240)     Usage:     result = GetMoranAuto()  **GetMoranAutop**(self, AAP={}, AAPName='p')  Moran autocorrelation descriptors for the given property (30)     Usage:     result = GetMoranAutop(AAP={},AAPName='p')     AAP is a dict containing physicochemical properities of 20 amino acids  **GetMoreauBrotoAuto**(self)  Normalized Moreau-Broto autocorrelation descriptors (240)     Usage:     result = GetMoreauBrotoAuto()  **GetMoreauBrotoAutop**(self, AAP={}, AAPName='p')  Normalized Moreau-Broto autocorrelation descriptors for the given property (30)     Usage:     result = GetMoreauBrotoAutop(AAP={},AAPName='p')     AAP is a dict containing physicochemical properities of 20 amino acids  **GetPAAC**(self, lamda=10, weight=0.05)  Type I Pseudo amino acid composition descriptors (default is 30)     Usage:     result = GetPAAC(lamda=10,weight=0.05)     lamda factor reflects the rank of correlation and is a non-Negative integer, such as 15.     Note that (1)lamda should NOT be larger than the length of input protein sequence;     (2) lamda must be non-Negative integer, such as 0, 1, 2, ...; (3) when lamda =0, the      output of PseAA server is the 20-D amino acid composition.     weight factor is designed for the users to put weight on the additional PseAA components      with respect to the conventional AA components. The user can select any value within the      region from 0.05 to 0.7 for the weight factor.  **GetPAACp**(self, lamda=10, weight=0.05, AAP=[])  Type I Pseudo amino acid composition descriptors for the given properties (default is 30)     Usage:     result = GetPAACp(lamda=10,weight=0.05,AAP=[])     lamda factor reflects the rank of correlation and is a non-Negative integer, such as 15.     Note that (1)lamda should NOT be larger than the length of input protein sequence;     (2) lamda must be non-Negative integer, such as 0, 1, 2, ...; (3) when lamda =0, the      output of PseAA server is the 20-D amino acid composition.     weight factor is designed for the users to put weight on the additional PseAA components      with respect to the conventional AA components. The user can select any value within the      region from 0.05 to 0.7 for the weight factor.     AAP is a list form containing the properties, each of which is a dict form.  **GetProteinSequenceFromID**(self, uniprotid='')  Downloading a protein sequence by uniprot id.  **GetQSO**(self, maxlag=30, weight=0.1)  Quasi sequence order descriptors  default is 50     result = GetQSO(maxlag=30, weight=0.1)     maxlag is the maximum lag and the length of the protein should be larger     than maxlag. default is 45.  **GetQSOp**(self, maxlag=30, weight=0.1, distancematrix={})  Quasi sequence order descriptors  default is 50     result = GetQSO(maxlag=30, weight=0.1)     maxlag is the maximum lag and the length of the protein should be larger     than maxlag. default is 45.     distancematrix is a dict form containing 400 distance values  **GetSOCN**(self, maxlag=45)  Sequence order coupling numbers  default is 45     Usage:     result = GetSOCN(maxlag=45)     maxlag is the maximum lag and the length of the protein should be larger     than maxlag. default is 45.  **GetSOCNp**(self, maxlag=45, distancematrix={})  Sequence order coupling numbers  default is 45     Usage:     result = GetSOCN(maxlag=45)     maxlag is the maximum lag and the length of the protein should be larger     than maxlag. default is 45.     distancematrix is a dict form containing 400 distance values  **GetSubSeq**(self, ToAA='S', window=3)  obtain the sub sequences wit length 2\*window+1, whose central point is ToAA     Usage:     result = GetSubSeq(ToAA='S',window=3)     ToAA is the central (query point) amino acid in the sub-sequence.     window is the span.  **GetTPComp**(self)  tri-peptide composition descriptors (8000)     Usage:     result = GetTPComp()  **GetTriad**(self)  Calculate the conjoint triad features from protein sequence.     Useage:     res = GetTriad()     Output is a dict form containing all 343 conjoint triad features.  **ReadProteinSequence**(self, ProteinSequence='')  Read a protein sequence.   ---  Data and other attributes inherited from pypro.PyPro:  **AALetter** = ['A', 'R', 'N', 'D', 'C', 'E', 'Q', 'G', 'H', 'I', 'L', 'K', 'M', 'F', 'P', 'S', 'T', 'W', 'Y', 'V']  **Version** = 1.0 |

|  |  |  |
| --- | --- | --- |
| **Data** | | |
|  |  | **FingerprintName** = ['topological', 'Estate', 'FP4', 'atompairs', 'torsions', 'morgan', 'MACCS']  **Version** = 1.0 |
