# Supplementary material for: Protein language model-based prediction for plant miRNA encoded peptides
Source: PeerJ Comput Sci. 2025 Mar 18;11:e2733. doi: 10.7717/peerj-cs.2733 (PMC11935769; doi:10.7717/peerj-cs.2733)
Supplement: Supplemental Information 1 [file peerj-cs-11-2733-s001.zip › pLM4PEP-master/pydpi/html/pydrug.html]

Python: module pydrug

|  |  |
| --- | --- |
| **pydrug** | index /home/orient/pydpi/src/pydpi/pydrug.py |

##############################################################################  
   
A class used for computing different types of drug descriptors!   
   
You can freely use and distribute it. If you have any problem,   
   
you could contact with us timely.  
   
Authors: Dongsheng Cao and Yizeng Liang.  
   
Date: 2012.09.24  
   
Email: oriental-cds@163.com  
   
##############################################################################

**Modules**

|  |  |  |  |
| --- | --- | --- | --- |
| rdkit.Chem  drug.charge  drug.connectivity  drug.constitution | drug.estate  drug.fingerprint  drug.geary  drug.getmol | drug.kappa  drug.moe  drug.molproperty  drug.moran | drug.moreaubroto  string  drug.topology |

**Classes**

PyDrug

|  |  |  |
| --- | --- | --- |
| class **PyDrug** | | |
|  | #################################################################  A PyDrug class used for computing drug descriptors.  ################################################################# | |
|  |  | Methods defined here:  **GetAllDescriptor**(self)  #################################################################  Calculate all descriptors (608).     Usage:            res=GetAllDescriptor()            res is a dict form.  #################################################################  **GetCharge**(self)  #################################################################  Calculate all charge descriptors (25).     Usage:            res=GetCharge()            res is a dict form.  #################################################################  **GetConnectivity**(self)  #################################################################  Calculate all conenctivity descriptors (44).     Usage:            res=GetConnectivity()            res is a dict form.  #################################################################  **GetConstitution**(self)  #################################################################  Calculate all constitutional descriptors (30).     Usage:            res=GetConstitution()            res is a dict form.  #################################################################  **GetEstate**(self)  #################################################################  Calculate estate descriptors (316).     Usage:            res=GetEstate()            res is a dict form.  #################################################################  **GetFingerprint**(self, FPName='topological')  #################################################################  Calculate all fingerprint descriptors.     see the fingerprint type in FingerprintName     Usage:            res=GetFingerprint(FPName='topological')            res is a tuple form.  #################################################################  **GetGeary**(self)  #################################################################  Calculate all Geary autocorrelation descriptors (32).     Usage:            res=GetGeary()            res is a dict form.  #################################################################  **GetKappa**(self)  #################################################################  Calculate all kappa descriptors (7).     Usage:            res=GetKappa()            res is a dict form.  #################################################################  **GetMOE**(self)  #################################################################  Calculate all MOE-type descriptors (60).     Usage:            res=GetMOE()            res is a dict form.  #################################################################  **GetMolFromCAS**(self, ID='')  #################################################################  Get a molecule by kegg id (e.g., 50-29-3).     Usage:            res=GetMolFromCAS(ID)            Input: ID is a CAS identifier.            Output: res is a SMILES string.  #################################################################  **GetMolFromDrugbank**(self, ID='')  #################################################################  Get a molecule by drugbank id (e.g.,DB00133).     Usage:            res=GetMolFromDrugbank(ID)            Input: ID is a compound identifier in Drugbank.            Output: res is a SMILES string.  #################################################################  **GetMolFromEBI**(self, ID='')  #################################################################  Get a molecule by EBI id.     Usage:            res=GetMolFromEBI(ID)            Input: ID is a compound identifier in EBI.            Output: res is a SMILES string.  #################################################################  **GetMolFromKegg**(self, ID='')  #################################################################  Get a molecule by kegg id (e.g., D02176).     Usage:            res=GetMolFromKegg(ID)            Input: ID is a compound identifier in KEGG.            Output: res is a SMILES string.  #################################################################  **GetMolFromNCBI**(self, ID='')  #################################################################  Get a molecule by NCBI id (e.g., 2244).     Usage:            res=GetMolFromNCBI(ID)            Input: ID is a compound ID (CID) in NCBI.            Output: res is a SMILES string.  #################################################################  **GetMolProperty**(self)  #################################################################  Calculate all molecular properties (6).     Usage:            res=GetMolProperty()            res is a dict form.  #################################################################  **GetMoran**(self)  #################################################################  Calculate all Moran autocorrealtion descriptors (32).     Usage:            res=GetMoran()            res is a dict form.  #################################################################  **GetMoreauBroto**(self)  #################################################################  Calculate all Moreau-Broto autocorrelation descriptors(32).     Usage:            res=GetMoreauBroto()            res is a dict form.  #################################################################  **GetTopology**(self)  #################################################################  Calculate all topological descriptors (25).     Usage:            res=GetTopology()            res is a dict form.  #################################################################  **ReadMolFromFile**(self, filename='')  #################################################################  Read a molecule by SDF or MOL file format.     Usage:            res=ReadMolFromFile(filename)            Input: filename is a file name.            Output: res is a molecule object.  #################################################################  **ReadMolFromInchi**(self, inchi='')  #################################################################  Read a molecule by Inchi string.     Usage:            res=ReadMolFromInchi(inchi)            Input: inchi is a InChi string.            Output: res is a molecule object.  #################################################################  **ReadMolFromMol**(self, filename='')  #################################################################  Read a molecule with mol file format.     Usage:            res=ReadMolFromMol(filename)            Input: filename is a file name.            Output: res is a molecule object.  #################################################################  **ReadMolFromSmile**(self, smi='')  #################################################################  Read a molecule by SMILES string.     Usage:            res=ReadMolFromSmile(smi)            Input: smi is a SMILES string.            Output: res is a molecule object.  #################################################################  **\_\_init\_\_**(self)  #################################################################  constructor of pydrug.  ################################################################# |

|  |  |  |
| --- | --- | --- |
| **Data** | | |
|  |  | **FingerprintName** = ['topological', 'Estate', 'FP4', 'atompairs', 'torsions', 'morgan', 'MACCS']  **Version** = 1.0 |
