# Supplementary material for: Protein language model-based prediction for plant miRNA encoded peptides
Source: PeerJ Comput Sci. 2025 Mar 18;11:e2733. doi: 10.7717/peerj-cs.2733 (PMC11935769; doi:10.7717/peerj-cs.2733)
Supplement: Supplemental Information 1 [file peerj-cs-11-2733-s001.zip › pLM4PEP-master/pydpi/html/pypro.html]

Python: module pypro

|  |  |
| --- | --- |
| **pypro** | index /home/orient/pydpi/src/pydpi/pypro.py |

##############################################################################  
   
A class used for computing different types of protein descriptors!   
   
You can freely use and distribute it. If you have any problem,   
   
you could contact with us timely.  
   
Authors: Dongsheng Cao and Yizeng Liang.  
   
Date: 2012.9.4  
   
Email: oriental-cds@163.com  
   
##############################################################################

**Modules**

|  |  |  |  |
| --- | --- | --- | --- |
| protein.AAComposition  protein.Autocorrelation | protein.CTD  protein.ConjointTriad | protein.PseudoAAC  protein.QuasiSequenceOrder | string |

**Classes**

PyPro

|  |  |  |
| --- | --- | --- |
| class **PyPro** | | |
|  | This GetProDes class aims at collecting all descriptor calcualtion modules into a simple class. | |
|  |  | Methods defined here:  **GetAAComp**(self)  amino acid compositon descriptors (20)     Usage:     result = GetAAComp()  **GetAAindex1**(self, name, path='.')  Get the amino acid property values from aaindex1     Usage:     result=GetAAIndex1(name)     Input: name is the name of amino acid property (e.g., KRIW790103)     Output: result is a dict form containing the properties of 20 amino acids  **GetAAindex23**(self, name, path='.')  Get the amino acid property values from aaindex2 and aaindex3     Usage:     result=GetAAIndex23(name)     Input: name is the name of amino acid property (e.g.,TANS760101,GRAR740104)     Output: result is a dict form containing the properties of 400 amino acid pairs  **GetALL**(self)  Calcualte all descriptors except tri-peptide descriptors  **GetAPAAC**(self, lamda=10, weight=0.5)  Amphiphilic (Type II) Pseudo amino acid composition descriptors      default is 30     Usage:     result = GetAPAAC(lamda=10,weight=0.5)     lamda factor reflects the rank of correlation and is a non-Negative integer, such as 15.     Note that (1)lamda should NOT be larger than the length of input protein sequence;     (2) lamda must be non-Negative integer, such as 0, 1, 2, ...; (3) when lamda =0, the      output of PseAA server is the 20-D amino acid composition.     weight factor is designed for the users to put weight on the additional PseAA components      with respect to the conventional AA components. The user can select any value within the      region from 0.05 to 0.7 for the weight factor.  **GetCTD**(self)  Composition Transition Distribution descriptors (147)     Usage:     result = GetCTD()  **GetDPComp**(self)  dipeptide composition descriptors (400)      Usage:     result = GetDPComp()  **GetGearyAuto**(self)  Geary autocorrelation descriptors (240)     Usage:     result = GetGearyAuto()  **GetGearyAutop**(self, AAP={}, AAPName='p')  Geary autocorrelation descriptors for the given property (30)     Usage:     result = GetGearyAutop(AAP={},AAPName='p')     AAP is a dict containing physicochemical properities of 20 amino acids  **GetMoranAuto**(self)  Moran autocorrelation descriptors (240)     Usage:     result = GetMoranAuto()  **GetMoranAutop**(self, AAP={}, AAPName='p')  Moran autocorrelation descriptors for the given property (30)     Usage:     result = GetMoranAutop(AAP={},AAPName='p')     AAP is a dict containing physicochemical properities of 20 amino acids  **GetMoreauBrotoAuto**(self)  Normalized Moreau-Broto autocorrelation descriptors (240)     Usage:     result = GetMoreauBrotoAuto()  **GetMoreauBrotoAutop**(self, AAP={}, AAPName='p')  Normalized Moreau-Broto autocorrelation descriptors for the given property (30)     Usage:     result = GetMoreauBrotoAutop(AAP={},AAPName='p')     AAP is a dict containing physicochemical properities of 20 amino acids  **GetPAAC**(self, lamda=10, weight=0.05)  Type I Pseudo amino acid composition descriptors (default is 30)     Usage:     result = GetPAAC(lamda=10,weight=0.05)     lamda factor reflects the rank of correlation and is a non-Negative integer, such as 15.     Note that (1)lamda should NOT be larger than the length of input protein sequence;     (2) lamda must be non-Negative integer, such as 0, 1, 2, ...; (3) when lamda =0, the      output of PseAA server is the 20-D amino acid composition.     weight factor is designed for the users to put weight on the additional PseAA components      with respect to the conventional AA components. The user can select any value within the      region from 0.05 to 0.7 for the weight factor.  **GetPAACp**(self, lamda=10, weight=0.05, AAP=[])  Type I Pseudo amino acid composition descriptors for the given properties (default is 30)     Usage:     result = GetPAACp(lamda=10,weight=0.05,AAP=[])     lamda factor reflects the rank of correlation and is a non-Negative integer, such as 15.     Note that (1)lamda should NOT be larger than the length of input protein sequence;     (2) lamda must be non-Negative integer, such as 0, 1, 2, ...; (3) when lamda =0, the      output of PseAA server is the 20-D amino acid composition.     weight factor is designed for the users to put weight on the additional PseAA components      with respect to the conventional AA components. The user can select any value within the      region from 0.05 to 0.7 for the weight factor.     AAP is a list form containing the properties, each of which is a dict form.  **GetProteinSequenceFromID**(self, uniprotid='')  Downloading a protein sequence by uniprot id.  **GetQSO**(self, maxlag=30, weight=0.1)  Quasi sequence order descriptors  default is 50     result = GetQSO(maxlag=30, weight=0.1)     maxlag is the maximum lag and the length of the protein should be larger     than maxlag. default is 45.  **GetQSOp**(self, maxlag=30, weight=0.1, distancematrix={})  Quasi sequence order descriptors  default is 50     result = GetQSO(maxlag=30, weight=0.1)     maxlag is the maximum lag and the length of the protein should be larger     than maxlag. default is 45.     distancematrix is a dict form containing 400 distance values  **GetSOCN**(self, maxlag=45)  Sequence order coupling numbers  default is 45     Usage:     result = GetSOCN(maxlag=45)     maxlag is the maximum lag and the length of the protein should be larger     than maxlag. default is 45.  **GetSOCNp**(self, maxlag=45, distancematrix={})  Sequence order coupling numbers  default is 45     Usage:     result = GetSOCN(maxlag=45)     maxlag is the maximum lag and the length of the protein should be larger     than maxlag. default is 45.     distancematrix is a dict form containing 400 distance values  **GetSubSeq**(self, ToAA='S', window=3)  obtain the sub sequences wit length 2\*window+1, whose central point is ToAA     Usage:     result = GetSubSeq(ToAA='S',window=3)     ToAA is the central (query point) amino acid in the sub-sequence.     window is the span.  **GetTPComp**(self)  tri-peptide composition descriptors (8000)     Usage:     result = GetTPComp()  **GetTriad**(self)  Calculate the conjoint triad features from protein sequence.     Useage:     res = GetTriad()     Output is a dict form containing all 343 conjoint triad features.  **ReadProteinSequence**(self, ProteinSequence='')  Read a protein sequence.  **\_\_init\_\_**(self)  constructor of PyPro   ---  Data and other attributes defined here:  **AALetter** = ['A', 'R', 'N', 'D', 'C', 'E', 'Q', 'G', 'H', 'I', 'L', 'K', 'M', 'F', 'P', 'S', 'T', 'W', 'Y', 'V']  **Version** = 1.0 |
