# Supplementary material for: Protein language model-based prediction for plant miRNA encoded peptides
Source: PeerJ Comput Sci. 2025 Mar 18;11:e2733. doi: 10.7717/peerj-cs.2733 (PMC11935769; doi:10.7717/peerj-cs.2733)
Supplement: Supplemental Information 1 [file peerj-cs-11-2733-s001.zip › pLM4PEP-master/pydpi/html/topology.html]

Python: module topology

|  |  |
| --- | --- |
| **topology** | index /home/orient/pydpi/src/pydpi/drug/topology.py |

##############################################################################  
The calculation of molecular topological indices based on its topological  
   
structure. You can get 25 molecular topological descriptors. You can freely  
   
use and distribute it. If you hava  any problem, you could contact with us timely!  
   
Authors: Dongsheng Cao and Yizeng Liang.  
   
Date: 2012.09.18  
   
Email: oriental-cds@163.com  
##############################################################################

**Modules**

|  |  |  |  |
| --- | --- | --- | --- |
| rdkit.Chem  rdkit.Chem.GraphDescriptors | numpy  rdkit.Chem.rdchem | scipy |  |

|  |  |  |
| --- | --- | --- |
| **Functions** | | |
|  |  | **CalculateArithmeticTopoIndex**(mol)  #################################################################  Arithmetic topological index by Narumi     ---->Arto     Usage:             result=CalculateArithmeticTopoIndex(mol)            Input: mol is a molecule object            Output: result is a numeric value  #################################################################  **CalculateBalaban**(mol)  #################################################################  Calculation of Balaban index in a molecule     ---->J     Usage:             result=CalculateBalaban(mol)            Input: mol is a molecule object            Output: result is a numeric value  #################################################################  **CalculateBertzCT**(mol)  #################################################################  A topological index meant to quantify "complexity" of molecules.     Consists of a sum of two terms, one representing the complexity     of the bonding, the other representing the complexity of the     distribution of heteroatoms.     From S. H. Bertz, J. Am. Chem. Soc., vol 103, 3599-3601 (1981)     ---->BertzCT(log value)     Usage:             result=CalculateBertzCT(mol)            Input: mol is a molecule object            Output: result is a numeric value  #################################################################  **CalculateDiameter**(mol)  #################################################################  Calculation of diameter, which is   Largest value     in the distance matrix [Petitjean 1992].     ---->diametert     Usage:             result=CalculateDiameter(mol)            Input: mol is a molecule object            Output: result is a numeric value  #################################################################  **CalculateGeometricTopoIndex**(mol)  #################################################################  Geometric topological index by Narumi     ---->Geto     Usage:             result=CalculateGeometricTopoIndex(mol)            Input: mol is a molecule object            Output: result is a numeric value  #################################################################  **CalculateGraphDistance**(mol)  #################################################################  Calculation of graph distance index     ---->Tigdi(log value)     Usage:             result=CalculateGraphDistance(mol)            Input: mol is a molecule object            Output: result is a numeric value  #################################################################  **CalculateGutmanTopo**(mol)  #################################################################  Calculation of Gutman molecular topological index based on     simple vertex degree     ---->GMTI(log value)     Usage:             result=CalculateGutmanTopo(mol)            Input: mol is a molecule object            Output: result is a numeric value  #################################################################  **CalculateHarary**(mol)  #################################################################  Calculation of Harary number     ---->Thara     Usage:             result=CalculateHarary(mol)            Input: mol is a molecule object            Output: result is a numeric value  #################################################################  **CalculateHarmonicTopoIndex**(mol)  #################################################################  Calculation of harmonic topological index proposed by Narnumi.     ---->Hato     Usage:             result=CalculateHarmonicTopoIndex(mol)            Input: mol is a molecule object            Output: result is a numeric value  #################################################################  **CalculateIpc**(mol)  #################################################################  This returns the information content of the coefficients of the      characteristic polynomial of the adjacency matrix of a      hydrogen-suppressed graph of a molecule.     'avg = 1' returns the information content divided by the total     population.     From D. Bonchev & N. Trinajstic, J. Chem. Phys. vol 67,     4517-4533 (1977)      ---->Ipc(log value)     Usage:             result=CalculateIpc(mol)            Input: mol is a molecule object            Output: result is a numeric value  #################################################################  **CalculateMZagreb1**(mol)  #################################################################  Calculation of Modified Zagreb index with order 1 in a molecule     ---->MZM1     Usage:             result=CalculateMZagreb1(mol)            Input: mol is a molecule object            Output: result is a numeric value  #################################################################  **CalculateMZagreb2**(mol)  #################################################################  Calculation of Modified Zagreb index with order 2 in a molecule     ---->MZM2     Usage:             result=CalculateMZagreb2(mol)            Input: mol is a molecule object            Output: result is a numeric value  #################################################################  **CalculateMeanWeiner**(mol)  #################################################################  Calculation of Mean Weiner number in a molecule     ---->AW     Usage:             result=CalculateWeiner(mol)            Input: mol is a molecule object            Output: result is a numeric value  #################################################################  **CalculatePetitjean**(mol)  #################################################################  Calculation of Petitjean based on topology.     Value of (diameter - radius) / diameter as defined in [Petitjean 1992].     ---->petitjeant     Usage:             result=CalculatePetitjean(mol)            Input: mol is a molecule object            Output: result is a numeric value  #################################################################  **CalculatePlatt**(mol)  #################################################################  Calculation of Platt number in a molecule     ---->Platt     Usage:             result=CalculatePlatt(mol)            Input: mol is a molecule object            Output: result is a numeric value  #################################################################  **CalculatePoglianiIndex**(mol)  #################################################################  Calculation of Poglicani index     The Pogliani index (Dz) is the sum over all non-hydrogen atoms     of a modified vertex degree calculated as the ratio     of the number of valence electrons over the principal     quantum number of an atom [L. Pogliani, J.Phys.Chem.     1996, 100, 18065-18077].     ---->DZ     Usage:             result=CalculatePoglianiIndex(mol)            Input: mol is a molecule object            Output: result is a numeric value  #################################################################  **CalculatePolarityNumber**(mol)  #################################################################  Calculation of Polarity number.     It is the number of pairs of vertexes at     distance matrix equal to 3     ---->Pol     Usage:             result=CalculatePolarityNumber(mol)            Input: mol is a molecule object            Output: result is a numeric value  #################################################################  **CalculateQuadratic**(mol)  #################################################################  Calculation of Quadratic index in a molecule     ---->Qindex     Usage:             result=CalculateQuadratic(mol)            Input: mol is a molecule object            Output: result is a numeric value  #################################################################  **CalculateRadius**(mol)  #################################################################  Calculation of radius based on topology.     It is :If ri is the largest matrix entry in row i of the distance     matrix D,then the radius is defined as the smallest of the ri      [Petitjean 1992].     ---->radiust     Usage:             result=CalculateRadius(mol)            Input: mol is a molecule object            Output: result is a numeric value  #################################################################  **CalculateSchiultz**(mol)  #################################################################  Calculation of Schiultz number     ---->Tsch(log value)     Usage:             result=CalculateSchiultz(mol)            Input: mol is a molecule object            Output: result is a numeric value  #################################################################  **CalculateSimpleTopoIndex**(mol)  #################################################################  Calculation of the logarithm of the simple topological index by Narumi,     which is defined as the product of the vertex degree.     ---->Sito     Usage:             result=CalculateSimpleTopoIndex(mol)            Input: mol is a molecule object            Output: result is a numeric value  #################################################################  **CalculateWeiner**(mol)  #################################################################  Calculation of Weiner number in a molecule     ---->W     Usage:             result=CalculateWeiner(mol)            Input: mol is a molecule object            Output: result is a numeric value  #################################################################  **CalculateXuIndex**(mol)  #################################################################  Calculation of Xu index     ---->Xu     Usage:             result=CalculateXuIndex(mol)            Input: mol is a molecule object            Output: result is a numeric value  #################################################################  **CalculateZagreb1**(mol)  #################################################################  Calculation of Zagreb index with order 1 in a molecule     ---->ZM1     Usage:             result=CalculateZagreb1(mol)            Input: mol is a molecule object            Output: result is a numeric value  #################################################################  **CalculateZagreb2**(mol)  #################################################################  Calculation of Zagreb index with order 2 in a molecule     ---->ZM2     Usage:             result=CalculateZagreb2(mol)            Input: mol is a molecule object            Output: result is a numeric value  #################################################################  **GetTopology**(mol)  #################################################################  Get the dictionary of constitutional descriptors for given     moelcule mol     Usage:             result=CalculateWeiner(mol)            Input: mol is a molecule object            Output: result is a dict form containing all topological indices.  ################################################################# |

|  |  |  |
| --- | --- | --- |
| **Data** | | |
|  |  | **Version** = 1.0  **periodicTable** = <rdkit.Chem.rdchem.PeriodicTable object> |
