# Supplementary material for: Protein language model-based prediction for plant miRNA encoded peptides
Source: PeerJ Comput Sci. 2025 Mar 18;11:e2733. doi: 10.7717/peerj-cs.2733 (PMC11935769; doi:10.7717/peerj-cs.2733)
Supplement: Supplemental Information 1 [file peerj-cs-11-2733-s001.zip › pLM4PEP-master/pydpi/manual/Descriptors.pdf]

Molecular descriptors		
	**Constitutional descriptors**	
1[a]	Weight	Molecular weight
2	nhyd	Count of hydrogen atoms
3	nhal	Count of halogen atoms
4[a]	nhet	Count of hetero atoms
5[a]	nhev	Count of heavy atoms
6	ncof	Count of F atoms
7	ncocl	Count of Cl atoms
8	ncobr	Count of Br atoms
9	ncoi	Count of I atoms
10	ncarb	Count of C atoms
11	nphos	Count of P atoms
12	nsulph	Count of S atoms
13	noxy	Count of O atoms
14	nnitro	Count of N atoms
15[a]	nring	Number of rings
16[a]	nrot	Number of rotatable bonds
17[a]	ndonr	Number of H-bond donors
18[a]	naccr	Number of H-bond acceptors
19	nsb	Number of single bonds

20	ndb	Number of double bonds
21	ntb	Number of triple bonds
22	naro	Number of aromatic bonds
23	nta	Number of all atoms
24	AWeight	Average molecular weight
25-30	PC1<br>PC2<br>PC3<br>PC4<br>PC5<br>PC6	Molecular path counts of length 1-6
	**Topological descriptors**	
1	W	Weiner index
2	AW	Average Wiener index
3[a]	J	Balaban's J index
4	T_{hara}	Harary number
5	T_{sch}	Schiultz index
6	Tigdi	Graph distance index
7	Platt	Platt number
8	Xu	Xu index
9	Pol	Polarity number
10	Dz	Pogliani index
11[a]	Ipc	Ipc index

12[a]	BertzCT	BertzCT
13	GMTI	Gutman molecular topological index based on simple vertex degree
14-15	ZM1<br>ZM2	Zagreb index with order 1-2
16-17	MZM1<br>MZM2	Modified Zagreb index with order 1-2
18	Qindex	Quadratic index
19	diametert	Largest value in the distance matrix
20	radiust	radius based on topology
21	petitjeant	Petitjean based on topology
22	Sito	the logarithm of the simple topological index by Narumi
23	Hato	harmonic topological index proposed by Narumi
24	Geto	Geometric topological index by Narumi
25	Arto	Arithmetic topological index by Narumi
	**Connectivity descriptors**	
1-11 [a]	${}^{0}\chi^{v}$<br>${}^{1}\chi^{v}$<br>${}^{2}\chi^{v}$<br>${}^{3}\chi_{p}^{v}$<br>${}^{4}\chi_{p}^{v}$<br>${}^{5}\chi_{p}^{v}$<br>${}^{6}\chi_{p}^{v}$<br>${}^{7}\chi_{p}^{v}$<br>${}^{8}\chi_{p}^{v}$<br>${}^{9}\chi_{p}^{v}$	Valence molecular connectivity Chi index for path order 0-10

	$^{10}\chi_p^v$	
12	$^{3}\chi^v_c$	Valence molecular connectivity Chi index for three cluster
13	$^{4}\chi^v_c$	Valence molecular connectivity Chi index for four cluster
14	$^{4}\chi^v_{pc}$	Valence molecular connectivity Chi index for path/cluster
15-18	$^{3}\chi^v_{CH}$<br>$^{4}\chi^v_{CH}$<br>$^{5}\chi^v_{CH}$<br>$^{6}\chi^v_{CH}$	Valence molecular connectivity Chi index for cycles of 3-6
19-29[a]	$^{0}\chi$<br>$^{1}\chi$<br>$^{2}\chi$<br>$^{3}\chi_p$<br>$^{4}\chi_p$<br>$^{5}\chi_p$<br>$^{6}\chi_p$<br>$^{7}\chi_p$<br>$^{8}\chi_p$<br>$^{9}\chi_p$<br>$^{10}\chi_p$	Simple molecular connectivity Chi indices for path order 0-10
30	$^{3}\chi_c$	Simple molecular connectivity Chi indices for three cluster
31	$^{4}\chi_c$	Simple molecular connectivity Chi indices for four cluster
32	$^{4}\chi_{pc}$	Simple molecular connectivity Chi indices for path/cluster
33-36	$^{3}\chi_{CH}$<br>$^{4}\chi_{CH}$<br>$^{5}\chi_{CH}$<br>$^{6}\chi_{CH}$	Simple molecular connectivity Chi indices for cycles of 3-6
37	mChi1	mean chi1 (Randic) connectivity index
38	knotp	the difference between chi3c and chi4pc

39	dchi0	the difference between chi0v and chi0
40	dchi1	the difference between chi1v and chi1
41	dchi2	the difference between chi2v and chi2
42	dchi3	the difference between chi3v and chi3
43	dchi4	the difference between chi4v and chi4
44	knotpv	the difference between chiv3c and chiv4pc
	**Kappa descriptors**	
1	$^{1}\kappa_{\alpha}$	Kappa alpha index for 1 bonded fragment
2	$^{2}\kappa_{\alpha}$	Kappa alpha index for 2 bonded fragment
3	$^{3}\kappa_{\alpha}$	Kappa alpha index for 3 bonded fragment
4	phi	Kier molecular flexibility index
5[a]	$^{1}\kappa$	Molecular shape Kappa index for 1 bonded fragment
6[a]	$^{2}\kappa$	Molecular shape Kappa index for 2 bonded fragment
7[a]	$^{3}\kappa$	Molecular shape Kappa index for 3 bonded fragment
	**Burden Descriptors**	
1-16	bcutm1-16	Burden descriptors based on atomic mass
17-32	bcutv1-16	Burden descriptors based on atomic vloumes
33-48	bcute1-16	Burden descriptors based on atomic electronegativity
49-64	bcutp1-16	Burden descriptors based on polarizability
	**Basak information descriptors**	
1	IC0	Information content with order 0 proposed by Basak

2	IC1	Information content with order 1 proposed by Basak
3	IC2	Information content with order 2 proposed by Basak
4	IC3	Information content with order 3 proposed by Basak
5	IC4	Information content with order 4 proposed by Basak
6	IC5	Information content with order 5 proposed by Basak
7	IC6	Information content with order 6 proposed by Basak
8	SIC0	Complementary information content with order 0 proposed by Basak
9	SIC1	Structural information content with order 1 proposed by Basak
10	SIC2	Structural information content with order 2 proposed by Basak
11	SIC3	Structural information content with order 3 proposed by Basak
12	SIC4	Structural information content with order 4 proposed by Basak
13	SIC5	Structural information content with order 5 proposed by Basak
14	SIC6	Structural information content with order 6 proposed by Basak
15	CIC0	Complementary information content with order 0 proposed by Basak
16	CIC1	Complementary information content with order 1 proposed by Basak
17	CIC2	Complementary information content with order 2 proposed by Basak
18	CIC3	Complementary information content with order 3 proposed by Basak
19	CIC4	Complementary information content with order 4 proposed by Basak
20	CIC5	Complementary information content with order 5 proposed by Basak
21	CIC6	Complementary information content with order 6 proposed by Basak
	**E-state descriptors**	

1	S(1)	Sum of E-State of atom type: sLi
2	S(2)	Sum of E-State of atom type: ssBe
3	S(3)	Sum of E-State of atom type: ssssBe
4	S(4)	Sum of E-State of atom type: ssBH
5	S(5)	Sum of E-State of atom type: sssB
6	S(6)	Sum of E-State of atom type: ssssB
7	S(7)	Sum of E-State of atom type: sCH3
8	S(8)	Sum of E-State of atom type: dCH2
9	S(9)	Sum of E-State of atom type: ssCH2
10	S(10)	Sum of E-State of atom type: tCH
11	S(11)	Sum of E-State of atom type: dsCH
12	S(12)	Sum of E-State of atom type: aaCH
13	S(13)	Sum of E-State of atom type: sssCH
14	S(14)	Sum of E-State of atom type: ddC
15	S(15)	Sum of E-State of atom type: tsC
16	S(16)	Sum of E-State of atom type: dssC
17	S(17)	Sum of E-State of atom type: aasC
18	S(18)	Sum of E-State of atom type: aaaC
19	S(19)	Sum of E-State of atom type: ssssC
20	S(20)	Sum of E-State of atom type: sNH3
21	S(21)	Sum of E-State of atom type: sNH2

22	S(22)	Sum of E-State of atom type: ssNH2
23	S(23)	Sum of E-State of atom type: dNH
24	S(24)	Sum of E-State of atom type: ssNH
25	S(25)	Sum of E-State of atom type: aaNH
26	S(26)	Sum of E-State of atom type: tN
27	S(27)	Sum of E-State of atom type: sssNH
28	S(28)	Sum of E-State of atom type: dsN
29	S(29)	Sum of E-State of atom type: aaN
30	S(30)	Sum of E-State of atom type: sssN
31	S(31)	Sum of E-State of atom type: ddsN
32	S(32)	Sum of E-State of atom type: aasN
33	S(33)	Sum of E-State of atom type: ssssN
34	S(34)	Sum of E-State of atom type: sOH
35	S(35)	Sum of E-State of atom type: dO
36	S(36)	Sum of E-State of atom type: ssO
37	S(37)	Sum of E-State of atom type: aaO
38	S(38)	Sum of E-State of atom type: sF
39	S(39)	Sum of E-State of atom type: sSiH3
40	S(40)	Sum of E-State of atom type: ssSiH2
41	S(41)	Sum of E-State of atom type: sssSiH
42	S(42)	Sum of E-State of atom type: ssssSi

43	S(43)	Sum of E-State of atom type: sPH2
44	S(44)	Sum of E-State of atom type: ssPH
45	S(45)	Sum of E-State of atom type: sssP
46	S(46)	Sum of E-State of atom type: dsssP
47	S(47)	Sum of E-State of atom type: sssssP
48	S(48)	Sum of E-State of atom type: sSH
49	S(49)	Sum of E-State of atom type: dS
50	S(50)	Sum of E-State of atom type: ssS
51	S(51)	Sum of E-State of atom type: aaS
52	S(52)	Sum of E-State of atom type: dssS
53	S(53)	Sum of E-State of atom type: ddssS
54	S(54)	Sum of E-State of atom type: sCl
55	S(55)	Sum of E-State of atom type: sGeH3
56	S(56)	Sum of E-State of atom type: ssGeH2
57	S(57)	Sum of E-State of atom type: sssGeH
58	S(58)	Sum of E-State of atom type: ssssGe
59	S(59)	Sum of E-State of atom type: sAsH2
60	S(60)	Sum of E-State of atom type: ssAsH
61	S(61)	Sum of E-State of atom type: sssAs
62	S(62)	Sum of E-State of atom type: sssdAs
63	S(63)	Sum of E-State of atom type: sssssAs

64	S(64)	Sum of E-State of atom type: sSeH
65	S(65)	Sum of E-State of atom type: dSe
66	S(66)	Sum of E-State of atom type: ssSe
67	S(67)	Sum of E-State of atom type: aaSe
68	S(68)	Sum of E-State of atom type: dssSe
69	S(69)	Sum of E-State of atom type: ddssSe
70	S(70)	Sum of E-State of atom type: sBr
71	S(71)	Sum of E-State of atom type: sSnH3
72	S(72)	Sum of E-State of atom type: ssSnH2
73	S(73)	Sum of E-State of atom type: sssSnH
74	S(74)	Sum of E-State of atom type: ssssSn
75	S(75)	Sum of E-State of atom type: sI
76	S(76)	Sum of E-State of atom type: sPbH3
77	S(77)	Sum of E-State of atom type: ssPbH2
78	S(78)	Sum of E-State of atom type: sssPbH
79	S(79)	Sum of E-State of atom type: ssssPb
80-158	Smax1-Smax79	maxmum of E-State value of specified atom type
159-237	Smin1-Smin79	minimum of E-State value of specified atom type
	**Autocorrelation descriptors**	
1-8	ATSm1-ATSm8	Moreau-Broto autocorrelation descriptors based on atom mass
9-16	ATSv1-ATSv8	Moreau-Broto autocorrelation descriptors based on atomic van der Waals volume

17-24	ATSe1-ATSe8	Moreau-Broto autocorrelation descriptors based on atomic Sanderson electronegativity
25-32	ATSp1-ATSp8	Moreau-Broto autocorrelation descriptors based on atomic polarizability
33-40	MATSm1-MATSm8	Moran autocorrelation descriptors based on atom mass
41-48	MATSv1-MATSv8	Moran autocorrelation descriptors based on atomic van der Waals volume
49-56	MATSe1-MATSe8	Moran autocorrelation descriptors based on atomic Sanderson electronegativity
57-64	MATSp1-MATSp8	Moran autocorrelation descriptors based on atomic polarizability
65-72	GATSm1-GATSm8	Geary autocorrelation descriptors based on atom mass
73-80	GATSv1-GATSv8	Geary autocorrelation descriptors based on atomic van der Waals volume
81-88	GATSe1-GATSe8	Geary autocorrelation descriptors based on atomic Sanderson electronegativity
89-96	GATSp1-GATSp8	Geary autocorrelation descriptors based on atomic polarizability
	**Charge descriptors**	
1-4	Q_{Hmax}<br>Q_{Cmax}<br>Q_{Nmax}<br>Q_{Omax}	Most positive charge on H,C,N,O atoms
5-8	Q_{Hmin}<br>Q_{Cmin}<br>Q_{Nmin}<br>Q_{Omin}	Most negative charge on H,C,N,O atoms
9-10	Q_{max}<br>Q_{min}	Most positive and negative charge in a molecule
11-15	Q_{HSS}<br>Q_{CSS}<br>Q_{NSS}<br>Q_{OSS}	Sum of squares of charges on H,C,N,O and all toms

	Qass	
16-17	Mpc<br>Tpc	Mean and total of positive charges
18-19	Mnc<br>Tnc	Mean and total of negative charges
20-21	Mac<br>Tac	Mean and total of absolute charges
22	Rpc	Relative positive charge
23	Rnc	Relative negative charge
24	SPP	Submolecular polarity parameter
25	LDI	Local dipole index
	**Molecular property descriptors**	
1[a]	MREF	Molar refractivity
2[a]	logP	LogP value based on the Crippen method
3	logP2	Square of LogP value based on the Crippen method
4[a]	TPSA	Topological polarity surface area
5	UI	Unsaturation index
6	Hy	Hydrophilic index
	**MOE-type descriptors**	
1[a]	MTPSA	topological polar surface area based on fragments
2[a]	LabuteASA	Labute's Approximate Surface Area
3-14[a]	SLOGPVSA	MOE-type descriptors using SLogP contributions and surface area contributions
15-24[a]	SMRVSA	MOE-type descriptors using MR contributions and surface area contributions

25-38[a]	PEOEVSA	MOE-type descriptors using partial charges and surface area contributions
39-49[a]	EstateVSA	MOE-type descriptors using Estate indices and surface area contributions
50-60[a]	VSAEstate	MOE-type descriptors using surface area contributions and Estate indices
	**Fragment/Fingerprint-based descriptors**	
1[a]	FP2	(Topological fingerprint) A Daylight-like fingerprint based on hashing molecular subgraphs
2[a]	MACCS	(MACCS keys)Using the 166 public keys implemented as SMARTS
3	E-state	79 E-state fingerprints or fragments
4	FP4	307 FP4 fingerprints
5[a]	Atom Paris	Atom Paris fingerprints
6[a]	Torsions	Topological torsion fingerprints
7[a]	Morgan/Circular	Fingerprints based on the Morgan algorithm

Note: [a] indicates that these descriptors are from RDkit. In PyDPI, we wrapped most of molecular descriptors form RDkit. The other descriptors are independently coded by us.
