# Supplementary material for: Protein language model-based prediction for plant miRNA encoded peptides
Source: PeerJ Comput Sci. 2025 Mar 18;11:e2733. doi: 10.7717/peerj-cs.2733 (PMC11935769; doi:10.7717/peerj-cs.2733)
Supplement: Supplemental Information 1 [file peerj-cs-11-2733-s001.zip › pLM4PEP-master/pydpi/manual/UserGuide.pdf]

User Guide for PyDPI 1.0

Dongsheng Cao

©2012 China Computational Biology Drug Design Group

Table of Contents

1. What is this?······3
2. Install the PyDPI package······3
3. Working on drug molecules······4
   3.1. Read single molecules······4
   3.2. Download molecules from corresponding ID······5
   3.3. Calculating molecular descriptors······6
   3.4. Molecular fingerprints and chemoinforamtics······9
      3.4.1. Daylight-type fingerprints······10
      3.4.2. MACCS keys and FP4 fingerprints······10
      3.4.3. E-state fingerprints······11
      3.4.4. Atom pairs and topological torsions······11
      3.4.5. Morgan fingerprints······11
      3.4.6. Using PyDrug object······11
      3.4.7. Fingerprint similarity······12
   4. Working on protein sequences······12
   4.1. Download proteins from Uniprot······12
   4.2. Download the property from the AAindex database······14
   4.3. Calculating protein descriptors······15
5. Interaction representation······19
   5.1 Protein-protein interaction descriptors······19
   5.2. Protein-ligand interaction descriptors······19
Appendix······21

1. What is this?

This document is intended to provide an overview of how one can use the PyDPI functionality from Python. It's not comprehensive and it's not a manual.

If you find mistakes, or have suggestions for improvements, please either fix them yourselves in the source document (the .py file) or send them to the mailing list: oriental-cds@hotmail.com

2. Install the PyDPI package

PyDPI has been successfully tested on Linux and Windows systems. The author could download this package from https://sourceforge.net/projects/pydpicao/ (.zip and .tar.gz). The install process of PyDPI is very easy:

On Windows:

(1): download the pydpi package (.zip)

(2): extract or uncompress the .zip file

(3): cd pydpi-1.0

(4): python setup.py install

On Linux:

(1): download the pydpi package (.tar.gz)

(2): tar -zxf pydpi-1.0.tar.gz

(3): cd pydpi-1.0

(4): python setup.py install or sudo python setup.py install

Once the PyDPI package is installed, you can test if it is successfully installed.

```
>>> from pydpi import test
>>> test.main()
Test successfully!
>>> from pydpi import example
>>> example.main()
```

If the above functions are all correctly run, the PyDPI package is successfully installled.

Note that you must guarantee that your computer is connected into the Internet.

3. Working on drug molecules

3.1. Read single molecules

The majority of the basic drug molecular functionality is found in module pydrug:

```
>>> from pydpi import pydrug
```

Individual molecules can be constructed using a variety of approaches.

```
>>> from pydpi.pydrug import Chem
>>> mol=Chem.MolFromSmiles('O=C(Oc1ccccc1C(=O)O)C')
```

The PyDPI allow the users to provide different molecular formats.

```
>>> from pydpi import pydrug
>>> drug=pydrug.PyDrug()
>>> mol=drug.ReadMolFromSmile(smi='CC(Oc1ccccc1C(O)=O)=O')
>>> mol=drug.ReadMolFromInchi(inchi='InChI=1S/C9H8O4/c1-6(10)13-8-5-3-2-4-7(8)9(11)12/h2-5H,1H3,(H,11,12)')
>>> mol=drug.ReadMolFromMol("/home/orient/1.mol")
```

```
>>> from pydpi.pydrug import getmol
>>> mol=getmol.ReadMolFromSDF("/home/orient/drug.sdf")
>>> for i in mol:
...     print i
...
<rdkit.Chem.rdchem.Mol object at 0x5108ad0>
<rdkit.Chem.rdchem.Mol object at 0x5108a60>
<rdkit.Chem.rdchem.Mol object at 0x5108ad0>
<rdkit.Chem.rdchem.Mol object at 0x5108a60>
<rdkit.Chem.rdchem.Mol object at 0x5108ad0>
```

All of these functions return a Mol object on success:

```
>>> mol
<rdkit.Chem.rdchem.Mol object at 0x7f702c067360>
```

3.2. Download molecules from corresponding ID

The PyDPI allows the user to download the molecules by providing their IDs such as CAS, NCBI, KEGG, EBI and Drugbank.

```
>>> from pydpi.drug import getmol
>>> smi=getmol.GetMolFromCAS('50-78-2')
>>> print smi
CC(=O)Oc1ccccc1C(=O)[O-]
>>> smi=getmol.GetMolFromDrugbank('DB00945')
>>> print smi
CC(Oc1ccccc1C(O)=O)=O
>>> smi=getmol.GetMolFromKegg('D00109')
>>> print smi
CC(Oc1ccccc1C(O)=O)=O
>>> smi=getmol.GetMolFromNCBI(cid='2244')
>>> print smi
CC(Oc1ccccc1C(O)=O)=O
```

By providing a aspirin IDs, we could download its SMILES format conveniently.
We can also download a molecule by constructing a PyDrug object, which contains the majority of the basic drug molecular functionality.

```
>>> drug=pydrug.PyDrug()
>>> smi=drug.GetMolFromNCBI('2244')
>>> print smi
CC(Oc1ccccc1C(O)=O)=O
>>> mol=drug.ReadMolFromSmile(smi)
```

You could read a molecule by providing a Drugbank ID:

```
>>> drug=pydrug.PyDrug()
>>> smi=drug.GetMolFromDrugbank('DB00945')
>>> mol=drug.ReadMolFromSmile(smi)
```

3.3. Calculating molecular descriptors

The PyDPI package could calculate a large number of molecular descriptors including constitutional descriptors, topological descriptors, connectivity indices, E-state indices, autocorrelation descriptors, charge descriptors, molecular properties, kappa shape indices, MOE-type descriptors, and molecular fingerprints. These descriptors capture and magnify distinct aspects of chemical structures.

Once we read a Mol object, we could easily calculate these molecular descriptors:

Example 1: Calculating molecular constitutional descriptors

```
>>> from pydpi.drug import constitution
>>> res=constitution.CalculateMolWeight(mol)
>>> print res
172.095
>>> res=constitution.CalculatePath2(mol)
>>> print res
17
>>> res=constitution.CalculateRingNumber(mol)
>>> print res
1
>>> res=constitution.GetConstitutional(mol)
>>> print res
{'nphos': 0.0, 'ndb': 2.0, 'nsb': 5.0, 'ncoi': 0.0, 'ncarb': 9.0, 'nsulph': 4
4.0, 'nhet': 4.0, 'nhev': 13.0, 'nhal': 0.0, 'naccr': 3.0, 'nta': 21.0, 'ntb'
'PC1': 13.0, 'PC6': 19.0, 'PC4': 23.0, 'PC5': 24.0, 'AWeight': 13.238, 'ncocl
```

```
>>> from pydpi.pydrug import constitution,Chem
>>> molset=Chem.SDMolSupplier('/home/orient/drug.sdf')
>>> for mol in molset:
...     res=constitution.GetConstitutional(mol)
...     print len(res)
...
30
30
30
30
30
```

We could calculate any constitutional descriptor by calling the corresponding functions. We could also calculate all 30 descriptors by calling GetConstitutional function. The result is given in the form of dictionary.

Example 2: Calculating topology descriptors

25 topology descriptors can be calculated by the PyDPI package. For detailed information of topology descriptors, refer to Table S2 in Appendix and their introductions in Manual.

```
>>> from pydpi.drug import topology
>>> res=topology.CalculateBalaban(mol)
>>> print res
2.46175836459
>>> res=topology.CalculateSchiultz(mol)
>>> print res
981.0
>>> res=topology.GetTopology(mol)
>>> print res
{'W': 246.0, 'Geto': 1.831, 'DZ': 30.0, 'Arto': 2.0, 'radiust': 3.0, 'petitje
60.0, 'Thara': 33.783, 'Qindex': 7.0, 'Tsch': 981.0, 'J': 2.462, 'AW': 3.154,
 3.04, 'Pol': 16.0, 'Hato': 1.66, 'Xu': 12.994}
```

Example 3: Calculating molecular connectivity indices

```
>>> from pydpi.drug import connectivity
>>> from pydpi.pydrug import Chem
>>> mol=Chem.MolFromSmiles('CC(Oc1ccccc1C(O)=O)=O')
>>> print connectivity.CalculateChi2(mol)
5.58195736265
>>> print connectivity.CalculateChi3c(mol)
1.04433105395
>>> res=connectivity.GetConnectivity(mol)
>>> print res
{'Chi3ch': 0.0, 'knotp': 0.609, 'dchi3': 2.227, 'dchi2': 3.187, 'dchi1':
'Chiv1': 3.617, 'Chiv0': 6.981, 'Chiv3': 1.371, 'Chiv2': 2.395, 'Chi4c':
 'Chi8': 0.299, 'Chi9': 0.118, 'Chi2': 5.582, 'Chi3': 3.598, 'Chi0': 9.8
0.521, 'Chiv4c': 0.0, 'Chiv9': 0.01, 'Chi4pc': 1.653, 'knotpv': 0.132, '
'Chi4ch': 0.0, 'Chiv4ch': 0.0, 'mChi1': 0.47, 'Chi6ch': 0.083}
```

Example 4: Calculating molecular properties

```
>>> from pydpi.drug import molproperty
>>> from pydpi.pydrug import Chem
>>> mol=Chem.MolFromSmiles('CC(Oc1ccccc1C(O)=O)=O')
>>> print molproperty.CalculateMolLogP(mol)
1.31
>>> print molproperty.CalculateTPSA(mol)
63.6
>>> print molproperty.CalculateMolMR(mol)
44.71
>>> print molproperty.GetMolecularProperty(mol)
{'TPSA': 63.6, 'Hy': -2.562, 'LogP': 1.31, 'LogP2': 1.716, 'UI': 3.17, 'MR': 44.71}
```

Example 5: Calculating Kappa shape descriptors

```
>>> from pydpi.drug import kappa
>>> from pydpi.pydrug import Chem
>>> mol=Chem.MolFromSmiles('CC(Oc1ccccc1C(O)=O)=O')
>>> print kappa.CalculateKappa1(mol)
11.077
>>> print kappa.CalculateFlexibility(mol)
2.63909615385
>>> print kappa.CalculateKappaAlapha2(mol)
3.709
>>> res=kappa.GetKappa(mol)
>>> print res
{'phi': 2.639096153846154, 'kappa1': 11.077, 'kappa3': 3.324, 'kappa2': 5.024,
```

Example 6: Calculating charge descriptors

```
>>> from pydpi.drug import charge
>>> from pydpi.pydrug import Chem
>>> mol=Chem.MolFromSmiles('CC(Oc1ccccc1C(O)=O)=O')
>>> print charge.CalculateNSumSquareCharge(mol)
0
>>> print charge.CalculateAllMaxPCharge(mol)
0.339
>>> print charge.CalculateLocalDipoleIndex(mol)
0.322
>>> res=charge.GetCharge(mol)
>>> print res
{'QNmin': 0, 'QOss': 0.534, 'Mpc': 0.122, 'QHss': 0.108, 'SPP':
'QOmax': -0.246, 'Tpc': 1.584, 'Qmax': 0.339, 'QOmin': -0.478, '
0.214, 'Qmin': -0.478, 'Tac': 3.167, 'Mnc': -0.198}
```

Example 7: Calculating descriptors using PyDrug object

An easier way to calculate molecular descriptors is to generate a PyDrug object and then call their methods. The PyDrug contains the majority of drug molecule operation functionality.

```
>>> from pydpi.pydrug import PyDrug
>>> drug=PyDrug() #construct a PyDrug object
>>> drug.ReadMolFromSmile('CC(Oc1ccccc1C(O)=O)=O')
<rdkit.Chem.rdchem.Mol object at 0x3ef9de0>
>>> res1=drug.GetConnectivity()
>>> print len(res1)
44
>>> res2=drug.GetMOE()
>>> print len(res2)
60
>>> res3=drug.GetAllDescriptor() #calculate all descriptors
>>> print len(res3)
615
```

3.4. Molecular fingerprints and chemoinforamtics

In the PyDPI package, there are seven types of molecular fingerprints which are defined by abstracting and magnifying different aspects of molecular topology.

3.4.1. Daylight-type fingerprints

```
>>> from pydpi.drug import fingerprint
>>> from pydpi.pydrug import Chem
>>> mol=Chem.MolFromSmiles('CC(Oc1ccccc1C(O)=O)=O')
>>> res=fingerprint.CalculateDaylightFingerprint(mol)
>>> print res[0] #the number of fingerprints
2048
>>> print len(res[1]) #the number of bit 1
335
```

We can calculate the similarity between two molecules by specifying a type of similarity measure. There exist to be nine types of similarity measures to calculate the similarity between two molecules.

```
>>> print pydrug.fingerprint.similaritymeasure
['Tanimoto', 'Dice', 'Cosine', 'Sokal', 'Russel', 'Kulczynski', 'McConnaughey', 'Asymmetric', 'BraunBlanquet']
```

```
>>> from pydpi.drug import fingerprint
>>> from pydpi.pydrug import Chem
>>> ms=[Chem.MolFromSmiles(i) for i in ['CC(Oc1ccccc1C(O)=O)=O','CCCOC=O']]
>>> fps=[fingerprint.CalculateDaylightFingerprint(i) for i in ms]
>>> print fingerprint.CalculateSimilarity(fps[0][2],fps[1][2],'Tanimoto')
0.297
```

3.4.2. MACCS keys and FP4 fingerprints

```
>>> from pydpi.drug import fingerprint
>>> from pydpi.pydrug import Chem
>>> mol=Chem.MolFromSmiles('CC(Oc1ccccc1C(O)=O)=O')
>>> res1=fingerprint.CalculateMACCSFingerprint(mol)
>>> print res1[0]
166
>>> res2=fingerprint.CalculateFP4Fingerprint('CC(Oc1ccccc1C(O)=O)=O')
>>> print res2[0]
307
```

Note that the input of MACCS and FP4 is different.

3.4.3. E-state fingerprints

```
>>> from pydpi.drug import fingerprint
>>> from pydpi.pydrug import Chem
>>> mol=Chem.MolFromSmiles('CC(Oc1ccccc1C(O)=O)=O')
>>> fp=fingerprint.CalculateEstateFingerprint(mol)
>>> print fp[0]
79
>>> print fp[1]
{'12': 1, '17': 1, '16': 1, '36': 1, '35': 1, '34': 1, '7': 1}
```

3.4.4. Atom pairs and topological torsions

```
>>> from pydpi.drug import fingerprint
>>> from pydpi.pydrug import Chem
>>> mol=Chem.MolFromSmiles('CC(Oc1ccccc1C(O)=O)=O')
>>> fp1=fingerprint.CalculateAtomPairsFingerprint(mol)
>>> fp2=fingerprint.CalculateTopologicalTorsionFingerprint(mol)
>>> print fp1[0], fp2[0]
8388608 68719476735
>>> print len(fp1[1]),len(fp2[1])
51 16
```

3.4.5. Morgan fingerprints

```
>>> from pydpi.drug import fingerprint
>>> from pydpi.drug import getmol
>>> from pydpi.pydrug import Chem
>>> smi=getmol.GetMolFromNCBI('2244')
>>> mol=Chem.MolFromSmiles(smi)
>>> fp=fingerprint.CalculateMorganFingerprint(mol)
>>> print fp[0]
4294967295
>>> print len(fp[1])
25
```

3.4.6. Using PyDrug object

The convenient way to calculate the fingerprints is to generate a PyDrug object and call GetFingerprint method.

```
>>> from pydpi.pydrug import PyDrug
>>> drug=PyDrug() #generate a PyDrug object
>>> drug.ReadMolFromSmile('CC(Oc1ccccc1C(O)=O)=O')
<rdkit.Chem.rdchem.Mol object at 0x3ef9de0>
>>> from pydpi import pydrug
>>> pydrug.FingerprintName
['topological', 'Estate', 'FP4', 'atompairs', 'torsions', 'morgan', 'MACCS']
>>> FP1=drug.GetFingerprint('Estate')
>>> FP2=drug.GetFingerprint('MACCS')
>>> FP3=drug.GetFingerprint('topological')
>>> print FP1[0], FP2[0],FP3[0]
79 166 2048
```

3.4.7. Fingerprint similarity

We could any fingerprint similarity using the nine given similarity measure methods.

```
>>> print pydrug.FingerprintName
['topological', 'Estate', 'FP4', 'atompairs', 'torsions', 'morgan', 'MACCS']
>>> print pydrug.fingerprint.similaritymeasure
['Tanimoto', 'Dice', 'Cosine', 'Sokal', 'Russel', 'Kulczynski', 'McConnaughey', 'Asymmetric', 'BraunBlanquet']
>>> drug1=pydrug.PyDrug()
>>> drug1.ReadMolFromSmile('CC(Oc1ccccc1C(O)=O)=O')
<rdkit.Chem.rdchem.Mol object at 0x7f70440982f0>
>>> fp1=drug1.GetFingerprint(FPName='topological')
>>> drug2=pydrug.PyDrug()
>>> drug2.ReadMolFromSmile(smi='CCCOC=O')
<rdkit.Chem.rdchem.Mol object at 0x3fd1130>
>>> drug2.GetFingerprint(FPName='topological')
(2048, {1: 1, 34: 1, 50: 1, 36: 1, 6: 1, 7: 1, 8: 1, 42: 1, 45: 1, 13: 1, 14: 1, 15: 1, 18: 1, 20: 1, 46: 1, 2[illegible]
>>> fp2=drug2.GetFingerprint(FPName='topological')
>>> print pydrug.fingerprint.CalculateSimilarity(fp1[2], fp2[2], similarity='Tanimoto')
```

4. Working on protein sequences

4.1. Download proteins from Uniprot

```
>>> from pydpi import pypro
>>> ps=pypro.GetProteinSequence('P48039')
>>> print ps
MQGNGSALPNASQPVLRGDGARPSWLASALACVLIFTIVVDILGNLLVILSVYRNKKLRNAGNIFVVSLAVADLVVAIYPYPLVLMSIFNNGWNLGY
LHCQVSGFLMGLSVIGSIFNITGIAINRYCYICHSLKYDKLYSSKNSLCYVLLIWLLTLAAVLPNLRAGTLQYDPRIYSCTFAQSVSSAYTIAVVVF
HFLVPMIIVIFCYLRIWILVLQVRQRVKPDRKPKLKPQDFRNFVTMFVVFVLFAICWAPLNFIGLAVASDPASMVPRIPEWLFVASYYMAYFNSCLN
AIIYGLLNQNFRKEYRRIIVSLCTARVFFVDSSNDVADRVKWKPSPLMTNNNVVKVDSV
```

You can get a protein sequence from the Uniprot website by providing a Uniprot ID.

You can get the window×2+1 sub-sequences whose central point is the given amino acid ToAA.

```
>>> subseq=pypro.GetSubSequence(ps,ToAA='S',window=5)
>>> print subseq
['MQGNGSALPNA', 'ALPNASQPVLR', 'DGARPSWLASA', 'PSWLASALACV', 'LLVILSVYRNK', 'NIFVVSLAVAD',
'PLVLMSIFNNG', 'LHCQVSGFLMG', 'FLMGLSVIGSI', 'LSVIGSIFNIT', 'CYICHSLKYDK', 'YDKLYSSKNSL',
'DKLYSSKNSLC', 'YSSKNSLCYVL', 'DPRIYSCTFAQ', 'CTFAQSVSSAY', 'FAQSVSSAYTI', 'AQSVSSAYTIA',
'GLAVASDPASM', 'ASDPASMVPRI', 'WLFVASYYMAY', 'MAYFNSCLNAI', 'RRIIVSLCTAR', 'VFFVDSSNDVA',
'FFVDSSNDVAD', 'VKWKPSPLMTN']
```

You can also get several protein sequences by providing a file containing Uniprot IDs of these proteins.

```
>>> from pydpi.protein import GetProteinFromUniprot
>>> tag=GetProteinFromUniprot.GetProteinSequenceFromTxt('/home/orient/', 'target.txt', 'res.txt')
--------------------------------------------------------
The 1 protein sequence has been downloaded!
MADSCRNLTYVRGSVGPATSTLMFVAGVVGNGLALGILSARRPARPSAFAVLVTGLAATDLLGTSFLSPAVFVAYARNSSLLGLARGGPALCDAFAFA
HQQYCPGSWCFLRMRWAQPGGAAFSLAYAGLVALLVAAIFLCNGSVTLSLCRMYRQQKRHQGSLGPRPRTGEDEVDHLILLALMTVVMAVCSLPLTIR
TPLSQLASGRRDPRAPSAPVGKEGSCVPLSAWGEGQVEPLPPTQQSSGSAVGTSSKAEASVACSLC
--------------------------------------------------------
--------------------------------------------------------
The 2 protein sequence has been downloaded!
MPNNSTALSLANVTYITMEIFIGLCAIVGNVLVICVVKLNPSLQTTTFYFIVSLALADIAVGVLVMPLAIVVSLGITIHFYSCLFMTCLLLIFTHASI
VTFLSCQFVSVMRMDYMVYFSFLTWIFIPLVVMC[illegible]IYLDIFYIIRNKLSLNLSNSKETGAFYGREFKTAKSLFLVLFLFALSWLPLSIINCIIYFNGE
--------------------------------------------------------
--------------------------------------------------------
The 3 protein sequence has been downloaded!
MQGNGSALPNASQPVLRGDGARPSWLASALACVLIFTIVVDILGNLLVILSVYRNKKLRNAGNIFVVSLAVADLVVAIYPYPLVLMSIFNNGWNLGYL
PNLRAGTLQYDPRIYSCTFAQSVSSAYTIAVVVFHFLVPMIIVIFCYLRIWILVLQVRQRVKPDRKPKLKPQDFRNFVTMFVVFVLFAICWAPLNFIG
VDSSNDVADRVKWKPSPLMTNNNVVKVDSV
--------------------------------------------------------
```

The downloaded protein sequences have been saved in "/home/orient/res.txt".

The user can also download the pdb file by providing corresponding pdb id, and then extract its amino acid sequence.

```
>>> from pydpi.protein import getpdb
>>> getpdb.GetPDB(['1atp'])
Connecting...
1atp.pdb retrieved successfully.
True
>>> seq=getpdb.GetSeqFromPDB("/home/orient/1atp.pdb")
>>> print seq
GNAAAAKKGSEQESVKEFLAKAKEDFLKKWETPSQNTAQLDQFDRIKTLGTGSFGRVMLVKHKESGNHYAMKILDKQKVVKLKQI
DLIYRDLKPENLLIDQQGYIQVTDFGFAKRVKGRTWXLCGTPEYLAPEIILSKGYNKAVDWWALGVLIYEMAAGYPPFFADQPIQ
PGDTSNFDDYEEEEIRVXINEKCGKEFTEFTTYADFIASGRTGRRNAIHD
```

The downloaded protein has been saved in "/home/orient/1atp.pdb".

You could check whether the input sequence is a valid protein sequence or not.

```
>>> temp=pypro.ProteinCheck(ps)
>>> print temp
350
```

The output is the number of the protein sequence if it is valid; otherwise 0.

4.2. Download the property from the AAindex database

You could get the properties of amino acids from the AAindex database by providing a property name (e.g., KRIW790103). The output is given in the form of dictionary.

If the user provides the directory containing the AAindex database (the AAindex database could be downloaded from ftp://ftp.genome.jp/pub/db/community/aaindex/. It consists of three files: aaindex1, aaindex2 and aaindex3), the program will read the given database to get the property.

```
>>> from pydpi.pypro import GetAAIndex1, GetAAIndex23
>>> proindex=GetAAIndex1('KRIW790103',path='.')
>>> print proindex
{'A': 27.5, 'C': 44.6, 'E': 62.0, 'D': 40.0, 'G': 0.0, 'F': 115.5, 'I': 93.5, 'H': 79.0, 'K':
100.0, 'M': 94.1, 'L': 93.5, 'N': 58.7, 'Q': 80.7, 'P': 41.9, 'S': 29.3, 'R': 105.0, 'T': 51.3,
'W': 145.5, 'V': 71.5, 'Y': 117.3}
```

It should be noted that the PyDPI package has contained the AAindex database. The GetAAIndex1 methods in AAIndex will get the property from the aaindex1 database.

If the user does not provide the directory containing the AAindex database, the program will downlaod the three databases (i.e., aaindex1, aaindex2 and aaindex3) to obtain the property. It should be noted that the downloaded AAindex will be saved in the current directory. You can also specify the directory according to your needs.

```
>>> proindex=GetAAIndex23('GRAR740104',path='/home/orient/')
>>> print len(proindex)
400
```

The downloaded databases are saved in F disk. The GetAAIndex23 methods in AAIndex will get the property from the aaindex2 and aaindex3 databases.

4.3. Calculating protein descriptors

There are two ways to calculate protein descriptors in the PyDPI package. One is to directly use the corresponding methods, the other one is firstly to construct a PyPro class and then run their methods to obtain the protein descriptors. It should be noted that the output is a dictionary form, whose keys and values represent the descriptor name and the descriptor value, respectively. The user could clearly understand the meaning of each descriptor.

Use functions:

```
>>> from pydpi.pypro import AAComposition
>>> print AAComposition.CalculateAAComposition(ps)
{'A': 7.714, 'C': 2.857, 'E': 0.571, 'D': 3.429, 'G': 4.286, 'F': 5.714,
: 4.857, 'S': 7.714, 'R': 5.143, 'T': 2.571, 'W': 2.0, 'V': 11.714, 'Y':
>>> from pydpi.pypro import CTD
>>> ctd=CTD.CalculateCTD(ps)
>>> print ctd
{'_NormalizedVDWVD1075': 75.143, '_PolarityD1075': 71.714, '_SecondaryStr
0.149, '_PolarityD3025': 35.714, '_NormalizedVDWVC1': 0.306, '_Normalized
'_PolarityD1100': 100.0, '_NormalizedVDWVD2100': 100.0, '_ChargeD3050': 6
'_HydrophobicityD3050': 51.143, '_PolarityT12': 0.112, '_PolarityT13': 0.
: 0.286, '_ChargeD1075': 86.571, '_SecondaryStrD1075': 72.571, '_Polariza
74.857, '_HydrophobicityD3025': 25.143, '_PolarizabilityD3025': 33.143, '
```

We can also compute various types of descriptors based on PDB format.

```
>>> from pydpi.protein import getpdb,AAComposition
>>> getpdb.GetPDB(['1atp'])
Connecting...
1atp.pdb retrieved successfully.
True
>>> ps=getpdb.GetSeqFromPDB('/home/orient/1atp.pdb')
>>> res=AAComposition.CalculateAAComposition(ps)
>>> print len(res)
20
```

Use GetProDes class:

```
>>> from pydpi.pypro import PyPro
>>> print PyPro.AALetter
['A', 'R', 'N', 'D', 'C', 'E', 'Q', 'G', 'H', 'I', 'L', 'K', 'M', 'F', 'P', 'S', 'T', 'W', 'Y', 'V']
```

Example 1: Calculating amino acid composition descriptors

```
>>> protein=PyPro()
>>> protein=PyPro()  #generate a protein object
>>> protein.ReadProteinSequence(ps)
>>> print protein.GetAAComp()
{'A': 7.714, 'C': 2.857, 'E': 0.571, 'D': 3.429, 'G': 4.286, 'F': 5.714, 'I': 8.0,
 'H': 0.857, 'K': 3.714, 'M': 2.286, 'L': 12.286, 'N': 6.571, 'Q': 2.571, 'P':
4.857, 'S': 7.714, 'R': 5.143, 'T': 2.571, 'W': 2.0, 'V': 11.714, 'Y': 5.143}
```

Example 2: Calculating Moran autocorrelation descriptors

```
>>> protein=PyPro()  #generate a protein object
>>> protein.ReadProteinSequence(ps)
>>> moran=protein.GetMoranAuto()
>>> for i in moran:
...     print i,  moran[i]
...
MoranAuto_ResidueVol8 0.059
MoranAuto_ResidueVol9 0.042
MoranAuto_ResidueVol4 0.128
MoranAuto_ResidueVol5 0.043
MoranAuto_ResidueVol6 -0.047
MoranAuto_ResidueVol7 0.019
MoranAuto_ResidueVol1 0.049
MoranAuto_ResidueVol2 0.034
```

Example 3: Calculating pseudo amino acid composition descriptors

```
>>> protein=PyPro()  #generate a protein object
>>> protein.ReadProteinSequence(ps)
>>> paac=protein.GetPAAC(lamda=5,weight=0.05)
>>> print paac
{'PAAC24': 5.613, 'PAAC25': 5.887, 'PAAC8': 3.043, 'PAAC9': 0.609, 'PAAC2': 3.652,
 'PAAC3': 4.666, 'PAAC1': 5.478, 'PAAC6': 0.405, 'PAAC7': 1.826, 'PAAC4': 2.435,
'PAAC5': 2.029, 'PAAC21': 6.071, 'PAAC20': 8.318, 'PAAC23': 5.897, 'PAAC22': 5.521
, 'PAAC18': 1.42, 'PAAC19': 3.652, 'PAAC14': 4.058, 'PAAC15': 3.449, 'PAAC16':
5.478, 'PAAC17': 1.826, 'PAAC10': 5.681, 'PAAC11': 8.724, 'PAAC12': 2.637,
'PAAC13': 1.623}
```

When we change the values of lamda and weight, we could get different PAAC values. Note that the number of PAAC depends on the choice of lamda. If lamda = 10, we can obtain 20+lamda=30 PAAC descriptors.

```
>>> paac=protein.GetPAAC(lamda=10,weight=0.05)
>>> print paac
{'PAAC29': 4.548, 'PAAC24': 4.283, 'PAAC28': 4.787, 'PAAC25': 4.491, 'PAAC23': 4.5
, 'PAAC8': 2.322, 'PAAC9': 0.464, 'PAAC2': 2.786, 'PAAC3': 3.56, 'PAAC1': 4.179,
'PAAC6': 0.309, 'PAAC7': 1.393, 'PAAC4': 1.858, 'PAAC5': 1.548, 'PAAC21': 4.632,
'PAAC20': 6.346, 'PAAC30': 4.863, 'PAAC22': 4.212, 'PAAC18': 1.084, 'PAAC19':
2.786, 'PAAC27': 4.681, 'PAAC26': 4.825, 'PAAC14': 3.096, 'PAAC15': 2.631,
'PAAC16': 4.179, 'PAAC17': 1.393, 'PAAC10': 4.334, 'PAAC11': 6.656, 'PAAC12':
2.012, 'PAAC13': 1.239}
```

Example 4: Calculating all protein descriptors

The PyPro class includes a built-in method which can calculate all protein descriptors.

```
>>> allp=protein.GetALL()
>>> print len(allp)
2049
```

Example 5: Calculating protein descriptors based on the user-defined property

The user could provide some property in the form of dictionary in python. Thus, PyDPI could calculate the descriptors based on the user-defined property.

```
>>> from pydpi.pypro import PseudoAAC
>>> Hy=PseudoAAC._Hydrophobicity
>>> pk1=PseudoAAC._pK1
>>> rm=PseudoAAC._residuemass
>>> print rm
{'A': 15.0, 'C': 47.0, 'E': 73.0, 'D': 59.0, 'G': 1.0, 'F': 91.0, 'I': 57.0, 'H': 82.0
31.0, 'R': 101.0, 'T': 45.0, 'W': 130.0, 'V': 43.0, 'Y': 107.0}
>>> from pydpi.pypro import PyPro
>>> protein=PyPro()#generate a protein object
>>> protein.ReadProteinSequence(ps)
>>> paacp=protein.GetPAACp(lamda=10,weight=0.05,AAP=[Hy,pk1,rm])
>>> print paacp
{'PAAC29': 4.475, 'PAAC24': 4.285, 'PAAC28': 4.679, 'PAAC25': 4.611, 'PAAC23': 4.518,
4.187, 'PAAC6': 0.31, 'PAAC7': 1.395, 'PAAC4': 1.861, 'PAAC5': 1.551, 'PAAC21': 4.532,
'PAAC19': 2.791, 'PAAC27': 4.66, 'PAAC26': 4.798, 'PAAC14': 3.101, 'PAAC15': 2.636, 'P
'PAAC12': 2.016, 'PAAC13': 1.241}
```

Example 6: Calculating protein descriptors based on the property from AAindex

A powerful ability of PyDPI is that it can easily calculate thousands of protein features through automatically obtaining the needed property from AAindex.

```
>>> from pydpi.pypro import GetAAIndex1, GetAAIndex23
>>> proindex=GetAAIndex1('KRIW790103',path='.')
>>> print proindex
{'A': 27.5, 'C': 44.6, 'E': 62.0, 'D': 40.0, 'G': 0.0, 'F': 115.5, 'I': 93.5,
: 29.3, 'R': 105.0, 'T': 51.3, 'W': 145.5, 'V': 71.5, 'Y': 117.3}
>>> from pydpi.pypro import PyPro
>>> protein=PyPro()#generate a protein object
>>> protein.ReadProteinSequence(ps)
>>> gearyp=protein.GetGearyAutop(AAP=proindex,AAPName='p')
>>> print len(gearyp)
30
```

5. Interaction representation

5.1 Protein-protein interaction descriptors

```
>>> from pydpi import pydpi
>>> ppi=pydpi.PyPPI()
>>> ps1=ppi.GetProteinSequenceFromID('P48039') #download protein sequence
>>> ppi.ReadProteinSequence(ps1)
>>> pdict1=ppi.GetPAAC(lamda=10, weight=0.05)
>>> ps2=ppi.GetProteinSequenceFromID('P33765') #download protein sequence
>>> ppi.ReadProteinSequence(ps2)
>>> pdict2=ppi.GetPAAC(lamda=10, weight=0.05)
>>> print ppi.GetPPIFeature1(pdict1,pdict2)
```

5.2. Protein-ligand interaction descriptors

```
>>> from pydpi import pydpi
>>> dpi=pydpi.PyDPI()  #construct a dpi object
>>> ps=dpi.GetProteinSequenceFromID('P48039') #download a protein sequence from uniprot
>>> dpi.ReadProteinSequence(ps)  #read a protein sequence
>>> pdict=dpi.GetAAComp() #calculate amino acid composition descriptors
>>> smi=dpi.GetMolFromNCBI('2244') #download a small molecule from NCBI
>>> print smi
CC(Oc1ccccc1C(O)=O)=O
>>> dpi.ReadMolFromSmile(smi) #read a molecule
<rdkit.Chem.rdchem.Mol object at 0x3601130>
>>> ddict=dpi.GetKappa() #calculate kappa descriptors
>>> f1=dpi.GetDPIFeature1(ddict,pdict) #calculate protein-ligand interaction features
>>> print f1
{'A': 7.714, 'phi': 2.639096153846154, 'E': 0.571, 'D': 3.429, 'G': 4.286, 'F': 5.714, 'I':
'L': 12.286, 'Q': 2.571, 'P': 4.857, 'S': 7.714, 'R': 5.143, 'kappa1': 11.077, 'T': 2.571, '
 9.25, 'kappam3': 2.297, 'kappam2': 3.709, 'W': 2.0}
>>> f2=dpi.GetDPIFeature2(ddict,pdict) #calculate protein-ligand interaction features
>>> print len(f2)
140
```

Appendix:

Table S1 List of propy computed features for protein sequences

Feature group	Features	Number of descriptors
Amino acid composition	Amino acid composition	20
	Dipeptide composition	400
	Tripeptide composition	8000
Autocorrelation	Normalized Moreau-Broto autocorrelation	240[a]
	Moran autocorrelation	240 [a]
	Geary autocorrelation	240 [a]
CTD	Composition	21
	Transition	21
	Distribution	105
Conjoint triad	Conjoint triad features	343
Quasi-sequence order	Sequence order coupling number	60
	Quasi-sequence order descriptors	100
Pseudo amino acid composition	Pseudo amino acid composition	50 [b]
	Amphiphilic pseudo amino acid composition	50[c]

[a] The number depends on the choice of the number of properties of amino acid and the choice of the maximum values of the lag. The default is use eight types of properties and lag = 30.

[b] The number depends on the choice of the number of the set of amino acid properties and the choice of the lamda value. The default is use three types of properties proposed by Chou et al and lamda = 30.

[c] The number depends on the choice of the lamda vlaue. The default is that lamda = 30.

Table S2 List of PyDPI computed descriptors for small molecules

Molecular descriptors		
	**Constitutional descriptors**	
1[a]	Weight	Molecular weight
2	nhyd	Count of hydrogen atoms
3	nhal	Count of halogen atoms
4[a]	nhet	Count of hetero atoms
5[a]	nhev	Count of heavy atoms
6	ncof	Count of F atoms
7	ncocl	Count of Cl atoms
8	ncobr	Count of Br atoms
9	ncoi	Count of I atoms
10	ncarb	Count of C atoms
11	nphos	Count of P atoms
12	nsulph	Count of S atoms
13	noxy	Count of O atoms
14	nnitro	Count of N atoms
15[a]	nring	Number of rings
16[a]	nrot	Number of rotatable bonds
17[a]	ndonr	Number of H-bond donors

18[a]	naccr	Number of H-bond acceptors
19	nsb	Number of single bonds
20	ndb	Number of double bonds
21	ntb	Number of triple bonds
22	naro	Number of aromatic bonds
23	nta	Number of all atoms
24	AWeight	Average molecular weight
25-30	PC1<br>PC2<br>PC3<br>PC4<br>PC5<br>PC6	Molecular path counts of length 1-6
	**Topological descriptors**	
1	W	Weiner index
2	AW	Average Wiener index
3[a]	J	Balaban's J index
4	T_{hara}	Harary number
5	T_{sch}	Schiultz index
6	Tigdi	Graph distance index
7	Platt	Platt number
8	Xu	Xu index

9	Pol	Polarity number
10	Dz	Pogliani index
11[a]	Ipc	Ipc index
12[a]	BertzCT	BertzCT
13	GMTI	Gutman molecular topological index based on simple vertex degree
14-15	ZM1<br>ZM2	Zagreb index with order 1-2
16-17	MZM1<br>MZM2	Modified Zagreb index with order 1-2
18	Qindex	Quadratic index
19	diametert	Largest value in the distance matrix
20	radiust	radius based on topology
21	petitjeant	Petitjean based on topology
22	Sito	the logarithm of the simple topological index by Narumi
23	Hato	harmonic topological index proposed by Narumi
24	Geto	Geometric topological index by Narumi
25	Arto	Arithmetic topological index by Narumi
	**Connectivity descriptors**	
1-11 [a]	$^{0}\chi^{v}$<br>$^{1}\chi^{v}$<br>$^{2}\chi^{v}$<br>$^{3}\chi_{p}^{v}$	Valence molecular connectivity Chi index for path order 0-10

	$^{4}\chi_{p}^{v}$<br>$^{5}\chi_{p}^{v}$<br>$^{6}\chi_{p}^{v}$<br>$^{7}\chi_{p}^{v}$<br>$^{8}\chi_{p}^{v}$<br>$^{9}\chi_{p}^{v}$<br>$^{10}\chi_{p}^{v}$	
12	$^{3}\chi^{v}_{c}$	Valence molecular connectivity Chi index for three cluster
13	$^{4}\chi^{v}_{c}$	Valence molecular connectivity Chi index for four cluster
14	$^{4}\chi^{v}_{pc}$	Valence molecular connectivity Chi index for path/cluster
15-18	$^{3}\chi^{v}_{CH}$<br>$^{4}\chi^{v}_{CH}$<br>$^{5}\chi^{v}_{CH}$<br>$^{6}\chi^{v}_{CH}$	Valence molecular connectivity Chi index for cycles of 3-6
19-29[a]	$^{0}\chi$<br>$^{1}\chi$<br>$^{2}\chi$<br>$^{3}\chi_{p}$<br>$^{4}\chi_{p}$<br>$^{5}\chi_{p}$<br>$^{6}\chi_{p}$<br>$^{7}\chi_{p}$<br>$^{8}\chi_{p}$<br>$^{9}\chi_{p}$<br>$^{10}\chi_{p}$	Simple molecular connectivity Chi indices for path order 0-10
30	$^{3}\chi_{c}$	Simple molecular connectivity Chi indices for three cluster
31	$^{4}\chi_{c}$	Simple molecular connectivity Chi indices for four cluster
32	$^{4}\chi_{pc}$	Simple molecular connectivity Chi indices for path/cluster

33-36	$^{3}\chi_{CH}$<br>$^{4}\chi_{CH}$<br>$^{5}\chi_{CH}$<br>$^{6}\chi_{CH}$	Simple molecular connectivity Chi indices for cycles of 3-6
37	mChi1	mean chi1 (Randic) connectivity index
38	knotp	the difference between chi3c and chi4pc
39	dchi0	the difference between chi0v and chi0
40	dchi1	the difference between chi1v and chi1
41	dchi2	the difference between chi2v and chi2
42	dchi3	the difference between chi3v and chi3
43	dchi4	the difference between chi4v and chi4
44	knotpv	the difference between chiv3c and chiv4pc
	**Kappa descriptors**	
1	$^{1}\kappa_{\alpha}$	Kappa alpha index for 1 bonded fragment
2	$^{2}\kappa_{\alpha}$	Kappa alpha index for 2 bonded fragment
3	$^{3}\kappa_{\alpha}$	Kappa alpha index for 3 bonded fragment
4	phi	Kier molecular flexibility index
5[a]	$^{1}\kappa$	Molecular shape Kappa index for 1 bonded fragment
6[a]	$^{2}\kappa$	Molecular shape Kappa index for 2 bonded fragment
7[a]	$^{3}\kappa$	Molecular shape Kappa index for 3 bonded fragment
	**Burden Descriptors**	

1-16	bcutm1-16	Burden descriptors based on atomic mass
17-32	bcutv1-16	Burden descriptors based on atomic vloumes
33-48	bcute1-16	Burden descriptors based on atomic electronegativity
49-64	bcutp1-16	Burden descriptors based on polarizability
	**Basak information descriptors**	
1	IC0	Information content with order 0 proposed by Basak
2	IC1	Information content with order 1 proposed by Basak
3	IC2	Information content with order 2 proposed by Basak
4	IC3	Information content with order 3 proposed by Basak
5	IC4	Information content with order 4 proposed by Basak
6	IC5	Information content with order 5 proposed by Basak
7	IC6	Information content with order 6 proposed by Basak
8	SIC0	Complementary information content with order 0 proposed by Basak
9	SIC1	Structural information content with order 1 proposed by Basak
10	SIC2	Structural information content with order 2 proposed by Basak
11	SIC3	Structural information content with order 3 proposed by Basak
12	SIC4	Structural information content with order 4 proposed by Basak
13	SIC5	Structural information content with order 5 proposed by Basak
14	SIC6	Structural information content with order 6 proposed by Basak
15	CIC0	Complementary information content with order 0

		proposed by Basak
16	CIC1	Complementary information content with order 1 proposed by Basak
17	CIC2	Complementary information content with order 2 proposed by Basak
18	CIC3	Complementary information content with order 3 proposed by Basak
19	CIC4	Complementary information content with order 4 proposed by Basak
20	CIC5	Complementary information content with order 5 proposed by Basak
21	CIC6	Complementary information content with order 6 proposed by Basak
	**E-state descriptors**	
1	S(1)	Sum of E-State of atom type: sLi
2	S(2)	Sum of E-State of atom type: ssBe
3	S(3)	Sum of E-State of atom type: ssssBe
4	S(4)	Sum of E-State of atom type: ssBH
5	S(5)	Sum of E-State of atom type: sssB
6	S(6)	Sum of E-State of atom type: ssssB
7	S(7)	Sum of E-State of atom type: sCH3
8	S(8)	Sum of E-State of atom type: dCH2
9	S(9)	Sum of E-State of atom type: ssCH2
10	S(10)	Sum of E-State of atom type: tCH
11	S(11)	Sum of E-State of atom type: dsCH
12	S(12)	Sum of E-State of atom type: aaCH

13	S(13)	Sum of E-State of atom type: sssCH
14	S(14)	Sum of E-State of atom type: ddC
15	S(15)	Sum of E-State of atom type: tsC
16	S(16)	Sum of E-State of atom type: dssC
17	S(17)	Sum of E-State of atom type: aasC
18	S(18)	Sum of E-State of atom type: aaaC
19	S(19)	Sum of E-State of atom type: ssssC
20	S(20)	Sum of E-State of atom type: sNH3
21	S(21)	Sum of E-State of atom type: sNH2
22	S(22)	Sum of E-State of atom type: ssNH2
23	S(23)	Sum of E-State of atom type: dNH
24	S(24)	Sum of E-State of atom type: ssNH
25	S(25)	Sum of E-State of atom type: aaNH
26	S(26)	Sum of E-State of atom type: tN
27	S(27)	Sum of E-State of atom type: sssNH
28	S(28)	Sum of E-State of atom type: dsN
29	S(29)	Sum of E-State of atom type: aaN
30	S(30)	Sum of E-State of atom type: sssN
31	S(31)	Sum of E-State of atom type: ddsN
32	S(32)	Sum of E-State of atom type: aasN

33	S(33)	Sum of E-State of atom type: ssssN
34	S(34)	Sum of E-State of atom type: sOH
35	S(35)	Sum of E-State of atom type: dO
36	S(36)	Sum of E-State of atom type: ssO
37	S(37)	Sum of E-State of atom type: aaO
38	S(38)	Sum of E-State of atom type: sF
39	S(39)	Sum of E-State of atom type: sSiH3
40	S(40)	Sum of E-State of atom type: ssSiH2
41	S(41)	Sum of E-State of atom type: sssSiH
42	S(42)	Sum of E-State of atom type: ssssSi
43	S(43)	Sum of E-State of atom type: sPH2
44	S(44)	Sum of E-State of atom type: ssPH
45	S(45)	Sum of E-State of atom type: sssP
46	S(46)	Sum of E-State of atom type: dsssP
47	S(47)	Sum of E-State of atom type: sssssP
48	S(48)	Sum of E-State of atom type: sSH
49	S(49)	Sum of E-State of atom type: dS
50	S(50)	Sum of E-State of atom type: ssS
51	S(51)	Sum of E-State of atom type: aaS
52	S(52)	Sum of E-State of atom type: dssS

53	S(53)	Sum of E-State of atom type: ddssS
54	S(54)	Sum of E-State of atom type: sCl
55	S(55)	Sum of E-State of atom type: sGeH3
56	S(56)	Sum of E-State of atom type: ssGeH2
57	S(57)	Sum of E-State of atom type: sssGeH
58	S(58)	Sum of E-State of atom type: ssssGe
59	S(59)	Sum of E-State of atom type: sAsH2
60	S(60)	Sum of E-State of atom type: ssAsH
61	S(61)	Sum of E-State of atom type: sssAs
62	S(62)	Sum of E-State of atom type: sssdAs
63	S(63)	Sum of E-State of atom type: sssssAs
64	S(64)	Sum of E-State of atom type: sSeH
65	S(65)	Sum of E-State of atom type: dSe
66	S(66)	Sum of E-State of atom type: ssSe
67	S(67)	Sum of E-State of atom type: aaSe
68	S(68)	Sum of E-State of atom type: dssSe
69	S(69)	Sum of E-State of atom type: ddssSe
70	S(70)	Sum of E-State of atom type: sBr
71	S(71)	Sum of E-State of atom type: sSnH3
72	S(72)	Sum of E-State of atom type: ssSnH2

73	S(73)	Sum of E-State of atom type: sssSnH
74	S(74)	Sum of E-State of atom type: ssssSn
75	S(75)	Sum of E-State of atom type: sI
76	S(76)	Sum of E-State of atom type: sPbH3
77	S(77)	Sum of E-State of atom type: ssPbH2
78	S(78)	Sum of E-State of atom type: sssPbH
79	S(79)	Sum of E-State of atom type: ssssPb
80-158	Smax1-Smax79	maxmum of E-State value of specified atom type
159-237	Smin1-Smin79	minimum of E-State value of specified atom type
	**Autocorrelation descriptors**	
1-8	ATSm1-ATSm8	Moreau-Broto autocorrelation descriptors based on atom mass
9-16	ATSv1-ATSv8	Moreau-Broto autocorrelation descriptors based on atomic van der Waals volume
17-24	ATSe1-ATSe8	Moreau-Broto autocorrelation descriptors based on atomic Sanderson electronegativity
25-32	ATSp1-ATSp8	Moreau-Broto autocorrelation descriptors based on atomic polarizability
33-40	MATSm1-MATSm8	Moran autocorrelation descriptors based on atom mass
41-48	MATSv1-MATSv8	Moran autocorrelation descriptors based on atomic van der Waals volume
49-56	MATSe1-MATSe8	Moran autocorrelation descriptors based on atomic Sanderson electronegativity
57-64	MATSp1-MATSp8	Moran autocorrelation descriptors based on atomic polarizability

65-72	GATSm1-GATSm8	Geary autocorrelation descriptors based on atom mass
73-80	GATSv1-GATSv8	Geary autocorrelation descriptors based on atomic van der Waals volume
81-88	GATSe1-GATSe8	Geary autocorrelation descriptors based on atomic Sanderson electronegativity
89-96	GATSp1-GATSp8	Geary autocorrelation descriptors based on atomic polarizability
	**Charge descriptors**	
1-4	Q_{Hmax}<br>Q_{Cmax}<br>Q_{Nmax}<br>Q_{Omax}	Most positive charge on H,C,N,O atoms
5-8	Q_{Hmin}<br>Q_{Cmin}<br>Q_{Nmin}<br>Q_{Omin}	Most negative charge on H,C,N,O atoms
9-10	Q_{max}<br>Q_{min}	Most positive and negative charge in a molecule
11-15	Q_{HSS}<br>Q_{CSS}<br>Q_{NSS}<br>Q_{OSS}<br>Qass	Sum of squares of charges on H,C,N,O and all toms
16-17	Mpc<br>Tpc	Mean and total of positive charges
18-19	Mnc<br>Tnc	Mean and total of negative charges
20-21	Mac<br>Tac	Mean and total of absolute charges
22	Rpc	Relative positive charge

23	Rnc	Relative negative charge
24	SPP	Submolecular polarity parameter
25	LDI	Local dipole index
	**Molecular property descriptors**	
1[a]	MREF	Molar refractivity
2[a]	logP	LogP value based on the Crippen method
3	logP2	Square of LogP value based on the Crippen method
4[a]	TPSA	Topological polarity surface area
5	UI	Unsaturation index
6	Hy	Hydrophilic index
	**MOE-type descriptors**	
1[a]	MTPSA	topological polar surface area based on fragments
2[a]	LabuteASA	Labute's Approximate Surface Area
3-14[a]	SLOGPVSA	MOE-type descriptors using SLogP contributions and surface area contributions
15-24[a]	SMRVSA	MOE-type descriptors using MR contributions and surface area contributions
25-38[a]	PEOEVSA	MOE-type descriptors using partial charges and surface area contributions
39-49[a]	EstateVSA	MOE-type descriptors using Estate indices and surface area contributions
50-60[a]	VSAEstate	MOE-type descriptors using surface area contributions and Estate indices

	**Fragment/Fingerprint-based descriptors**	
1[a]	FP2	(Topological fingerprint) A Daylight-like fingerprint based on hashing molecular subgraphs
2[a]	MACCS	(MACCS keys)Using the 166 public keys implemented as SMARTS
3	E-state	79 E-state fingerprints or fragments
4	FP4	307 FP4 fingerprints
5[a]	Atom Paris	Atom Paris fingerprints
6[a]	Torsions	Topological torsion fingerprints
7[a]	Morgan/Circular	Fingerprints based on the Morgan algorithm

Note: [a] indicates that these descriptors are from RDkit. In PyDPI, we wrapped most of molecular descriptors form RDkit. The other descriptors are independently coded by us.
