# Supplementary material for: Protein language model-based prediction for plant miRNA encoded peptides
Source: PeerJ Comput Sci. 2025 Mar 18;11:e2733. doi: 10.7717/peerj-cs.2733 (PMC11935769; doi:10.7717/peerj-cs.2733)
Supplement: Supplemental Information 1 [file peerj-cs-11-2733-s001.zip › pLM4PEP-master/pydpi/manual/manual.pdf]

Molecular Descriptors Guide

Description of the Molecular Descriptors Appearing in the PyDPI Software Package

Version1.0

©2012 China Computational Biology Drug Design Group

Table of Contents

1 Descriptors of drugs....4
1.1 Molecular constitutional descriptors....4
1.2 Topological descriptors....6
1.3 Molecular connectivity indices....11
1.4 Kappa shape descriptors....13
1.5 Burden descriptors....15
1.6 Basak descriptors....19
1.7 Electrotopological State Indices....19
1.8 Autocorrelation descriptors....23
1.8.1 Moreau-Broto autocorrelation descriptors....24
1.8.2 Moran autocorrelation descriptors....26
1.8.3 Geary autocorrelation descriptors....27
1.9 Charge descriptors....28
1.10 molecular properties....30
1.11 MOE-type descriptors....31
1.12 Molecular fingerprint....33
1.12.1 Daylight-type fingerprint....35
1.12.2 MACCS keys and FP4 fingerprint....35
1.12.3 E-state fingerprint....35
1.12.4 Atom pairs and topological torsions fingerprints....35
1.12.5 Morgan fingerprint....36
References:....37
2 Descriptors of proteins and peptides....39
2.1 Amino acid composition....39
2.2 Dipeptide composition....39
2.3 Tripeptide composition....39
2.4 Autocorrelation descriptors....40
2.4.1 Normalized Moreau-Broto autocorrelation descriptors....40
2.4.2 Moran autocorrelation....41
2.4.3 Geary autocorrelation Descriptors....41
2.5 Composition, transition and distribution....42
2.6 Conjoint Triad Descriptors....44
2.7 Quasi-sequence-order Descriptors....46
2.7.1 Sequence-order-coupling numbers....47
2.7.2 Quasi-sequence-order (QSO) descriptors....47
2.8 pseudo-amino acid composition (PAAC)....48
2.9 Amphiphilic pseudo-amino acid composition (APAAC)....50
References:....53
3 Protein-protein interaction descriptors....54
4 Protein-ligand interaction descriptors....55

References:..56
Appendix:..57

1 Descriptors of drugs

A small or drug molecule could be represented by its chemical structure. In the PyDPI software, we calculate twelve types of molecular descriptors to represent drug molecules, including constitutional descriptors, topological descriptors, connectivity indices, Burden descriptors, basak's information indices, E-state indices, autocorrelation descriptors, charge descriptors, molecular properties, kappa shape indices, MOE-type descriptors, and molecular fingerprints. These descriptors capture and magnify distinct aspects of chemical structures.

1.1 Molecular constitutional descriptors

1. Molecular weight (*Weight*)
2. Count of hydrogen atoms (*nhyd*)
3. Count of halogen atoms (*nhal*)
4. Count of hetero atoms (*nhet*)
5. Count of heavy atoms (*nhev*)
6. Count of F atoms (*ncof*)
7. Count of Cl atoms (*ncocl*)
8. Count of Br atoms (*ncobr*)
9. Count of I atoms (*ncoi*)
10. Count of C atoms (*ncarb*)
11. Count of P atoms (*nphos*)
12. Count of S atoms (*nsulph*)
13. Count of O atoms (*noxy*)
14. Count of N atoms (*nnitro*)
15. Number of rings (*nring*)
16. Number of rotatable bonds (*nrot*)
17. Number of H-bond donors (*ndonr*)
18. Number of H-bond acceptors (*naccr*)
19. Number of single bonds (*nsb*)
20. Number of double bonds (*ndb*)

21. Number of triple bonds (*ntb*)
22. Number of aromatic bonds (*naro*)
23. Number of all atoms (*nta*)
24. Average molecular weight (*AWeight*)
25. Molecular path counts of length 1 (*PC1*)
26. Molecular path counts of length 2 (*PC2*)
27. Molecular path counts of length 3 (*PC3*)
28. Molecular path counts of length 4 (*PC4*)
29. Molecular path counts of length 5 (*PC5*)
30. Molecular path counts of length 6 (*PC6*)

Introduction:

(1) The molecular weight (MW) is the sum of molecular weights of the individual atoms, defined as:

$$MW = \sum_{i=1}^{A} MW_i$$

and the average molecular weight (AWeight) is given as follows:

AWeight=MW/nAT

where nAT is the number of atoms

(2) The number of hydrogen (*nhyd*), carbon (*ncarb*), nitrogen (*nnitro*), oxygen (*noxy*), phosphorus (*nphos*), sulfur (*nsulph*), fluorine (*ncof*), chlorine (*ncocl*), bromine (*ncobr*), and iodine (*ncoi*) atoms are simply the total number of each of these types of atoms in the molecule.

The number of halogen atoms (*nhal*) is simply the sum of the counts of the halogen atoms; the number of heavy atoms (*nhev*) and hetero atoms (*nhet*) are defined the similar way.

(3) From descriptor 15 to 22, they are simply the number of ring, single bond, double bond, aromatic bond and H-acceptor, etc, in the molecule.

(4) From descriptor 25 to 30, they represent the number of path of length 1-6. The path of length *n* indicates the shortest distance equal *n* between two atoms in a topological molecular graph.

1.2 Topological descriptors

1. Weiner index (*W*)
2. Average Weiner index (*AW*)
3. Balaban's J index (*J*)
4. Harary number (T_{hara})
5. Schiultz index (T_{sch})
6. Graph distance index (*Tigdi*)
7. Platt number (*Platt*)
8. Xu index (*Xu*)
9. Polarity number (*Pol*)
10. Pogliani index (*Dz*)
11. Ipc index (*Ipc*)
12. BertzCT (*BertzCT*)
13. Gutman molecular topological index based on simple vertex degree (*GMTI*)
14. Zagreb index with order 1 (*ZM1*)
15. Zagreb index with order 2 (*ZM2*)
16. Modified Zagreb index with order 1 (*MZM1*)
17. Modified Zagreb index with order 2 (*MZM2*)
18. Quadratic index (*Qindex*)
19. Largest value in the distance matrix (*diametert*)
20. Radius based on topology (*radiust*)
21. Petitjean based on topology (*petitjeant*)
22. The logarithm of the simple topological index by Narumi (*Sito*)
23. Harmonic topological index proposed by Narnumi (*Hato*)
24. Geometric topological index by Narumi (*Geto*)
25. Arithmetic topological index by Narumi (*Arto*)

Introduction:

(1) Weiner index (*W*)

$$W = (\sum d_{ij}) / 2$$

d_{ij} is the entries of distance matrix D from H-depleted molecular graph.

(2) Average Weiner index (*AW*)

The average Weiner index is given by

$$WA = \frac{2W}{A(A-1)}$$

where A is the total number of atoms in the molecule, W and AW are described in more detail on pa 497 of the Handbook of Molecular Descriptors

(3) Balaban's J index (*J*)

$$J = \frac{B}{C+1}\sum_b (\sigma_i \sigma_j)_b^{-1/2}$$

where σ_i and σ_j are the vertex distance degree of adjacent atoms, and the sum run over all the molecular bond b, B is the number of bonds in the molecular graph and C is the number of rings. *J* are described in more detail on pa 21 of the Handbook of Molecular Descriptors

(4) Harary number (T_{hara})

$$H = \frac{1}{2}\sum_i \sum_j d_{ij}^{-1}$$

The Harary index is a molecular topological index derived from the reciprocal distance matrix D^{-1}

(5) Schiultz index (T_{sch})

$$MTI = \sum_{i=1}^{n} [(\mathbf{A} + \mathbf{D})v]_i$$

It is a topological index derived from the adjacency matrix **A**, the distance matrix **D** and *n*-dimensional column vector ***v*** constituted by the vertex degree of the A atoms.

(6) Graph distance index (*Tigdi*)

The graph distance index is defined as the squared sum of all graph distance counts:

$$GDI = \sum_{k=1}^{D} ({}^{k}f)^2$$

where D is the topological diameter, ${}^{k}f$ is the total number of distances in the graph equal to k.

(7) Platt number (*Platt*)

Platt number is also known as the total edge adjacency index A_E, it is the sum over all entries of the edge adjacency matrix:

$$A_E = \sum_{i=1}^{B} \sum_{j=1}^{B} E_{ij}$$

where B is the number of edges in molecular graph

(8) Xu index (*Xu*)

It is a topological molecular descriptor based on the adjacency matrix and distance matrix; it is defined as:

$$Xu = \sqrt{A} \log \frac{\sum_{i=1}^{A} \delta_i \sigma_i^2}{\sum_{i=1}^{A} \delta_i \sigma_i}$$

where A is the number of atoms, δ is vertex degree and σ is distance degree of all the atoms.

(9) Polarity number (*Pol*)

It is usually assumed that the polarity number accounts for the flexibility of acyclic structure; it is usually calculated on the distance matrix as the number of pairs of vertices at a topological distance equal to three. Some other polarity number also been defined based on different rules.

(10) Pogliani index (*Dz*)

$$D^Z = \sum_{i=1}^{A} \frac{Z_i^v}{L_i}$$

where A is the number of atoms, Z is the number of valence electrons and L the principal quantum number.

(11) Ipc index (*Ipc*)

Ipc index is the information for polynomial coefficients based information theory.

(12) BertzCT (*BertzCT*)

It is the most popular complexity index, taking into account both the variety of kinds of bond connectivities and atom types. It is defined as:

$$I_{CPX} = I_{CPB} + I_{CPA}$$

where I_{CPB} and I_{CPA} are the information contents related to the bond connectivity and atom type diversity

(13) Gutman molecular topological index based on simple vertex degree (*GMTI*)

$$S_G = \sum_{i=1}^{A}\sum_{j=1}^{A}\delta_i\delta_j d_{ij}$$

where $\delta_i\delta_j d_{ij}$ is the topological distance between vertex i and vertex j weighted by the product of the endpoint vertex degrees.

(14) Zagreb index with order 1 (*ZM1*)

The first Zagreb index (Weighted by vertex degrees) is given by

$$M1 = \sum_a \delta_a^2$$

where *a* runs over the A atoms of the molecule and δ is the vertex degree.

(15) Zagreb index with order 2 (*ZM2*)

$$M2 = \sum_b (\delta_i\delta_j)_b$$

where b runs over all the bonds in the molecule

The Zagreb indices are described on pg 509 of Handbook of Molecular Descriptors

(16) Modified Zagreb index with order 1 (*MZM1*)

(17) Modified Zagreb index with order 2 (*MZM2*)

(18) Quadratic index (*Qindex*)

$$Q = \frac{\sum_g (g^2 - 2g)^g F + 2}{2}$$

Quadratic index also called normalized quadratic index, where g are the different vertex degree values and ${}^{g}F$ is the vertex degree count.

(19) Largest value in the distance matrix (*diametert*)

$$D = \max_i(\eta_i)$$

$$\eta_i = \max_j(d_{ij})$$

η_i called atom eccentricity is the maximum distance from the *ith* vertex to the other vertices.

(20) Radius based on topology (*radiust*)

$$R = \min_i(\eta_i)$$

(21) Petitjean based on topology (*petitjeant*)

$$I_2 = \frac{D - R}{R}$$

(22) The logarithm of the simple topological index by Narumi (*Sito*)

$$S = \prod_{i=1}^{A} \delta_i$$

where A is the number of atoms, *Sito* is a molecular descriptor related to molecular branching proposed as the product of the vertex degrees.

(23) Harmonic topological index proposed by Narumi (*Hato*)

$$H = \frac{A}{\sum_{i=1}^{A} 1/\delta_i}$$

(24) Geometric topological index by Narumi (*Geto*)

$$G = \left(\prod_{i=1}^{A} \delta_i \right)^{1/A}$$

(25) Arithmetic topological index by Narumi (*Arto*)

$$A = \frac{\sum_{i=1}^{A} \delta_i}{A}$$

1.3 Molecular connectivity indices

1. Valence molecular connectivity Chi index for path order 0 ($^{0}\chi^{v}$)
2. Valence molecular connectivity Chi index for path order 1($^{1}\chi^{v}$)
3. Valence molecular connectivity Chi index for path order 2($^{3}\chi^{v}$)
4. Valence molecular connectivity Chi index for path order 3($^{4}\chi^{v}$)
5. Valence molecular connectivity Chi index for path order 4($^{5}\chi^{v}$)
6. Valence molecular connectivity Chi index for path order 5($^{6}\chi^{v}$)
7. Valence molecular connectivity Chi index for path order 6($^{7}\chi^{v}$)
8. Valence molecular connectivity Chi index for path order 7 ($^{8}\chi^{v}$)
9. Valence molecular connectivity Chi index for path order 8($^{9}\chi^{v}$)
10. Valence molecular connectivity Chi index for path order 9($^{10}\chi^{v}$)
11. Valence molecular connectivity Chi index for path order 10($^{11}\chi^{v}$)
12. Valence molecular connectivity Chi index for three cluster ($^{3}\chi^{v}{}_{c}$)
13. Valence molecular connectivity Chi index for four cluster ($^{4}\chi^{v}{}_{c}$)
14. Valence molecular connectivity Chi index for path/cluster ($^{4}\chi^{v}{}_{pc}$)
15. Valence molecular connectivity Chi index for cycles of 3 ($^{3}\chi^{v}{}_{CH}$)
16. Valence molecular connectivity Chi index for cycles of 4 ($^{4}\chi^{v}{}_{CH}$)
17. Valence molecular connectivity Chi index for cycles of 5 ($^{5}\chi^{v}{}_{CH}$)
18. Valence molecular connectivity Chi index for cycles of 6 ($^{6}\chi^{v}{}_{CH}$)
19. Simple molecular connectivity Chi indices for path order 0 ($^{0}\chi$)
20. Simple molecular connectivity Chi indices for path order 1 ($^{1}\chi$)
21. Simple molecular connectivity Chi indices for path order 2 ($^{2}\chi$)
22. Simple molecular connectivity Chi indices for path order 3 ($^{3}\chi_{p}$)
23. Simple molecular connectivity Chi indices for path order 4 ($^{4}\chi_{p}$)
24. Simple molecular connectivity Chi indices for path order 5 ($^{5}\chi_{p}$)
25. Simple molecular connectivity Chi indices for path order 6 ($^{6}\chi_{p}$)
26. Simple molecular connectivity Chi indices for path order 7 ($^{7}\chi_{p}$)
27. Simple molecular connectivity Chi indices for path order 8 ($^{8}\chi_{p}$)
28. Simple molecular connectivity Chi indices for path order 9 ($^{9}\chi_{p}$)

29. Simple molecular connectivity Chi indices for path order 10 (${}^{10}\chi_p$)
30. Simple molecular connectivity Chi indices for three cluster (${}^{3}\chi_c$)
31. Simple molecular connectivity Chi indices for four cluster (${}^{4}\chi_c$)
32. Simple molecular connectivity Chi indices for path/cluster (${}^{4}\chi_{pc}$)
33. Simple molecular connectivity Chi indices for cycles of 3 (${}^{3}\chi_{CH}$)
34. Simple molecular connectivity Chi indices for cycles of 4 (${}^{4}\chi_{CH}$)
35. Simple molecular connectivity Chi indices for cycles of 5 (${}^{5}\chi_{CH}$)
36. Simple molecular connectivity Chi indices for cycles of 6 (${}^{6}\chi_{CH}$)
37. mean chi1 (Randic) connectivity index (*mChi1*)
38. the difference between chi3c and chi4pc (*knotp*)
39. the difference between chi0v and chi0 (*dchi0*)
40. the difference between chi1v and chi1 (*dchi1*)
41. the difference between chi2v and chi2 (*dchi0*)
42. the difference between chi3v and chi3 (*dchi3*)
43. the difference between chi4v and chi4 (*dchi4*)
44. the difference between chiv3c and chiv4pc (*knotpv*)

Introduction:

1. Simple molecular connectivity index (No.19~36)

   The general formula for the molecular connectivity indices (${}^{m}\chi_t$) is as follows:

$$ {}^{m}\chi_q = \sum_{k=1}^{k} \left(\prod_{a=1}^{n} \delta_a\right)_k^{-1/2} $$

   where *k* runs over all of the *mth* order sub-graphs constituted by *n* atoms; *K* is the total number of *mth* order sub-graphs present in the molecular graph and in the case of the path sub-graphs equals the *mth* order path count ${}^{m}P$. The product is over the simple vertex degrees of all the vertices involved in each sub-graph. The subscript "*q*" for the connectivity indices refers to the type of molecular sub-graph and *ch* for chain or ring, *pc* for path-cluster, *c* for cluster, and *p* for path. For the first three path indices (${}^{0}\chi$, ${}^{1}\chi$, ${}^{2}\chi$), the calculation type, *p*, is often omitted from the variable name in the software.

2. Valence molecular connectivity indices (No.1~18)

   The valence connectivity indices (${}^{m}\chi^{v}_t$) are calculated in the same fashion as the simple connectivity

indices except that the vertex degree are replaced by the valence vertex degree, and the valence degree is given by: $\delta^v = Z^v - h = \sigma + \pi + n - h$. Where Z^v is the number of valence electrons, π is the number of electrons in *pi* orbital and n is the number of electrons in lone-pair orbitals.

The valence connectivity indices are described on page 86 of the Handbook of Molecular Descriptors. The connectivity indices are described in detail in the literature.

3. The remains connectivity indices are simple combination of the above simple connectivity indices and valence connectivity indices.

1.4 Kappa shape descriptors

1. Kappa alpha index for 1 bonded fragment ($^1\kappa_\alpha$)
2. Kappa alpha index for 2 bonded fragment ($^2\kappa_\alpha$)
3. Kappa alpha index for 3 bonded fragment ($^3\kappa_\alpha$)
4. Kier molecular flexibility index (*phi*)
5. Molecular shape Kappa index for 1 bonded fragment ($^1\kappa$)
6. Molecular shape Kappa index for 2 bonded fragment ($^1\kappa$)
7. Molecular shape Kappa index for 3 bonded fragment ($^1\kappa$)

Introduction:

(1) Kappa alpha index

The first order kappa shape index ($^1\kappa$) is given by

$$^1k = 2\,^1P_{\max}\,^1P_{\min} / (^1P_i)^2 = A(A-1)^2 / (^1P_i)^2$$

where P_i=# of paths of bond length i in the hydrogen suppressed molecule and A is the number of non hydrogen atoms in the molecule.

The second order kappa shape index ($^2\kappa$) is given by

$$^2k = 2\,^2P_{\max}\,^2P_{\min} / (^2P_i)^2 = (A-1)(A-2)^2 / (^2P_i)^2$$

The kappa shape indices are described on pg 248 of the Handbook of Molecular Descriptors.

The first order kappa alpha shape index ($^1\kappa_\alpha$) is given by

$$^{1}k_{a} = \frac{(A+a)(A+a-1)^{2}}{(^{1}P+a)^{2}}$$

where

$$a = 1 - \frac{r_x}{r_{x(sp^3)}}$$

where r_x is the covalent radius of the atom being evaluated and $r_{x(sp^3)}$ is the covalent radius of a carbon sp^3 atom (0.77Å).

The second order kappa alpha shape index ($^{2}\kappa_{\alpha}$) is given by

$$^{2}k_{a} = \frac{(A+a-1)(A+a-2)^{2}}{(^{2}P+a)^{2}}$$

The third order kappa alpha shape index ($^{3}\kappa_{\alpha}$) is given by

$$^{3}k_{a} = \frac{(A+a-1)(A+a-3)^{2}}{(^{3}P+a)^{2}} \quad \text{if A is odd}$$

$$^{3}k_{a} = \frac{(A+a-3)(A+a-2)^{2}}{(^{3}P+a)^{2}} \quad \text{if A is even}$$

The kappa shape indices are described on page 250 of the Handbook of Molecular Descriptors.

The kappa flexibility index (*phi*) is given by

$$phi = \frac{^{1}k_{a}\,^{2}k_{a}}{A}$$

The kappa flexibility index is described on page 178 of the Handbook of Molecular Descriptors.

1.5 Burden descriptors

1. Highest eigenvaluen.1 of Burden matrix/weighted by atomic masses (*bcutm1*)
2. Highest eigenvaluen.2 of Burden matrix/weighted by atomic masses (*bcutm2*)
3. Highest eigenvaluen.3 of Burden matrix/weighted by atomic masses (*bcutm 3*)
4. Highest eigenvaluen.4 of Burden matrix/weighted by atomic masses (*bcutm 4*)
5. Highest eigenvaluen.5 of Burden matrix/weighted by atomic masses (*bcutm 5*)
6. Highest eigenvaluen.6 of Burden matrix/weighted by atomic masses (*bcutm 6*)
7. Highest eigenvaluen.7 of Burden matrix/weighted by atomic masses (*bcutm7*)
8. Highest eigenvaluen.8 of Burden matrix/weighted by atomic masses (*bcutm8*)
9. Lowest eigenvaluen.1 of Burden matrix/weighted by atomic masses (*bcutm1*)
10. Lowest eigenvaluen.2 of Burden matrix/weighted by atomic masses (*bcutm2*)
11. Lowest eigenvaluen.3 of Burden matrix/weighted by atomic masses (*bcutm3*)
12. Lowest eigenvaluen.4 of Burden matrix/weighted by atomic masses (*bcutm4*)
13. Lowest eigenvaluen.5 of Burden matrix/weighted by atomic masses (*bcutm5*)
14. Lowest eigenvaluen.6 of Burden matrix/weighted by atomic masses (*bcutm6*)
15. Lowest eigenvaluen.7 of Burden matrix/weighted by atomic masses (*bcutm7*)
16. Lowest eigenvaluen.8 of Burden matrix/weighted by atomic masses (*bcutm8*)
17. Highest eigenvaluen.1 of Burden matrix/weighted by atomic vander Waals volumes (*bcutv1*)
18. Highest eigenvaluen.2 of Burden matrix/weighted by atomic vander Waals volumes (*bcutv2*)
19. Highest eigenvaluen.3 of Burden matrix/weighted by atomic vander Waals volumes (*bcutv3*)
20. Highest eigenvaluen.4 of Burden matrix/weighted by atomic vander Waals volumes(*bcutv4*)
21. Highest eigenvaluen.5 of Burden matrix/weighted by atomic vander Waals volumes (*bcutv5*)
22. Highest eigenvaluen.6 of Burden matrix/weighted by atomic vander Waals volumes (*bcutv6*)
23. Highest eigenvaluen.7 of Burden matrix/weighted by atomic vander Waals volumes (*bcutv7*)
24. Highest eigenvaluen.8 of Burden matrix/weighted by atomic vander Waals volumes (*bcutv8*)
25. Lowest eigenvaluen.1of Burden matrix/weighted by atomic vander Waals volumes (*bcutv1*)
26. Lowest eigenvaluen.2 of Burden matrix/weighted by atomic vander Waals volumes (*bcutv2*)
27. Lowest eigenvaluen.3 of Burden matrix/weighted by atomic vander Waals volumes (*bcutv3*)
28. Lowest eigenvaluen.4 of Burden matrix/weighted by atomic vander Waals volumes (*bcutv4*)
29. Lowest eigenvaluen.5 of Burden matrix/weighted by atomic vander Waals volumes (*bcutv5*)

30. Lowest eigenvaluen.6 of Burden matrix/weighted by atomic vander Waals volumes (*bcutv6*)

31. Lowest eigenvaluen.7of Burden matrix/weighted by atomic vander Waals volumes (*bcutv7*)

32. Lowest eigenvaluen.8 of Burden matrix/weighted by atomic vander Waals volumes (*bcutv8*)

33. Highest eigenvaluen.1 of Burden matrix/weighted by atomic Sanderson electronegativities (*bcute1*)

34. Highest eigenvaluen.2 of Burden matrix/weighted by atomic Sanderson electronegativities (*bcute2*)

35. Highest eigenvaluen.3 of Burden matrix/weighted by atomic Sanderson electronegativities (*bcute3*)

36. Highest eigenvaluen.4 of Burden matrix/weighted by atomic Sandersonel ectronegativities (*bcute4*)

37. Highest eigenvaluen.5 of Burden matrix/weighted by atomic Sanderson electronegativities (*bcute5*)

38. Highest eigenvaluen.6 of Burden matrix/weighted by atomic Sanderson electronegativities (*bcute6*)

39. Highest eigenvaluen.7of Burden matrix/weighted by atomic Sanderson electronegativities (*bcute7*)

40. Highest eigenvaluen.8 of Burden matrix/weighted by atomic Sanderson electronegativities (*bcute8*)

41. Lowest eigenvaluen.1 of Burden matrix/weighted by atomic Sanderson electronegativities (*bcute1*)

42. Lowes teigenvaluen.2 of Burden matrix/weighted by atomic Sanderson electronegativities (*bcute2*)

43. Lowest eigenvaluen.3 of Burden matrix/weighted by atomic Sanderson electronegativities (*bcute3*)

44. Lowest eigenvaluen.4 of Burden matrix/weighted by atomic Sanderson electronegativities (*bcute4*)

45. Lowest eigenvaluen.5 of Burden matrix/weighted by atomic Sanderson electronegativities (*bcute5*)

46. Lowest eigenvaluen.6 of Burden matrix/weighted by atomic Sanderson electronegativities (*bcute6*)

47. Lowesteigenvaluen.7 of Burden matrix/weighted by atomic Sanderson electronegativities (*bcute7*)

48. Lowest eigenvaluen.8 of Burden matrix/weighted by atomic Sanderson electronegativities (*bcute8*)

49. Highest eigenvaluen.1 of Burden matrix/weighted by atomic polarizabilities (*bcutp1*)

50. Highest eigenvaluen.2 of Burden matrix/weighted by atomic polarizabilities (*bcutp2*)

51. Highesteigenvaluen.3 of Burden matrix/weighted by atomic polarizabilities (*bcutp3*)

52. Highest eigenvaluen.4 of Burden matrix/weighted by atomic polarizabilities (*bcutp4*)

53. Highest eigenvaluen.5 of Burden matrix/weighted by atomic polarizabilities (*bcutp5*)

54. Highesteigenvaluen.6 of Burden matrix/weighted by atomic polarizabilities (*bcutp6*)

55. Highesteigenvaluen.7 of Burden matrix/weighted by atomic polarizabilities (*bcutp7*)

56. Highest eigenvaluen.8 of Burden matrix/weighted by atomic polarizabilities (*bcutp8*)

57. Lowes teigenvaluen.1 of Burden matrix/weighted by atomic polarizabilities (*bcutp1*)

58. Lowest eigenvaluen.2 of Burden matrix/weighted by atomic polarizabilities (*bcutp2*)

59. Lowest eigenvaluen.3 of Burden matrix/weighted by atomic polarizabilities (*bcutp3*)

60. Lowest eigenvaluen.4 of Burden matrix/weighted by atomic polarizabilities (*bcutp4*)

61. Lowest eigenvaluen.5 of Burden matrix/weighted by atomic polarizabilities (*bcutp5*)

62. Lowest eigenvaluen.6 of Burden matrix/weighted by atomic polarizabilities (*bcutp6*)

63. Lowest eigenvaluen.7of Burden matrix/weighted by atomic polarizabilities (*bcutp7*)

64. Lowest eigenvaluen.8 of Burden matrix/weighted by atomic polarizabilities (*bcutp8*)

Introduction:

The Burden eigenvalue descriptors are determined by solving the following general eigenvalue equation:

$$\mathbf{B}.\mathbf{V}=\mathbf{V}.\ \boldsymbol{e}$$

where **B** is a real connectivity matrix to be defined, **V** is a matrix of eigenvectors, and ***e*** is a diagonal matrix of eigenvalues. The rules defining **B** are as follows:

a. Hydrogen atoms are included.

b. The diagonal elements of **B**, B_{ii}, are either given by the carbon normalized atomic mass, vander Waals volume, Sanderson electronegativity, and polarizability of atom *i*.

c. The element of **B** connecting atoms *i* and *j*, B_{ij}, is equal to the square root of the bond order between atoms *i* and *j*.

d. All other elements of **B** (corresponding non bonded atom pairs) are set to 0.001.

The carbon normalized weights are as follows:

Symbol	Mass	VdWVolume	Electronegativity	Polarizability
C	1.000	1.000	1.000	1.000
H	0.084	0.299	0.942	0.381
N	1.167	0.695	1.160	0.625
O	1.332	0.512	1.327	0.455
P	2.579	1.181	0.916	2.063
S	2.670	1.088	1.076	1.648
F	1.582	0.410	1.455	0.318
Cl	2.952	1.000	1.265	1.239
Br	6.653	1.384	1.171	1.733
I	10.566	1.728	1.011	3.040
Si	2.339	1.424	0.778	3.057
As	6.238	1.181	1.025	2.449
Sn	9.884	2.041	0.836	4.375
Hg	16.702	0.695	0.800	3.239
Pb	17.252	1.538	0.833	3.864

The lowest eigenvalues are the absolute values of the negative eigenvalues. The highest eigenvalues are the eight largest positive eigenvalues. The Burden eigenvalues descriptors are described on the

Handbook of Molecular Descriptors (Todeschini and Consonni 2000)

1.6 Basak descriptors

(1) The information content with order 0 proposed by Basak (IC0)
(2) The information content with order 1 proposed by Basak(IC1)
(3) the information content with order 2 proposed by Basak(IC2)
(4) The information content with order 3 proposed by Basak(IC3)
(5) The information content with order 4 proposed by Basak(IC4)
(6) The information content with order 5 proposed by Basak(IC5)
(7) The information content with order 6 proposed by Basak(IC6)
(8) The structural information content with order 0 proposed by Basak (SIC0)
(9) The structural information content with order 1 proposed by Basak(SIC1)
(10) The structural information content with order 2 proposed by Basak(SIC2)
(11) The structural information content with order 3 proposed by Basak(SIC3)
(12) The structural information content with order 4 proposed by Basak(SIC4)
(13) The structural information content with order 5 proposed by Basak(SIC5)
(14) The structural information content with order 6 proposed by Basak(SIC6)
(15) The complementary information content with order 0 proposed by Basak(CIC0)
(16) The complementary information content with order 1 proposed by Basak(CIC1)
(17) The complementary information content with order 2 proposed by Basak(CIC2)
(18) The complementary information content with order 3 proposed by Basak(CIC3)
(19) The complementary information content with order 4 proposed by Basak(CIC4)
(20) The complementary information content with order 5 proposed by Basak(CIC5)
(21) The complementary information content with order 6 proposed by Basak(CIC6)

1.7 Electrotopological State Indices

1. Sum of E-State of atom type: sLi (*S1*)
2. Sum of E-State of atom type: ssBe (*S2*)
3. Sum of E-State of atom type: ssssBe (*S3*)
4. Sum of E-State of atom type: ssBH (*S4*)

5. Sum of E-State of atom type: sssB (*S5*)
6. Sum of E-State of atom type: ssssB (*S6*)
7. Sum of E-State of atom type: sCH3 (*S7*)
8. Sum of E-State of atom type: dCH2 (*S8*)
9. Sum of E-State of atom type: ssCH2 (*S9*)
10. Sum of E-State of atom type: tCH (*S10*)
11. Sum of E-State of atom type: dsCH (*S11*)
12. Sum of E-State of atom type: aaCH (*S12*)
13. Sum of E-State of atom type: sssCH (*S13*)
14. Sum of E-State of atom type: ddC (*S14*)
15. Sum of E-State of atom type: tsC (*S15*)
16. Sum of E-State of atom type: dssC (*S16*)
17. Sum of E-State of atom type: aasC (*S17*)
18. Sum of E-State of atom type: aaaC (*S18*)
19. Sum of E-State of atom type: ssssC (*S19*)
20. Sum of E-State of atom type: sNH3 (*S20*)
21. Sum of E-State of atom type: sNH2 (*S21*)
22. Sum of E-State of atom type: ssNH2 (*S22*)
23. Sum of E-State of atom type: dNH (*S23*)
24. Sum of E-State of atom type: ssNH (*S24*)
25. Sum of E-State of atom type: aaNH (*S25*)
26. Sum of E-State of atom type: tN (*S26*)
27. Sum of E-State of atom type: sssNH (*S27*)
28. Sum of E-State of atom type: dsN (*S28*)
29. Sum of E-State of atom type: aaN (*S29*)
30. Sum of E-State of atom type: sssN (*S30*)
31. Sum of E-State of atom type: ddsN (*S31*)
32. Sum of E-State of atom type: aasN (*S32*)
33. Sum of E-State of atom type: ssssN (*S33*)
34. Sum of E-State of atom type: sOH (*S34*)
35. Sum of E-State of atom type: dO (*S35*)

36. Sum of E-State of atom type: ssO (*S36*)

37. Sum of E-State of atom type: aaO (*S37*)

38. Sum of E-State of atom type: sF (*S38*)

39. Sum of E-State of atom type: sSiH3 (*S39*)

40. Sum of E-State of atom type: ssSiH2 (*S40*)

41. Sum of E-State of atom type: sssSiH (*S41*)

42. Sum of E-State of atom type: ssssSi (*S42*)

43. Sum of E-State of atom type: sPH2 (*S43*)

44. Sum of E-State of atom type: ssPH (*S44*)

45. Sum of E-State of atom type: sssP (*S45*)

46. Sum of E-State of atom type: dsssP (*S46*)

47. Sum of E-State of atom type: sssssP (*S47*)

48. Sum of E-State of atom type: sSH (*S48*)

49. Sum of E-State of atom type: dS (*S49*)

50. Sum of E-State of atom type: ssS (*S50*)

51. Sum of E-State of atom type: aaS (*S51*)

52. Sum of E-State of atom type: dssS (*S52*)

53. Sum of E-State of atom type: ddssS (*S53*)

54. Sum of E-State of atom type: sCl (*S54*)

55. Sum of E-State of atom type: sGeH3 (*S55*)

56. Sum of E-State of atom type: ssGeH2 (*S56*)

57. Sum of E-State of atom type: sssGeH (*S57*)

58. Sum of E-State of atom type: ssssGe (*S58*)

59. Sum of E-State of atom type: sAsH2 (*S59*)

60. Sum of E-State of atom type: ssAsH (*S60*)

61. Sum of E-State of atom type: sssAs (*S61*)

62. Sum of E-State of atom type: sssdAs (*S62*)

63. Sum of E-State of atom type: sssssAs (*S63*)

64. Sum of E-State of atom type: sSeH (*S64*)

65. Sum of E-State of atom type: dSe (*S65*)

66. Sum of E-State of atom type: ssSe (*S66*)

67. Sum of E-State of atom type: aaSe (*S67*)

68. Sum of E-State of atom type: dssSe (*S68*)

69. Sum of E-State of atom type: ddssSe (*S69*)

70. Sum of E-State of atom type: sBr (*S70*)

71. Sum of E-State of atom type: sSnH3 (*S71*)

72. Sum of E-State of atom type: ssSnH2 (*S72*)

73. Sum of E-State of atom type: sssSnH (*S73*)

74. Sum of E-State of atom type: ssssSn (*S74*)

75. Sum of E-State of atom type: sI (*S75*)

76. Sum of E-State of atom type: sPbH3 (*S76*)

77. Sum of E-State of atom type: ssPbH2 (*S77*)

78. Sum of E-State of atom type: sssPbH (*S78*)

79. Sum of E-State of atom type: ssssPb (*S79*)

80-158. maximum of E-State value of specified atom type (*Smax1~Smax79*)

159-237. minimum of E-State value of specified atom type (*Smin1~Smin79*)

Introduction:

The E-State value for a given non-hydrogen atom *i* in a molecule is given by its intrinsic state (I_i) plus the sum of the perturbations on that atom from all the other atoms in the molecule:

$$S_k = I_k + \sum_{i=1}^{A} \Delta I_{ki}$$

where the intrinsic state (I_k) is given by

$$I_k = \frac{(2/N)^2 \delta_k^v + 1}{\delta_k}$$

where N=principle quantum number (which is equal to the element’s period or row in the element table).

The perturbation of atom *k* due to atom *i* is given by

$$\Delta I_{ki} = \frac{(I_i - I_k)}{r_{ki}^{\ 2}}$$

where

$$r_{ki} = d_{ki} + 1$$

d_{ki} is the number of bonds that separate atom k from atom i.

The atom type non hydrogen indices (SX) are obtained by summing the E-State values for all the atoms of a given type t that are present in the molecule.

$$SX = \sum S(t)$$

In addition, the symbol present in molecular descriptors, *s*, *d*, *t* and *a* indicate single bond, double bond, triple bond and aromatic bond, respectively.

1.8 Autocorrelation descriptors

The Broto-Moreau autocorrelation descriptors (ATSdw) are given by

$$ATSdw = \sum_{i=1}^{A}\sum_{j=1}^{A} \delta_{ij}\omega_i\omega_j$$

where d is the considered topological distance (i.e. the lag in the autocorrelation terms), d $_{ij}$ is the Kronecker delta function (d $_{ij}$=1 if d_{ij}=d, zero otherwise), and w_i and w_j are the weights (normalized atomic properties) for atoms i and j respectively. The normalized atomic mass, van der Waals volume, electronegativity, or polarizability can be used for the weights. To match Dragon, the Broto-Moreau autocorrelation descriptors are calculated in the Software as follows:

$$ATS\mathrm{d}w = \ln\left(1 + \sum_{i=1}^{A}\sum_{j=1}^{A} \delta_{ij} \cdot w_i \cdot w_j\right)$$

The Moran autocorrelation descriptors (MATSdw) are given by

$$MATS\mathrm{d}w = \frac{\frac{1}{\Delta} \cdot \sum_{i=1}^{A}\sum_{j=1}^{A} \delta ij \cdot \left(w_i - \bar{w}\right)\left(w_j - \bar{w}\right)}{\frac{1}{A}\sum_{i=1}^{A}\left(w_i - \bar{w}\right)^2}$$

where $\bar{w}$ is the average value of the property for the molecule and $\triangle$ is the number of vertex pairs at distance equal to d.

The Geary autocorrelation descriptors are given by

$$GATSdw=\frac{\frac{1}{2\Delta}\cdot\sum_{i=1}^{A}\sum_{j=1}^{A}\delta ij\cdot\left(w_i-w_j\right)^2}{\frac{1}{A-1}\sum_{i=1}^{A}\left(w_i-\bar{w}\right)^2}$$

The 2D autocorrelation descriptors are described on page17-19 of the Handbook of Molecular Descriptors.

1.8.1 Moreau-Broto autocorrelation descriptors

1. Broto-Moreau autocorrelation of a topological structure-lag1/weighted by atomic masses (*ATSm1*)
2. Broto-Moreau autocorrelation of a topological structure-lag2/weighted by atomic masses (*ATSm2*)
3. Broto-Moreau autocorrelation of a topological structure-lag3/weighted by atomic masses (*ATSm3*)
4. Broto-Moreau autocorrelation of a topologicalstructure-lag4/weighted by atomic masses (*ATSm4*)
5. Broto-Moreau autocorrelation of a topological structure-lag5/weighted by atomic masses (*ATSm5*)
6. Broto-Moreau autocorrelation of a topological structure-lag6/weighted by atomic masses (*ATSm6*)
7. Broto-Moreau autocorrelation of a topological structure-lag7/weighted by atomic masses (*ATSm7*)
8. Broto-Moreau autocorrelation of a topological structure-lag8/weighted by atomic masses (*ATSm8*)
9. Broto-Moreau autocorrelation of a topological structure-lag1/weighted by atomic van der Waals volumes (*ATSv1*)
10. Broto-Moreau autocorrelation of a topological structure-lag2/weighted by atomic van der Waals volumes (*ATSv2*)
11. Broto-Moreau autocorrelation of a topological structure-lag3/weighted by atomic van der Waals volumes (*ATSv3*)
12. Broto-Moreau autocorrelation of a topological structure-lag4/weighted by atomic van der Waals volumes (*ATSv4*)
13. Broto-Moreau autocorrelation of a topological structure-lag5/weighted by atomic van der Waals volumes (*ATSv5*)

14. Broto-Moreau autocorrelation of a topological structure-lag6/weighted by atomi van der Waals volumes (*ATSv6*)
15. Broto-Moreau autocorrelation of a topological structure-lag7/weighted by atomic van der Waals volumes (*ATSv7*)
16. Broto-Moreau autocorrelation of a topological structure-lag8/weighted by atomic van der Waals volumes (*ATSv8*)
17. Broto-Moreau autocorrelation of a topological structure-lag1/weighted by atomic Sanderson electronegativities (*ATSe1*)
18. Broto-Moreau autocorrelation of a topological structure-lag2/weighted by atomic Sanderson electronegativities (*ATSe2*)
19. Broto-Moreau autocorrelation of a topological structure-lag3/weighted by atomic Sanderson electronegativities (*ATSe3*)
20. Broto-Moreau autocorrelation of a topological structure-lag4/weighted by atomic Sanderson electronegativities (*ATSe4*)
21. Broto-Moreau autocorrelation of a topological structure-lag5/weighted by atomic Sanderson electronegativities (*ATSe5*)
22. Broto-Moreau autocorrelation of a topological structure-lag6/weighted by atomic Sanderson electronegativities (*ATSe6*)
23. Broto-Moreau autocorrelation of a topological structure-lag7/weighted by atomic Sanderson electronegativities (*ATSe7*)
24. Broto-Moreau autocorrelation of a topological structure-lag8/weighted by atomic Sanderson electronegativities (*ATSe8*)
25. Broto-Moreau autocorrelation of a topological structure-lag1/weighted by atomic polarizabilities (*ATSp1*)
26. Broto-Moreau autocorrelation of a topological structure-lag2/weighted by atomic polarizabilities (*ATSp2*)
27. Broto-Moreau autocorrelation of a topological structure-lag3/weighted by atomic polarizabilities (*ATSp3*)
28. Broto-Moreau autocorrelation of a topological structure-lag4/weighted by atomic polarizabilities (*ATSp4*)

29. Broto-Moreau autocorrelation of a topological structure-lag5/weighted by atomic polarizabilities (*ATSp5*)

30. Broto-Moreau autocorrelation of a topological structure-lag6/weighted by atomic polarizabilities (*ATSp6*)

31. Broto-Moreau autocorrelation of a topological structure-lag7/weighted by atomic polarizabilities (*ATSp7*)

32. Broto-Moreau autocorrelation of a topological structure-lag8/weightedbyatomic polarizabilities (*ATSp8*)

1.8.2 Moran autocorrelation descriptors

33. Moran autocorrelation-lag1/weighted by atomic masses (*MATSm1*)
34. Moran autocorrelation-lag2/weighted by atomic masses (*MATSm2*)
35. Moran autocorrelation-lag3/weighted by atomic masses (*MATSm3*)
36. Moran autocorrelation-lag4/weighted by atomic masses (*MATSm4*)
37. Moran autocorrelation-lag5/weighted by atomic masses (*MATSm5*)
38. Moran autocorrelation-lag6/weighted by atomic masses (*MATSm6*)
39. Moran autocorrelation-lag7/weighted by atomic masses (*MATSm7*)
40. Moran autocorrelation-lag 8/weighted by atomic masses (*MATSm8*)
41. Moran autocorrelation-lag1/weighted by atomic van der Waals volumes (*MATSv1*)
42. Moran autocorrelation-lag2/weighted by atomic van der Waals volumes (*MATSv2*)
43. Moran autocorrelation-lag3/weighted by atomic van der Waals volumes (*MATSv3*)
44. Moran autocorrelation-lag4/weighted by atomic van der Waals volumes (*MATSv4*)
45. Moran autocorrelation-lag5/weighted by atomic van der Waals volumes (*MATSv5*)
46. Moran autocorrelation-lag6/weighted by atomic van der Waals volumes (*MATSv6*)
47. Moran autocorrelation-lag7/weighted by atomic van der Waals volumes (*MATSv7*)
48. Moran autocorrelation-lag8/weighted by atomic van der Waals volumes (*MATSv8*)
49. Moran autocorrelation-lag1/weighted by atomic Sanderson electronegativities (*MATSe1*)
50. Moran autocorrelation-lag2/weighted by atomic Sanderson electronegativities (*MATSe2*)
51. Moran autocorrelation-lag3/weighted by atomic Sanderson electronegativities (*MATSe3*)
52. Moran autocorrelation-lag4/weighted by atomic Sanderson electronegativities (*MATSe4*)

53. Moran autocorrelation-lag5/weighted by atomic Sanderson electronegativities (*MATSe5*)
54. Moran autocorrelation-lag6/weighted by atomic Sanderson electronegativities (*MATSe6*)
55. Moran autocorrelation-lag7/weighted by atomic Sanderson electronegativities (*MATSe7*)
56. Moran autocorrelation-lag8/weighted by atomic Sanderson electronegativities (*MATSe8*)
57. Moran autocorrelation-lag1/weighted by atomic polarizabilities (*MATSp1*)
58. Moran autocorrelation-lag2/weighted by atomic polarizabilities (*MATSp2*)
59. Moran autocorrelation-lag3/weighted by atomic polarizabilities (*MATSp3*)
60. Moran autocorrelation-lag4/weighted by atomic polarizabilities (*MATSp4*)
61. Moran autocorrelation-lag5/weighted by atomic polarizabilities (*MATSp5*)
62. Moran autocorrelation-lag6/weighted by atomic polarizabilities (*MATSp6*)
63. Moran autocorrelation-lag7/weighted by atomic polarizabilities (*MATSp7*)
64. Moran autocorrelation-lag8/weighted by atomic polarizabilities (*MATSp8*)

1.8.3 Geary autocorrelation descriptors

65. Geary autocorrelation-lag1/weighted by atomic masses (*GATSm1*)
66. Geary autocorrelation-lag2/weighted by atomic masses (*GATSm2*)
67. Geary autocorrelation-lag3/weighted by atomic masses (*GATSm3*)
68. Geary autocorrelation-lag4/weighted by atomic masses (*GATSm4*)
69. Geary autocorrelation-lag5/weighted by atomic masses (*GATSm5*)
70. Geary autocorrelation-lag6/weighted by atomic masses (*GATSm6*)
71. Geary autocorrelation-lag7/weighted by atomic masses (*GATSm7*)
72. Geary autocorrelation-lag8/weighted by atomic masses (*GATSm8*)
73. Geary autocorrelation-lag1/weighted by atomic van der Waals volumes (*GATSv1*)
74. Geary autocorrelation-lag2/weighted by atomic van der Waals volumes (*GATSv2*)
75. Geary autocorrelation-lag3/weighted by atomic van der Waals volumes (*GATSv3*)
76. Geary autocorrelation-lag4/weighted by atomic van der Waals volumes (*GATSv4*)
77. Geary autocorrelation-lag5/weighted by atomic van der Waals volumes (*GATSv5*)
78. Geary autocorrelation-lag6/weighted by atomic van der Waals volumes (*GATSv6*)
79. Geary autocorrelation-lag7/weighted by atomic van der Waals volumes (*GATSv7*)
80. Geary autocorrelation-lag8/weighted by atomic van der Waals volumes (*GATSv8*)
81. Geary autocorrelation-lag1/weighted by atomic Sanderson electronegativities (*GATSe1*)

82. Geary autocorrelation-lag2/weighted by atomic Sanderson electronegativities (*GATSe2*)
83. Gearyautocorrelation-lag3/weighted by atomic Sanderson electronegativities (*GATSe3*)
84. Geary autocorrelation-lag4/weighted by atomic Sanderson electronegativities (*GATSe4*)
85. Geary autocorrelation-lag5/weighted by atomic Sanderson electronegativities (*GATSe5*)
86. Geary autocorrelation-lag6/weighted by atomic Sanderson electronegativities (*GATSe6*)
87. Geary autocorrelation-lag7/weighted by atomic Sanderson electronegativities (*GATSe7*)
88. Geary autocorrelation-lag8/weighted by atomic Sanderson electronegativities (*GATSe8*)
89. Geary autocorrelation-lag1/weighted by atomic polarizabilities (*GATSp1*)
90. Geary autocorrelation-lag2/weighted by atomic polarizabilities (*GATSp2*)
91. Geary autocorrelation-lag3/weighted by atomic polarizabilities (*GATSp3*)
92. Geary autocorrelation-lag4/weighted by atomic polarizabilities (*GATSp4*)
93. Geary autocorrelation-lag5/weighted by atomic polarizabilities (*GATSp5*)
94. Geary autocorrelation-lag6/weighted by atomic polarizabilities (*GATSp6*)
95. Geary autocorrelation-lag7/weighted by atomic polarizabilities (*GATSp7*)
96. Geary autocorrelation-lag8/weighted by atomic polarizabilities (*GATSp8*)

1.9 Charge descriptors

1. Most positive charge on H atoms (Q_{Hmax})
2. Most positive charge on C atoms (Q_{Cmax})
3. Most positive charge on N atoms (Q_{Nmax})
4. Most positive charge on O atoms (Q_{Omax})
5. Most negative charge on H atoms (Q_{Hmin})
6. Most negative charge on C atoms ($\mathrm{Q_{Cmin}}$)
7. Most negative charge on N atoms ($\mathrm{Q_{Nmin}}$)
8. Most negative charge on O atoms ($\mathrm{Q_{Omin}}$)
9. Most positive charge in a molecule (Q_{max})
10. Most negative charge in a molecule (Q_{min})
11. Sum of squares of charges on H atoms (Q_{HSS})
12. Sum of squares of charges on C atoms (Q_{CSS})
13. Sum of squares of charges on N atoms (Q_{NSS})
14. Sum of squares of charges on O atoms (Q_{OSS})

15. Sum of squares of charges on all atoms (Q_{aSS})
16. Mean of positive charges (*Mpc*)
17. Total of positive charges (*Tpc*)
18. Mean of negative charges (*Mnc*)
19. Total of negative charges (*Tnc*)
20. Mean of absolute charges (*Mac*)
21. Total of absolute charges (*Tac*)
22. Relative positive charge (*Rpc*)
23. Relative negative charge (*Rnc*)
24. Submolecular polarity parameter (*SPP*)
25. Local dipole index (*LDI*)

Introduction:

These are electronic descriptors defined in terms of atomic charges and used to describe electronic aspects of the whole molecule and of particular regions, such as atoms, bonds and molecular fragments. Charge descriptors are calculated by computational chemistry and therefore can be considered among quantum chemical descriptors.

Electrical charges in the molecule are the driving force of electrostatic interactions, and it is well known the local electron density or charge plays a fundamental role in many chemical reactions and physic-chemical properties.

Some most used charge descriptors are displayed here as followed:

(1) Most positive charge in a molecule (Q_{max})

The maximum positive charge of the atoms in a molecule:

$$Q_{\max} = \max_a (q_a^+)$$

where q^+ are net atom positive charges

(2) Most negative charge in a molecule (Q_{min})

The maximum negative charge of the atoms in a molecule:

$$Q_{\min} = \max_a (q_a^-)$$

where q^- are net atom negative charges

(3) Total of positive charges (*Tpc*)

The sum of all of the positive charges of the atoms in a molecule:

$$Tpc = \sum_a (q_a^+)$$

where q^+ are net atom positive charges

(4) Total of negative charges (*Tnc*)

The sum of all of the negative charges of the atoms in a molecule:

$$Tnc = \sum_a (q_a^-)$$

where q^- are net atom negative charges

1.10 molecular properties

1. Molar refractivity (*MREF*)
2. LogP value based on the Crippen method (*logP*)
3. Square of LogP value based on the Crippen method (*logP2*)
4. Topological polarity surface area (*TPSA*)
5. Unsaturation index (*UI*)
6. Hydrophilic index (*Hy*)

Introduction:

(1) Molar refractivity (*MREF*)

Molecular descriptor of a liquid which contains both information about molecular volume and polarizability, usually defined by the Lorenz-Lorentz equation:

$$MR = \frac{n^2 - 1}{n^2 + 2} \frac{MW}{\rho}$$

where MW is the molecular weight, ρ is the liquid density, and n the refractive index of the liquid.

（2） LogP value based on the Crippen method (*logP*)

The Ghose-Crippen contribution method is based on hydrophobic atomic constants a_k measuring the lipophilic contributions of atoms in the molecule, each described by its neighbouring atoms.

$$LogP = \sum_k a_k N_k$$

where N_k is the occurrence of the *kth* atom type

（3） Topological polarity surface area (*TPSA*)

It is the sum of solvent-accessible surface areas of atoms with absolute value of partial charges greater than or equal to 0.2.

$$TPSA = \sum_a SA_a$$
$$|q_a| \geq 0.2$$

（4） Unsaturation index (*UI*)

The unsaturation index (*UI*) is defined as

$$UI = \log_2(1 + nDB + nTB + nAB)$$

where nDB=the number of double bonds, nTB=the number of triple bonds and nAB=the number of aromatic bonds. The unsaturation index is described in the user manual for Dragon.

(5) Hydrophilic index (*Hy*)

The hydrophilic index is given by

$$Hy = \frac{(1 + N_{Hy})\log_2(1 + N_{Hy}) + N_c(\frac{1}{A}\log_2\frac{1}{A}) + \sqrt{\frac{N_{Hy}}{A^2}}}{\log_2(1 + A)}$$

where N_{Hy} is the number of hydrophilic groups (or the total number of hydrogen attached to oxygen, sulfur and nitrogen atoms), N_c is the number of carbon atoms, and A is the number of non hydrogen atoms. The hydrophilic index is described in more detail on page 225 of the Handbook of Molecular Descriptors (Todeschini and Consonni 2000).

1.11 MOE-type descriptors

1. topological polar surface area based on fragments (*TPSA*)
2. Labute's Approximate Surface Area (*LabuteASA*)
3. MOE-type descriptors using SLogP contributions and surface area contributions (*SLOGPVSA1*)
4. MOE-type descriptors using SLogP contributions and surface area contributions (*SLOGPVSA2*)

5. MOE-type descriptors using SLogP contributions and surface area contributions (*SLOGPVSA3*)
6. MOE-type descriptors using SLogP contributions and surface area contributions (*SLOGPVSA4*)
7. MOE-type descriptors using SLogP contributions and surface area contributions (*SLOGPVSA5*)
8. MOE-type descriptors using SLogP contributions and surface area contributions (*SLOGPVSA6*)
9. MOE-type descriptors using SLogP contributions and surface area contributions (*SLOGPVSA7*)
10. MOE-type descriptors using SLogP contributions and surface area contributions (*SLOGPVSA8*)
11. MOE-type descriptors using SLogP contributions and surface area contributions(*SLOGPVSA9*)
12. MOE-type descriptors using SLogP contributions and surface area contributions(*SLOGPVSA10*)
13. MOE-type descriptors using SLogP contributions and surface area contributions(*SLOGPVSA11*)
14. MOE-type descriptors using SLogP contributions and surface area contributions(*SLOGPVSA12*)
15. MOE-type descriptors using MR contributions and surface area contributions (*SMRVSA1*)
16. MOE-type descriptors using MR contributions and surface area contributions (*SMRVSA2*)
17. MOE-type descriptors using MR contributions and surface area contributions (*SMRVSA3*)
18. MOE-type descriptors using MR contributions and surface area contributions (*SMRVSA4*)
19. MOE-type descriptors using MR contributions and surface area contributions (*SMRVSA5*)
20. MOE-type descriptors using MR contributions and surface area contributions (*SMRVSA6*)
21. MOE-type descriptors using MR contributions and surface area contributions (*SMRVSA7*)
22. MOE-type descriptors using MR contributions and surface area contributions (*SMRVSA8*)
23. MOE-type descriptors using MR contributions and surface area contributions (*SMRVSA9*)
24. MOE-type descriptors using MR contributions and surface area contributions (*SMRVSA10*)
25. MOE-type descriptors using partial charges and surface area contributions (*PEOEVSA1*)
26. MOE-type descriptors using partial charges and surface area contributions (*PEOEVSA2*)
27. MOE-type descriptors using partial charges and surface area contributions (*PEOEVSA3*)
28. MOE-type descriptors using partial charges and surface area contributions (*PEOEVSA4*)
29. MOE-type descriptors using partial charges and surface area contributions (*PEOEVSA5*)
30. MOE-type descriptors using partial charges and surface area contributions (*PEOEVSA6*)
31. MOE-type descriptors using partial charges and surface area contributions (*PEOEVSA7*)
32. MOE-type descriptors using partial charges and surface area contributions (*PEOEVSA8*)
33. MOE-type descriptors using partial charges and surface area contributions (*PEOEVSA9*)
34. MOE-type descriptors using partial charges and surface area contributions (*PEOEVSA10*)
35. MOE-type descriptors using partial charges and surface area contributions (*PEOEVSA11*)

36. MOE-type descriptors using partial charges and surface area contributions (*PEOEVSA12*)
37. MOE-type descriptors using partial charges and surface area contributions (*PEOEVSA13*)
38. MOE-type descriptors using partial charges and surface area contributions (*PEOEVSA14*)
39. MOE-type descriptors using Estate indices and surface area contributions (*EstateVSA1*)
40. MOE-type descriptors using Estate indices and surface area contributions (*EstateVSA2*)
41. MOE-type descriptors using Estate indices and surface area contributions (*EstateVSA3*)
42. MOE-type descriptors using Estate indices and surface area contributions (*EstateVSA4*)
43. MOE-type descriptors using Estate indices and surface area contributions (*EstateVSA5*)
44. MOE-type descriptors using Estate indices and surface area contributions (*EstateVSA6*)
45. MOE-type descriptors using Estate indices and surface area contributions (*EstateVSA7*)
46. MOE-type descriptors using Estate indices and surface area contributions (*EstateVSA8*)
47. MOE-type descriptors using Estate indices and surface area contributions (*EstateVSA9*)
48. MOE-type descriptors using Estate indices and surface area contributions (*EstateVSA10*)
49. MOE-type descriptors using Estate indices and surface area contributions (*EstateVSA11*)
50. MOE-type descriptors using surface area contributions and Estate indices (*VSAEstate1*)
51. MOE-type descriptors using surface area contributions and Estate indices (*VSAEstate2*)
52. MOE-type descriptors using surface area contributions and Estate indices (*VSAEstate3*)
53. MOE-type descriptors using surface area contributions and Estate indices (*VSAEstate4*)
54. MOE-type descriptors using surface area contributions and Estate indices (*VSAEstate5*)
55. MOE-type descriptors using surface area contributions and Estate indices (*VSAEstate6*)
56. MOE-type descriptors using surface area contributions and Estate indices (*VSAEstate7*)
57. MOE-type descriptors using surface area contributions and Estate indices (*VSAEstate8*)
58. MOE-type descriptors using surface area contributions and Estate indices (*VSAEstate9*)
59. MOE-type descriptors using surface area contributions and Estate indices (*VSAEstate10*)
60. MOE-type descriptors using surface area contributions and Estate indices (*VSAEstate11*)

1.12 Molecular fingerprint

Molecular fingerprints are string representations of chemical structures designed to enhance the efficiency of chemical database searching and analysis. They can encode the 2D and/or 3D features of molecules as an array of binary values or counts. Therefore, molecular fingerprints consist of bins, each

bin being a substructure descriptor associated with a specific molecular feature.

Molecular fingerprints directly encode molecular structure in a series of binary bits that represent the presence or absence of particular substructures in the molecule. Although it divides the whole molecule into a large number of fragments, it has the potential to keep overall complexity of drug molecules. Additionally, it does not need reasonable three-dimensional conformation of drug molecules and thereby does not lead to error accumulation from the description of molecular structures. Thus by means of such descriptors, each molecule can be described based on a set of fingerprints of structural keys, which is represented as a Boolean array. A SMARTS list of substructure patterns is first determined as a predefined dictionary. There is a one-to-one correspondence between each SMARTS pattern and bit in the fingerprint. For each SMARTS pattern, if its corresponding substructure is present in the given molecule, the corresponding bit in the fingerprint is set to 1; conversely, it is set to 0 if the substructure is absent in the molecule (see Figure 1). Note that different molecular fingerprint systems abstract and magnify different aspects of molecular topology.

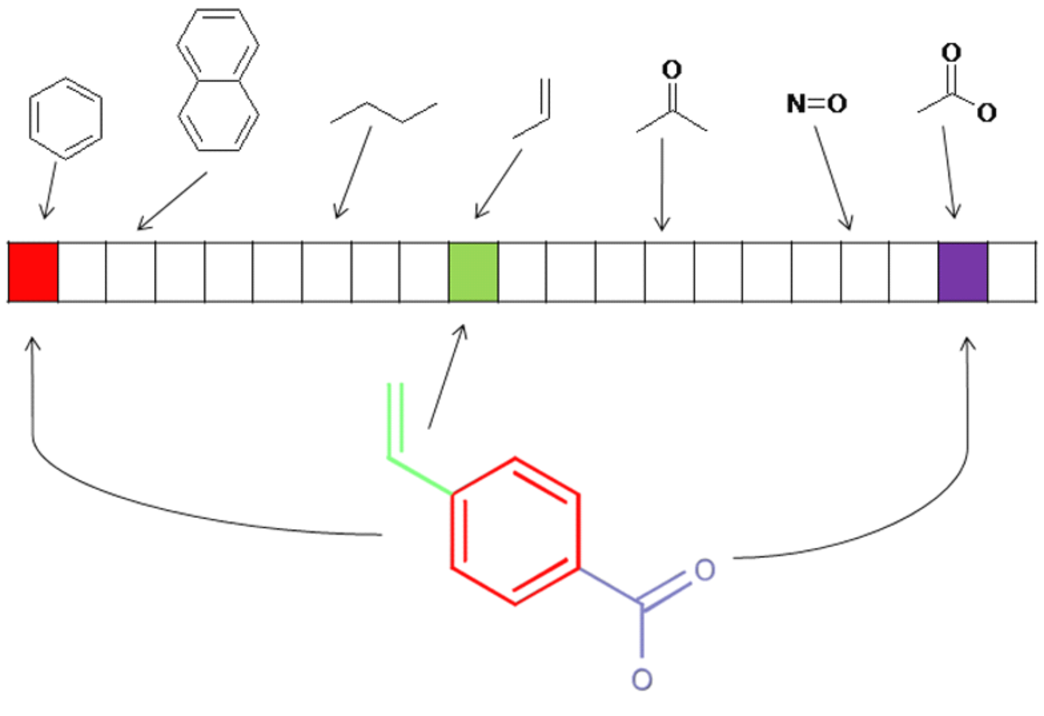

Figure 1 Representation of a molecular substructure fingerprint with a substructure fingerprint dictionary of given substructure patterns. This molecule is represented in a series of binary bits that represent the presence or absence of particular substructures in the molecules. This Figure is from Ref.

2 in section 3 and 4.

1.12.1 Daylight-type fingerprint

The Daylight fingerprints (DFP) are hashed fingerprints encoding each atom type, all Augmented Atoms and all paths of length 2–7 atoms, giving a total string of 1024 bits [Daylight-James, Weininger et al., 1997].

1.12.2 MACCS keys and FP4 fingerprint

The FP4 and MACCS fingerprints are used to construct the substructure dictionaries, respectively. The dictionary of FP4 fingerprint contains 307 mostly common substructure patterns. It is originally written in an attempt to represent the classification of organic compounds from the viewpoint of an organic chemist. The MACCS fingerprint uses a dictionary of MDL keys, which contains a set of 166 mostly common substructure features. These are referred to as the MDL public MACCS keys. Both the definitions of FP4 and MACCS fingerprints are available from OpenBabel (version 2.3.0, http://openbabel.org/, accessed October, 2010). All calculations for these substructure fingerprints are performed in PyDPI package, developed by our group.

1.12.3 E-state fingerprint

Electrotopological State (E-state) fingerprints represent the presence/absence of 79 E-state substructures defined Kier and Hall in a molecule. The definition of 79 atom types can be found in section 1.5.

1.12.4 Atom pairs and topological torsions fingerprints

Atom pairs fingerprint:

Atom pairs are substructure descriptors defined in terms of any pair of atoms and bond types connecting them. An atom pair is composed of two non-hydrogen atoms and an interatomic separation:

$$\mathrm{AP} = \left\{[i\text{th atom description}][\text{separation}][j\text{th atom description}]\right\}$$

The two considered atoms need not be directly connected and the separation can be the topological distance between them [Carhart, Smith et al., 1985]; these descriptors are usually called topological

atom pairs being based on the topological representation of the molecules. Atom type is defined by the element itself, the number of heavy-atom connections and number of p electron pairs on each atom. Unlike topological torsions, atom pairs are sensitive to long-range correlations between the atoms in molecules and therefore to small changes in one part of even large molecules. Atom pair descriptors usually are Boolean variables encoding the presence or absence of a particular atom pair in each molecule.

Topological torsion fingerprint:

The topological torsion descriptor (TT) is related to the 4-atom linear subfragment descriptor of Klopman because it is defined as a Boolean variable for the presence/absence of a linear sequence of four consecutively bonded non-hydrogen atoms *k–i–j–l*, each described by its atom type (TYPE), the number of *p* electrons (NPI) on each atom, and the number of non-hydrogen atoms (NBR) bonded to it [Nilakantan, Bauman et al., 1987]. Usually NBR does not include *k–i–j–l* atoms that go to make the torsion itself; therefore, it is -1 for k and l atoms and -2 for the two central atoms *i* and *j*. The torsion around the *i-j* bond and defined by the four indices *k–i–j–l* is represented by the following TT descriptor:

$$TT = \{[NPI-TYPE-NBR]_k [NPI-TYPE-NBR]_i [NPI-TYPE-NBR]_j [NPI-TYPE-NBR]_l\}$$

The TT descriptor is a topological analogue of the 3D torsion angle, defined by four consecutively bonded atoms. The topological torsion is a short-range descriptor, that is, it is sensitive only to local changes in the molecule and is independent of the total number of atoms in the molecule.

The use of atom-centered fragments and related descriptors greatly increases the specific chemical information concerning different functional groups, but cannot discriminate between different arrangements of functional groups within a molecule.

1.12.5 Morgan fingerprint

This family of fingerprints, better known as circular fingerprints, is built by applying the Morgan algorithm to a set of user-supplied atom invariants. When generating Morgan fingerprints, the radius of the fingerprint need be provided. For detailed information about Morgan fingerprint, please refer to Ref. [19]. Note the default atom invariants use connectivity information similar to those used for the well known ECFP family of fingerprints. When comparing the ECFP/FCFP fingerprints and the Morgan fingerprints generated by the PyDPI, remember that the 4 in ECFP4 corresponds to the diameter of the

atom environments considered, while the Morgan fingerprints take a radius parameter. So the examples above, with radius=2, are roughly equivalent to ECFP4 and FCFP4.

References:

[1] Aguiara, P.F.d., Bourguignon, B., Khotsa, M.S., Massarta, D.L., and Phan-Than-Luub, R. D-optimal designs. Chemometrics and Intelligent Laboratory Systems. 1995, 30, 199-210.

[2] Daylight Chemical Information Systems, Inc. Simplified Molecular Input Line Entry System. 2006, http://www.daylight.com/smiles/index.html.

[3] Elsevier MDL. MDL QSAR Version 2.2. 2006, http://www.mdl.com/products/predictive/qsar/index.jsp.

[4] Ghose, A.K., Viswanadhan,V. N., and Wendoloski, J.J. Prediction of Hydrophilic (Lipophilic) Properties of Small Organic Molecules Using Fragmental Methods: An analysis of ALOG an CLOGP Methods. J. Phys. Chem. 1998, 102, 3762-3772.

[5] Gramatica, P., Corradi, M., and Consonni, V. Model ligand Prediction of Soil Sorption Coefficients of Non-ionic Organic Pesticides by Molecular Descriptors. Chemosphere 2000, 41, 763-777.

[6] Hall, L.H., and Kier, L.B. The Molecular Connectivity Chi Indices and Kappa Shape Indices in Structure-Property Relations. In Reviews of Computational Chemistry, edited by D. Boyd and K. Lipkowitz. New York: VCH Publishers, Inc., 1991, 367-422.

[7] Hall, L.H., and Kier, L.B. Molecular Connectivity Chi Indices for Database Analysis and Structure-Property Modeling. In Methods for QSAR Modelling, edited by J. Devillers. 1999

[8] Kier,L.B. Inclusion of symmetry as a shape attribute in Kappa index analysis. Quantit. Struct.-Act. Relat. 1987, 6, 8-12.

[9] Kier, L.B., and Hall, L.H. Molecular Connectivity in Chemistry and Drug Research. 1976, New York: Academic Press Inc.

[10] Kier, L.B.,and Hall, L.H. Molecular Connectivity in Structure-Activity Analysis. 1986, New York: John Wiley and Sons.

[11] Kier,L.B., and Hall, L.H. Molecule Structure Description: The Electrotopological State. 1999, New York: Academic Press.

[12] Martin, T.M., Harten, P., Venkatapathy, R., Das, S., and Young, D.M. A Hierarchical Clustering Methodology for the Estimation of Toxicity. Toxicology Mechanisms and Methods 2008, 18, 251-266.

[13] JAMA : A Java Matrix Package. 2005, http://math.nist.gov/javanumerics/jama/.

[14] Talete. Dragon Version 5.4. 2006, http://www.talete.mi.it/dragon_net.htm.

Todeschini, R., and Consonni, V. Handbook of Molecular Descriptors. 2000, Weinheim, Germany: Wiley-VCH.

[15] Viswanadhan, V.N., Ghose, A.K., Revankar, G. R., and Robins, R.K. Atomic Physicochemical Parameters for Three Dimensional Structure Directed Quantitative Structure-Activity Relationships. 4. Additional Parameters for Hydrophobic and Dispersive Interactions and Their Application for an Automated Superposition of Certain Naturally Occurring Nucleoside Antibiotics. J. Chem. Inf. Comput. Sci. 1989, 29, 163-172.

[16] Wang, R., Gao, Y., and Lai, L. Calculating partition coefficient by atom-additive method. Perspectives in Drug Discovery and Design 2000, 19, 47-66.

[17] R. E. Carhart, D.H. Smith, R. Venkataraghavan. Atom Pairs as Molecular Features in Structure-Activity Studies: Definition and Applications. J. Chem. Inf. Comput. Sci. 1985, 265, 64-73.

[18] R. Nilakantan, N. Bauman, J.S. Dixon, R. Venkataraghavan. Topological Torsions: A New Molecular Descriptor for SAR Applications. Comparison with Other Descriptors. J. Chem. Inf. Comput. Sci. 1987, 27, 82-85.

[19] David Rogers, Mather Hahn. Extended-Connectivity Fingerprints. J. Chem. Inf. Comput. Sci. 2010, 50, 742-754.

[20] Paul Labute. A widely applicable set of descriptors. Journal of Molecular Graphics and Modeling. 2000, 18, 464-477.

[21] C. A. James, D. Weininger, J. Delany, Daylight Theory Manual 1997, http://www.daylight.com/dayhtml/doc/theory/theory.toc.html.

[22] Burden, F.R. A chemically intuitive molecular index based on the eigenvalues of a modified adjacency matrix. Quant. Struct. -Act. Relat., 1997, 16, 309–314

[23] Basak, S.C. Information theoretic indices of neighborhood complexity and their applications, in Bibliography Topological Indices and Related Descriptors in QSAR and QSPR (eds J. Devillers and A.T. Balaban), Gordon and Breach Science Publishers, Amsterdam, The Netherlands, 1999, pp. 563–593.

2 Descriptors of proteins and peptides

A protein or peptide sequence with N amino acid residues is expressed as: R_1, R_2, R_3, …, R_N, where R_i represents the residue at the i-th position in the sequence. The labels i and j are used to index amino acid position in a sequence and r, s are used to index the amino acid type. The computed features are divided into 4 groups according to their known applications described in the literature.

A protein sequence can be divided equally into segments and the methods, described as follows for the global sequence, can be applied to each segment.

2.1 Amino acid composition

The amino acid composition is the fraction of each amino acid type within a protein. The fractions of all 20 natural amino acids are calculated as:

$$f(r)=\frac{N_r}{N} \qquad r=1, 2, 3, ..., 20$$

Where N_r is the number of the amino acid type r and N is the length of the sequence.

2.2 Dipeptide composition

The dipeptide composition gives 400 features, defined as:

$$f(r,s)=\frac{N_{rs}}{N-1} \qquad r, s=1, 2, 3, ..., 20$$

where N_{rs} is the number of dipeptide represented by amino acid type r and s.

2.3 Tripeptide composition

The tripeptide composition gives 8000 features, defined as:

$$f(r,s,t)=\frac{N_{rst}}{N-2} \qquad r, s=1, 2, 3, ..., 20$$

where N_{rst} is the number of tripeptide represented by amino acid type r, s and t.

2.4 Autocorrelation descriptors

Autocorrelation descriptors are defined based on the distribution of amino acid properties along the sequence. The amino acid properties used here are various types of amino acids index (http://www.genome.ad.jp/dbget/aaindex.html).Three type of autocorrelation descriptors are used here and are described as following.

All the amino acid indices are centralized and standardized before the calculation, i.e.

$$P_r = \frac{P_r - \overline{P}}{\sigma}$$

Where $\overline{P}$ is the average of the property of the 20 amino acids.

$$\overline{P} = \frac{\sum_{r=1}^{20} P_r}{20} \quad \text{and} \quad \sigma = \sqrt{\frac{1}{20}\sum_{r=1}^{20}(P_r - \overline{P})^2}$$

2.4.1 Normalized Moreau-Broto autocorrelation descriptors

Moreau-Broto autocorrelation descriptors application to protein sequences may be defined as:

$$AC(d) = \sum_{i=1}^{N-d} P_i P_{i+d} \qquad d\text{=1, 2, 3, ..., nlag}$$

Where d is called the lag of the autocorrelation and P_i and P_{i+d} are the properties of the amino acids at position i and $i+d$, respectively. $nlag$ is the maximum value of the lag.

The normalized Moreau-Broto autocorrelation descriptors are defined as:

$$ATS(d) = \frac{AC(d)}{N-d} \qquad d=1, 2, 3, ..., nlag$$

Database: AAindex
Entry: ANDN920101
LinkDB: ANDN920101

```
H ANDN920101
D alpha-CH chemical shifts (Andersen et al., 1992)
R LIT:1810048b PMID:1575719
A Andersen, N.H., Cao, B. and Chen, C.
T Peptide/protein structure analysis using the chemical shift index method:
  upfield alpha-CH values reveal dynamic helices and aL sites
J Biochem. and Biophys. Res. Comm. 184, 1008-1014 (1992)
C BUNA790102    0.949
I    A/L     R/K     N/M     D/F     C/P     Q/S     E/T     G/W     H/Y     I/V
    4.35    4.38    4.75    4.76    4.65    4.37    4.29    3.97    4.63    3.95
    4.17    4.36    4.52    4.66    4.44    4.50    4.35    4.70    4.60    3.95
//
```

Figure 2 An illustrated example in the AAIndex database

2.4.2 Moran autocorrelation

Moran autocorrelation descriptors application to protein sequence may be defined as:

$$I(d) = \frac{\frac{1}{N-d}\sum_{i=1}^{N-d}(P_i-\overline{P})(P_{i+d}-\overline{P})}{\frac{1}{N}\sum_{i=1}^{N}(P_i-\overline{P})^2} \qquad d=1, 2, 3, ..., 30.$$

Where d and P_i and P_{i+d} are defined in the same way as in 2.2.1, and is the average of the considered property P along the sequence, i.e.,

$$\overline{P} = \frac{\sum_{i=1}^{N} P_i}{N}$$

Where d, $\overline{P}$, P_i and P_{i+d}, *nlag* have the same meaning as in the above.

2.4.3 Geary autocorrelation Descriptors

Geary autocorrelation descriptors application to protein sequence may be defined as:

$$C(d)=\frac{\frac{1}{2(N-d)}\sum_{i=1}^{N-d}(P_i-P_{i+d})^2}{\frac{1}{N-1}\sum_{i=1}^{N}(P_i-\overline{P})^2}\qquad d=1, 2, 3, ..., 30.$$

Where d, $\overline{P}$, P_i and P_{i+d}, $nlag$ have the same meaning as in the above.

The amino acid indices used in these autocorrelation descriptors can be specified in file "input-param.dat" from "input-aaindexdb.dat".

For each amino acid index, there will be $3\times nlag$ autocorrelation descriptors.

2.5 Composition, transition and distribution

These descriptors are developed by Dubchak, et.al.

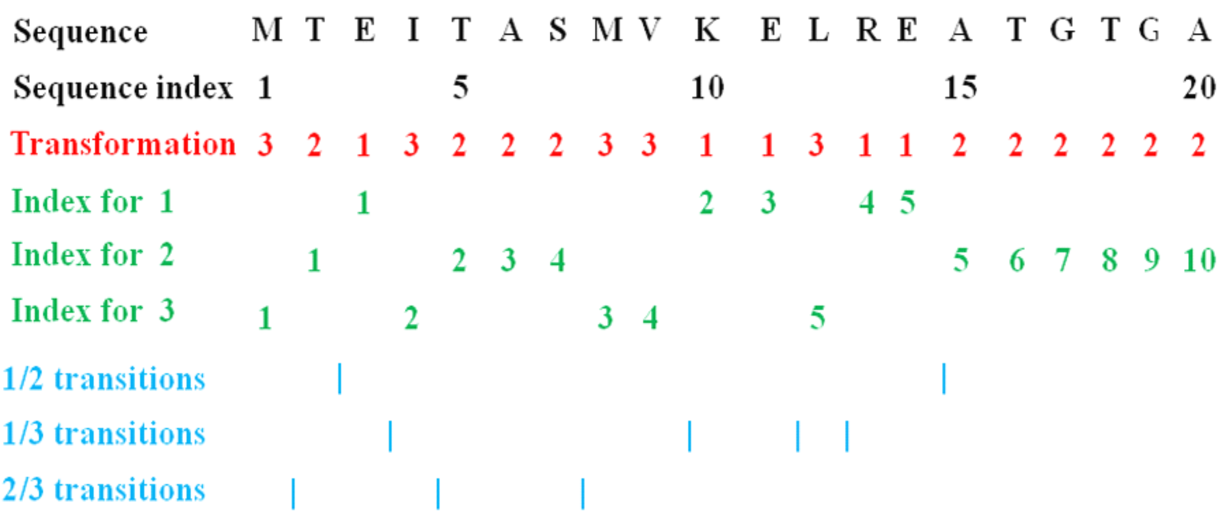

Figure 3 The sequence of a hypothetic protein indicating the construction of composition, transition and distribution descriptors of a protein. Sequence index indicates the position of an amino acid in the sequence. The index for each type of amino acids in the sequence ('1' '2' or '3') indicates the position of the first, second, third, ... of that type of amino acid. 1/2 transition indicates the position of '12' or '21' pairs in the sequence (1/3 and 2/3 are defined in the same way.). This figure is from Ref. 2 in section 3 and 4.

Step1. Sequence encoding

The amino acids are divided in three classes according to its attribute and each amino acid is encoded by one of the indices 1, 2, 3 according to which class it belonged. The attributes used here include

hydrophobicity, normalized van der Waals volume polarity, and polarizability, as in the references. The corresponding division is in the table 1.

Table 1 Amino acid attributes and the division of the amino acids into three groups for each attribute

	**Group 1**	**Group 2**	**Group 3**
hydrophobicity	Polar<br>R,K,E,D,Q,N	Neutral<br>G, A, S,T,P,H,Y	Hydrophobicity<br>C,L,V,I,M,F,W
normalized van der Waals volume	0-2.78<br>G,A,S,T,P,D	2.95-4.0<br>N,V,E,Q,I,L	4.03-8.08<br>M,H,K,F,R,Y,W
polarity	4.9-6.2<br>L,I,F,W,C,M,V,Y	8.0-9.2<br>P,A,T,G,S	10.4-13.0<br>H,Q,R,K,N,E,D
polarizability	0-1.08<br>G,A,S,D,T	0.128-0.186<br>C,P,N,V,E,Q,I,L	0.219-0.409<br>K,M,H,F,R,Y,W
charge	Positive<br>K,R	Neutral<br>A,N,C,Q,G,H,I,L,M,F,P,S,T,W,Y, V	Negative<br>D,E
secondary structure	Helix<br>E,A,L,M,Q,K,R,H	Strand<br>V,I,Y,C,W,F,T	Coil<br>G,N,P,S,D
solvent accessibility	Buried<br>A,L,F,C,G,I,V,W	Exposed<br>R,K,Q,E,N,D	Intermediate<br>M,S P,T,H,Y

For example, for a given sequence “MTEITAAMVKELRESTGAGA”, it will be encoded as “32132223311311222222” according to its hydrophobicity division.

Step 2: Composition, Transition and Distribution descriptors

Three descriptors, “Composition (C)”, “Transition (T)”, and “Distribution (D)” were calculated for a given attribute as follows:

Composition: It is the global percent for each encoded class in the sequence. In the above example using hydrophobicity division, the numbers for encoded classes “1”, “2”, “3” are 5, 10, 5 respectively, so the compositions for them are 5/20=25%, 10/20=10%, and 5/20=25% respectively, where 20 is the length of the protein sequence. Composition can be defined as:

$$C_r = \frac{n_r}{n} \qquad r\text{=1, 2, 3}$$

Where n_r is the number of r in the encoded sequence and N is the length of the sequence.

Transition: A transition from class 1 to 2 is the percent frequency with which 1 is followed by 2 or 2 is followed by 1 in the encoded sequence. Transition descriptor can be calculated as:

$$T_{rs} = \frac{n_{rs} + n_{sr}}{N - 1} \qquad rs = "12", "13", "23"$$

Where n_{rs}, n_{sr} is the numbers of dipeptide encoded as "*rs*" and "*sr*" respectively in the sequence and N is the length of the sequence.

Distribution: The "distribution" descriptor describes the distribution of each attribute in the sequence. There are five "distribution" descriptors for each attribute and they are the position percents in the whole sequence for the first residue, 25% residues, 50% residues, 75% residues and 100% residues , respectively, for a specified encoded class. For example, there are10 residues encoded as "2" in the above example, the positions for the first residue "2", the 2th residue "2" (25%*10=2), the 5th "2" residue (50%*10=5), the 7th "2" (75%*10=7) and the10th residue "2" (100%*10) in the encoded sequence are 2, 5, 15, 17, 20 respectively, so the distribution descriptors for "2" are: 10.0 (2/20*100), 25.0 (5/20*100), 75.0 (15/20*100), 85.0 (17/20*100) , 100.0 (20/20*100), respectively.

2.6 Conjoint Triad Descriptors

Conjoint triad descriptors are proposed by J.W. Shen et.al. These conjoint triad features abstracts the features of protein pairs based on the classification of amino acids. In this approach, each protein sequence is represented by a vector space consisting of features of amino acids. To reduce the dimensions of vector space, the 20 amino acids were clustered into several classes according to their dipoles and volumes of the side chains. The conjoint triad features are calculated as follows:

Step 1: classification of amino acids

Electrostatic and hydrophobic interactions dominate protein-protein interactions. These two kinds of interactions may be reflected by the dipoles and volumes of the side chains of amino acids, respectively. Accordingly, these two parameters were calculated, respectively, by using the density-functional theory method B3LYP/6-31G and molecular modeling approach. Based on the dipoles and volumes of the side chains, the 20 amino acids could be clustered into seven classes (See Table 2). Amino acids within the same class likely involve synonymous mutations because of their similar characteristics.

Table 2 Classification of amino acids based on dipoles and volumes of the side chains

No.	Dipole scale[a]	Volume scale[b]	Class
1	-	-	Ala, Gly, Val
2	-	+	Ile, Leu, Phe, Pro
3	+	+	Tyr, Met, Thr, Ser
4	++	+	His, Asn, Gln, Tpr
5	+++	+	Arg, Lys
6	+'+'+'	+	Asp, Glu
7	+ [c]	+	Cys

[a] Dipole scale (Debye): -, Dipole<1.0; +, 1.0<Dipole<2.0; ++, 2.0<Dipole<3.0; +++, Dipole>3.0; +'+'+', Dipole>3.0 with opposite orientation. [b] Volume scale ($Å^3$): -, Volume<50; +, Volume> 50. [c] Cys is separated from class 3 because of its ability to form disulfide bonds. This table is from Ref. 13.

Step 2: Conjoint triad calculation

The conjoint triad descriptors considered the properties of one amino acid and its vicinal amino acids and regarded any three continuous amino acids as a unit. Thus, the triads can be differentiated according to the classes of amino acids, i.e., triads composed by three amino acids belonging to the same classes, such as ART and VKS, could be treated identically. To conveniently represent a protein, we first use a binary space (**V**, **F**) to represent a protein sequence. Here, **V** is the vector space of the sequence features, and each feature v_i represents a sort of triad type; **F** is the frequency vector corresponding to **V**, and the value of the ith dimension of **F** (f_i) is the frequency of type v_i appearing in the protein sequence. For the amino acids that have been catalogued into seven classes, the size of **V** should be 7×7×7; thus i = 1,2, ..., 343. The detailed description for (**V**, **F**) is illustrated in Figure 3. Clearly, each protein correlates to the length (number of amino acids) of protein. In general, a long protein would have a large value of f_i, which complicates the comparison between two heterogeneous proteins. Thus, we defined a new parameter, d_i, by normalizing f_i with the following equation.

$$d_i = (f_i - \min\{f_1, f_2, f_3, ..., f_{343}\}) / \max\{f_1, f_2, f_3, ..., f_{343}\}$$

The numerical value of d_i of each protein ranges from 0 to 1, which thereby enables the comparison between proteins. Accordingly, we obtain another vector space (designated **D**) consisting of d_i to represent protein

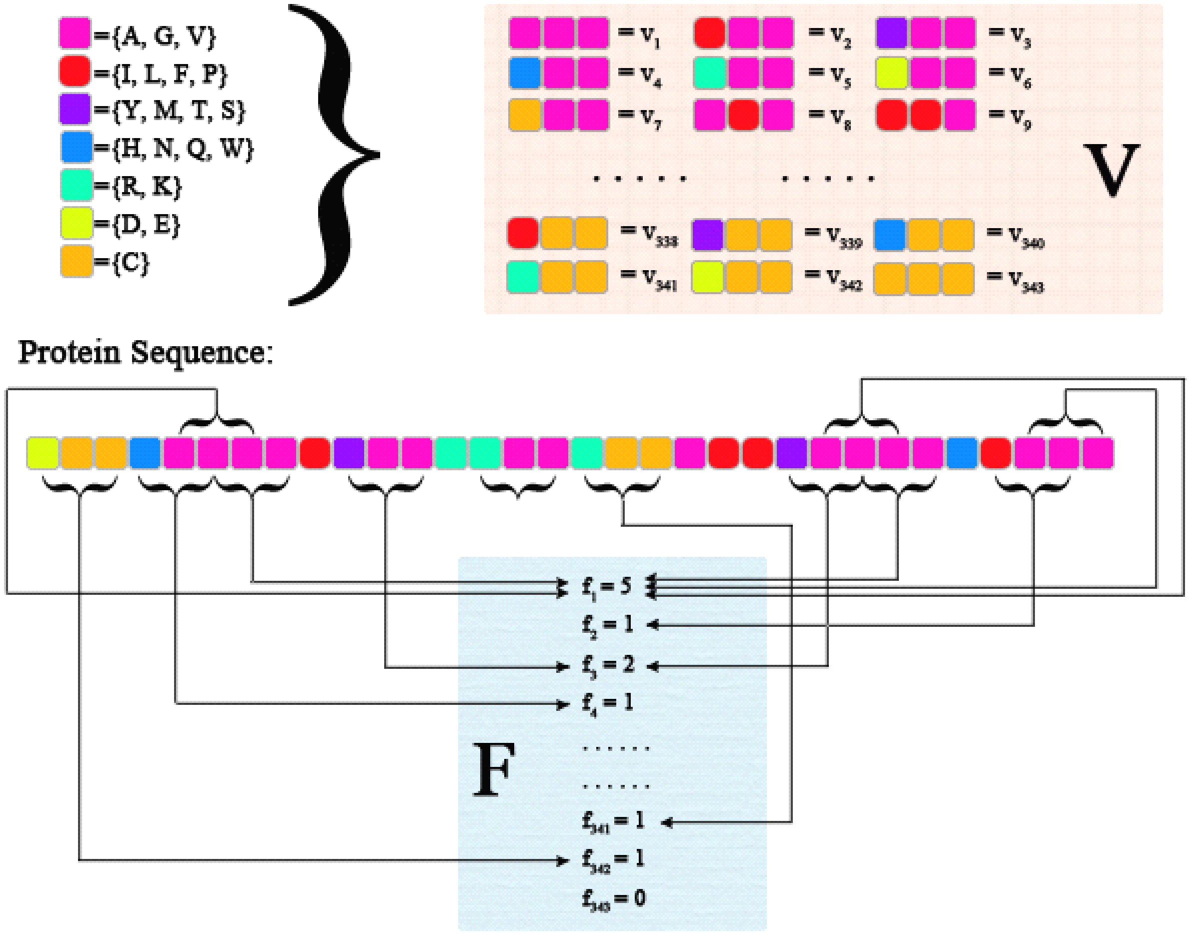

Figure 3 Schematic diagram for constructing the vector space (V, F) of protein sequence. V is the vector space of the sequence features; each feature (v_i) represents a triad composed of three consecutive amino acids; F is the frequency vector corresponding to V, and the value of the ith dimension of F(f_i) is the frequency that $\mathbf{v}_i$ triad appeared in the protein sequence. This figure is from Ref. 13.

2.7 Quasi-sequence-order Descriptors

The quasi-sequence-order descriptors are proposed by K.C. Chou, et.al. They are derived from the distance matrix between the 20 amino acids.

2.7.1 Sequence-order-coupling numbers

The dth-rank sequence-order-coupling number is defined as:

$$\tau_d = \sum_{i=1}^{N-d} (d_{i,i+d})^2 \qquad d=1, 2, 3, \ldots, maxlag$$

Where $d_{i,i+d}$ is the distance between the two amino acids at position i and $i+d$.

Note: *maxlag* is the maximum lag and the length of the protein must be not less than *maxlag*.

2.7.2 Quasi-sequence-order (QSO) descriptors

For each amino acid type, a quasi-sequence-order descriptor can be defined as:

$$X_r = \frac{f_r}{\sum_{r=1}^{20} f_r + w \sum_{d=1}^{maxlag} \tau_d} \qquad r=1, 2, 3, \ldots, 20$$

Where f_r is the normalized occurrence for amino acid type i and w is a weighting factor (w=0.1). These are the first 20 quasi-sequence-order descriptors. The other 30 quasi-sequence-order are defined as:

$$X_r = \frac{w\tau_{d-20}}{\sum_{r=1}^{20} f_r + w \sum_{d=1}^{maxlag} \tau_d} \qquad d=21, 22, 23, \ldots, 20+maxlag$$

In addition to Schneider-Wrede physicochemical distance matrix used by Chou et al, another chemical distance matrix by Grantham is also used here.

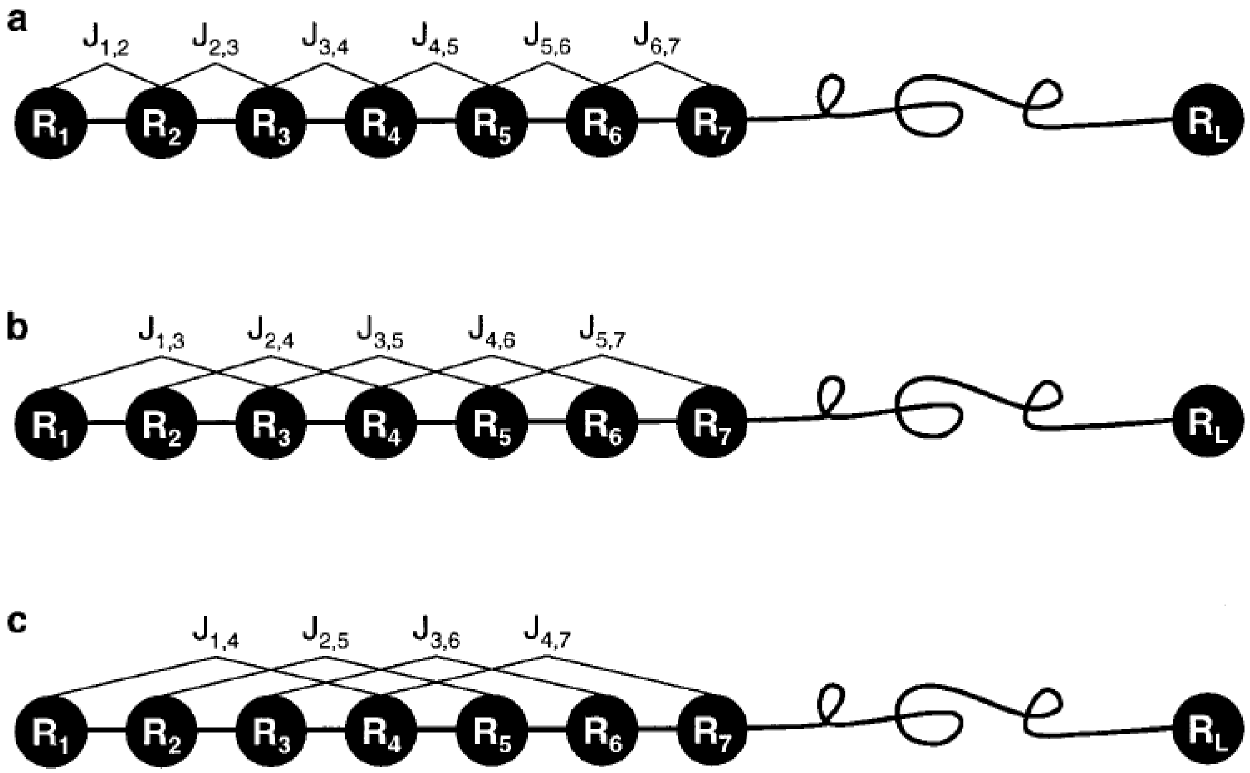

Figure 4 A schematic drawing to show (a) the 1st-rank, (b) the 2nd-rank, and (3) the 3rd-rank sequence-order-coupling mode along a protein sequence. (a) Reflects the coupling mode between all the most contiguous residues, (b) that between all the 2nd most contiguous residues, and (c) that between all the 3rd most contiguous residues. This figure is from Ref. 4.

2.8 pseudo-amino acid composition (PAAC)

This groups of descriptors are proposed by Kuo-chen Chou. PAAC descriptors (http://www.csbio.sjtu.edu.cn/bioinf/PseAAC/type1.htm) are also called the type 1 pseudo-amino acid composition. Let $H_1^o(i)$, $H_2^o(i)$, $M^o(i)$ (i=1,2,3, ..., 20) be the original hydrophobicity values, the original hydrophilicity values and the original side chain masses of the 20 natural amino acids, respectively. They are converted to following qualities by a standard conversion:

$$H_1(i) = \frac{H_1^o(i) - \frac{1}{20}\sum_{i=1}^{20} H_1^o(i)}{\sqrt{\frac{\sum_{i=1}^{20}[H_1^o(i) - \frac{1}{20}\sum_{i=1}^{20} H_1^o(i)]^2}{20}}}$$

$H_2^o(i)$ and $M^o(i)$ are normalized as $H_2(i)$ and $M(i)$ in the same way.

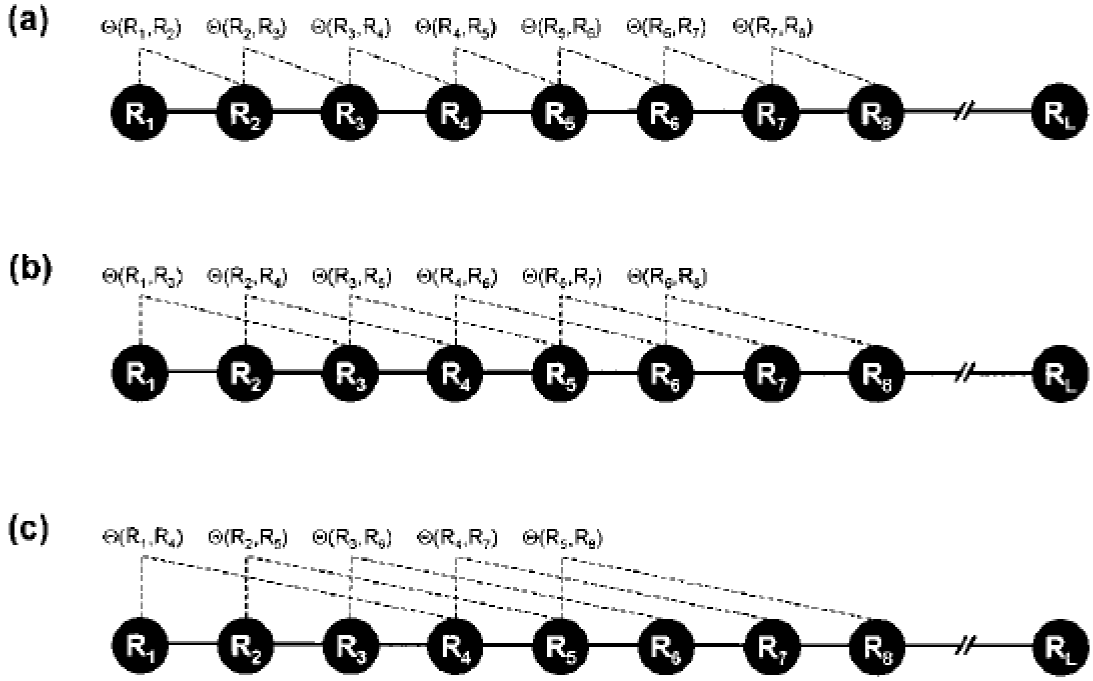

Figure 5 A schematic drawing to show (a) the first-tier, (b) the second-tier, and (3) the third-tier sequence order correlation mode along a protein sequence. Panel (**a**) reflects the correlation mode between all the most contiguous residues, panel (**b**) that between all the second-most contiguous residues, and panel (**c**) that between all the third-most contiguous residues. This figure is from Ref. 8.

Then, a correlation function can be defines as:

$$\Theta(R_i, R_j) = \frac{1}{3}\left\{\left[H_1(R_i) - H_1(R_j)\right]^2 + \left[H_2(R_i) - H_2(R_j)\right]^2 + \left[M(R_i) - M(R_j)\right]^2\right\}$$

This correlation function is actually an averaged value for the three amino acid properties: hydrophobicity value, hydrophilicity value and side chain mass. Therefore we can extend this definition of correlation function for one amino acid property or for a set of n amino acid properties. For one amino acid property, the correlation can be defined as:

$$\Theta(R_i, R_j) = \left[H_1(R_i) - H_1(R_j)\right]^2$$

where $H(R_i)$ is the amino acid property of amino acid R_i after standardization.

For a set of n amino acid properties, it can be defined as: where $H_k(R_i)$ is the kth property in the amino acid property set for amino acid R_i.

$$\Theta(R_i, R_j) = \frac{1}{n}\sum_{k=1}^{n}\left[H_k(R_i) - H_k(R_j)\right]^2$$

where Hk(Ri) is the kth property in the amino acid property set for amino acid Ri.

A set of descriptors called sequence order-correlated factors are defined as:

$$\theta_1 = \frac{1}{N-1}\sum_{i=1}^{N-1}\Theta(R_i, R_{i+1})$$

$$\theta_2 = \frac{1}{N-2}\sum_{i=1}^{N-2}\Theta(R_i, R_{i+2})$$

$$\theta_3 = \frac{1}{N-3}\sum_{i=1}^{N-3}\Theta(R_i, R_{i+3})$$

$$\cdots$$

$$\theta_\lambda = \frac{1}{N-\lambda}\sum_{i=1}^{N-\lambda}\Theta(R_i, R_{i+\lambda})$$

λ (<L) is a parameter to be chosen. Let f_i is the normalized occurrence frequency of the 20 amino acids in the protein sequence, a set of 20+λ descriptors called the pseudo-amino acid composition for a protein sequence can be defines as:

$$Xc = \frac{f_c}{\sum_{r=1}^{20} f_r + w\sum_{j=1}^{\lambda}\theta_j} \qquad (1<c<20)$$

$$Xc = \frac{w\theta_{c-20}}{\sum_{r=1}^{20} f_r + w\sum_{j=1}^{\lambda}\theta_j} \qquad (21<c<20+\lambda)$$

where w is the weighting factor for the sequence-order effect and is set as w=0.05 in PyDPI as suggested by Chou KC.

Note: the original hydrophobicity values for amino acids in PyDPI are different from the values by Chou KC. In this updated version, the default values of amino acid properties are the values of Chou KC. However, in the work of Chou KC, the definition for "normalized occurrence frequency" is not given and in this work we define it as the occurrence frequency of amino acid in the sequence normalized to 100% and hence our calculated values are not the same as values by them.

2.9 Amphiphilic pseudo-amino acid composition (APAAC)

APAAC (http://www.csbio.sjtu.edu.cn/bioinf/PseAAC/type2.htm) are also called type 2 pseudo-amino

acid composition. The definitions of these qualities are similar to the above PAAC descriptors. From $H_1(i)$ and $H_2(j)$ defined in eq. 16 and eq. 17, the hydrophobicity and hydrophilicity correlation functions are defined respectively as:

$$H_{i,j}^{1} = H_1(i)H_1(j)$$

$$H_{i,j}^{2} = H_2(i)H_2(j)$$

From these qualities, sequence order factors can be defines as:

$$\tau_1 = \frac{1}{N-1}\sum_{i=1}^{N-1} H_{i,i+1}^{1}$$

$$\tau_2 = \frac{1}{N-1}\sum_{i=1}^{N-1} H_{i,i+1}^{2}$$

$$\tau_3 = \frac{1}{N-2}\sum_{i=1}^{N-2} H_{i,i+2}^{1}$$

$$\tau_4 = \frac{1}{N-2}\sum_{i=1}^{N-2} H_{i,i+2}^{2}$$

...

$$\tau_{2\lambda-1} = \frac{1}{N-\lambda}\sum_{i=1}^{N-\lambda} H_{i,i+\lambda}^{1}$$

$$\tau_{2\lambda} = \frac{1}{N-\lambda}\sum_{i=1}^{N-\lambda} H_{i,i+\lambda}^{2}$$

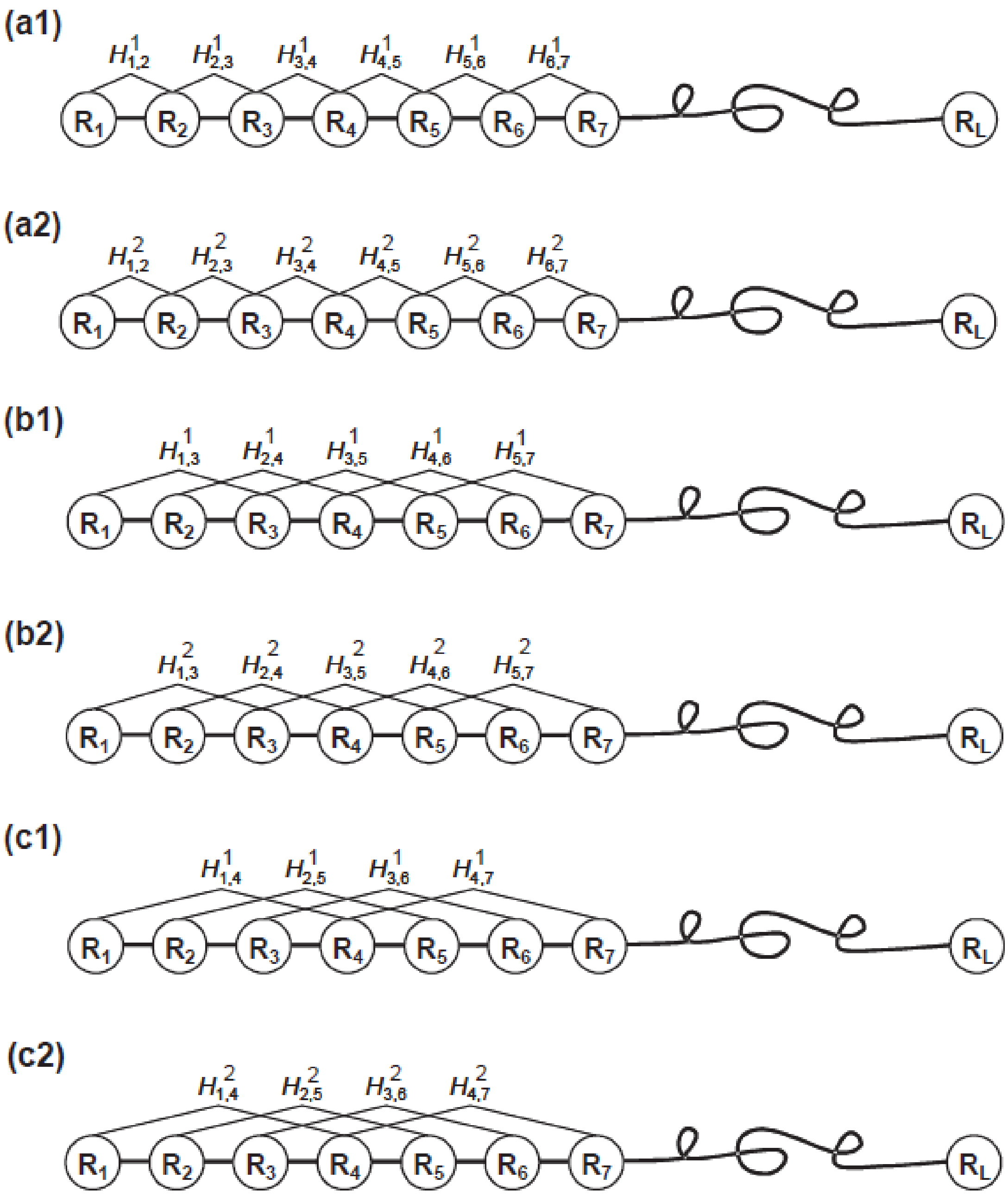

Figure 6 A schematic diagram to show (**a1**/**a2**) the first-rank, (**b1**/**b2**) the second-rank and (**c1**/**c2**) the third-rank sequence-order-coupling mode along a protein sequence through a hydrophobicity/hydrophilicity correlation function, where $H1\ i,j$ and $H2\ i,j$ are given by Equation (3). Panel (a1/a2) reflects the coupling mode between all the most contiguous residues, panel (b1/b2) that between all the second-most contiguous residues and panel (c1/c2) that between all the third-most contiguous residues. This figure is from Ref. 12.

Then a set of descriptors called "Amphiphilic pseudo amino acid composition" (APAAC) are defined

as:

$$Pc = \frac{f_c}{\sum_{r=1}^{20} f_r + w\sum_{j=1}^{2\lambda} \tau_j} \qquad 1<c<20$$

$$Pc = \frac{w\tau_u}{\sum_{r=1}^{20} f_r + w\sum_{j=1}^{2\lambda} \tau_j} \qquad 21<u<20+2\lambda$$

Where w is the weighting factor and is taken as w=0.5 in PyDPI as in the work of Chou KC.

References:

[1] M. Bhasin and G. P. S. Raghava. Classification of Nuclear Receptors Based on Amino Acid Composition and Dipeptide Composition. J. Bio. Chem. 2004, 279, 23262.

[2] Inna Dubchak, Ilya Muchink, Stephen R. Holbrook and Sung-Hou Kim. Prediction of protein folding class using global description of amino acid sequence. Proc. Natl. Acad. Sci. USA, 1995, 92, 8700-8704.

[3] Inna Dubchak, Ilya Muchink, Christopher Mayor, Igor Dralyuk and Sung-Hou Kim. Recognition of a Protein Fold in the Context of the SCOP classification. Proteins: Structure, Function and Genetics, 1999, 35, 401-407.

[4] Kuo-Chen Chou. Prediction of Protein Subcellar Locations by Incorporating Quasi-Sequence-Order Effect. Biochemical and Biophysical Research Communications 2000, 278, 477-483.

[5] Kuo-Chen Chou and Yu-Dong Cai. Prediction of Protein sub-cellular locations by GO-FunD-PseAA predictor. Biochemical and Biophysical Research Communications, 2004, 320, 1236-1239.

[6] Gisbert Schneider and Paul wrede. The Rational Design of Amino Acid Sequences by Artificial Neural Networks and Simulated Molecular Evolution: Do Novo Design of an Idealized Leader Cleavage Site. Biophys Journal, 1994, 66, 335-344.

[7] Grantham, R. Amino acid difference formula to help explain protein evolution. Science, 1974, 185, 862-864

[8] Kuo-Chen Chou. Prediction of Protein Cellular Attributes Using Pseudo-Amino Acid Composition. PROTEINS: Structure, Function, and Genetics, 2001, 43, 246–255.

[9] Jiri Damborsky. Quantitative structure–function and structure–stability relationships of purposely

modified proteins. Protein Engineering, 1998, 11, 21-30
[10] Hopp-Woods. Prediction of protein antigenic determinants from amino acid sequences. Proc. Natl. Acad. Sci. 1981, 78, 3824-3828.
[11] http://www.csbio.sjtu.edu.cn/bioinf/PseAAC/
[12] Kuo-Chen Chou. Using amphiphilic pseudo amino acid composition to predict enzyme subfamily classes. Bioinformatics, 2005, 21, 10-19.
[13] J.W. Shen, J. Zhang, X.M. Luo, W.L. Zhu, K.Q. Yu, K.X. Chen, Y.X. Li, H.L. Jiang. Predicting protein-protein interactions based only on sequences information. Proc. Natl. Acad. Sci. 2007, 104, 4337-4341.
[14] Z.R. Li, H.H. Lin, Y. Han, L. Jiang, X. Chen, Y.Z. Chen. PROFEAT: a web server for computing structural and physicochemical features of proteins and peptides form amino acid sequence. Nucleic Acids Research. 2006, 34, 32-37.
[15] H.B. Rao, F. Zhu, G.B. Yang, Z.R. Li, Y.Z. Chen. Update of PROFEAT: a web server for computing structural and physicochemical features of proteins and peptides from amino acid sequence. Nucleic Acids Research. 2011, 39, 385-390.
[16] Kawashima, S., Ogata, H., and Kanehisa, M.; AAindex: amino acid index database. Nucleic Acids Res. 1999, 27, 368-369.
[17] Kawashima, S. and Kanehisa, M.; AAindex: amino acid index database. Nucleic Acids Res. 2000, 28, 374.
[18] Kawashima, S., Pokarowski, P., Pokarowska, M., Kolinski, A., Katayama, T., and Kanehisa, M.; AAindex: amino acid index database, progress report 2008. Nucleic Acids Res. 2008, 36, D202-D205.
[19] Chou, K.-C., and Shen, H.-B. Cell-PLoc: a package of Web servers for predicting subcellular localization of proteins in various organisms. Nat. Protocols, 2008, 3, 153-162.

3 Protein-protein interaction descriptors

Let **Fa**={**Fa**(i),i=1,2,…,n} and **Fb**={**Fb**(i),i=1,2,…,n} are the two descriptor vectors for interaction protein A and protein B, respectively, then there are 3 methods to construct the descriptor vector **F** for A and B:

(1) Two vectors **F**ab and **F**ba with dimension of 2n are constructed: **F**ab = (**F**a,**F**b) for interaction

between protein A and protein B and **F**ba=(**F**b,**F**a) for interaction between protein B and protein A.

(2) One vector **F** with dimension of 2n is constructed: **F**={**F**a(i)+**F**b(i), **F**a(i) ×**F**b(i), i=1,2, …, n}.

(3) One vector **F** with dimension of n^2 is constructed by the tensor product: **F**={**F**(k)=**F**a(i) ×**F**b(j), i=1, 2, …, n, j=1, 2 ,… , n, k=(i-1) ×n+j}.

4 Protein-ligand interaction descriptors

There are two methods for construction of descriptor vector **F** for protein-ligand interaction from the protein descriptor vector $\mathbf{F}_p$ (F_p(i), i=1,n_p) and ligand descriptor vector $\mathbf{F}_l$($\mathbf{F}_l$(i), i=1,n_l)**:**

(1) One vector V with dimension of np+nl are constructed: **F**=($\mathbf{F}_p$,$\mathbf{F}_l$) for interaction between protein P and ligand L.

(2) One vector V with dimension of $n_p \times n_l$ is constructed by the tensor product: **F**={f(k)=$\mathbf{F}_p$(i) ×$\mathbf{F}_l$(j), i=1,2, …, np, j=1,2,…,n_l, k=(i-1) ×np+j}.

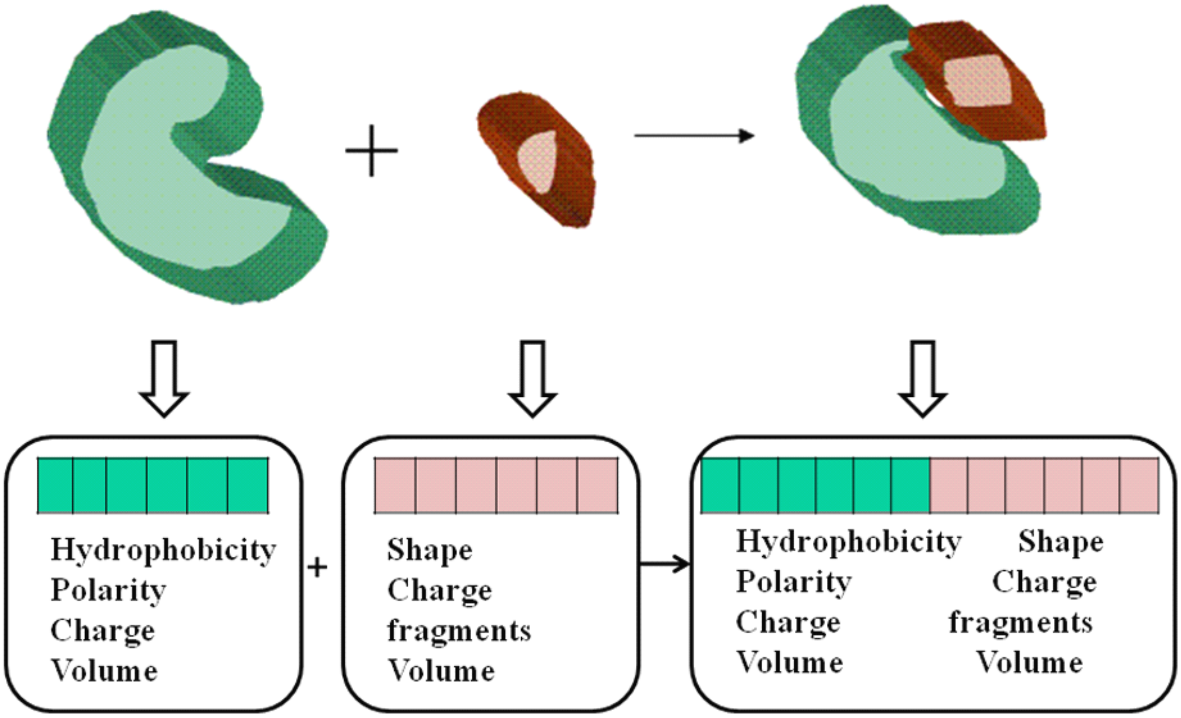

Figure 7 The schematic diagram dealing with the drug-target interaction by using the chemogenomics approach. This interaction can be considered as an event triggered by many factors influencing the binding between this drug and this protein. Therefore it can be efficiently represented by simultaneously considering the structure content from this drug and this protein under a common

chemogenomics representation framework. This figure is from Ref. 2.

References:

[1] Cao, D.-S., Liu, S., Xu, Q.-S., Lu, H.-M., Huang, J.-H., Hu, Q.-N. and Liang, Y.-Z. Large-scale prediction of drug-target interactions using protein sequences and drug topological structures. Analytica Chimica Acta, 2012, 752, 1-10.

[2] Yu, H., Chen, J., Xu, X., Li, Y., Zhao, H., Fang, Y., Li, X., Zhou, W., Wang, W. and Wang, Y. A Systematic Prediction of Multiple Drug-Target Interactions from Chemical, Genomic, and Pharmacological Data. PLoS ONE, 2012, 7, e37608.

[3] Cao, D.-S., Liang, Y.-Z., Deng, Zhe, Hu, Q.-N., He Min, Xu, Q.-S., Zhou, G.-H., Zhang, L.-X., Deng, Z.-X., Liu Shao. Genome-Scale Screening of Drug-Target Associations Relevant to Ki Using a Chemogenomics Approach. PLOS ONE, 2013, 8, e57680.

[4] Hiroaki Y., Satoshi N., Hiromu, T., Tomomi, L., Takatsugu, H., Takafumi, H., Teppei, O., Yohsuke, M., Gozoh, t., Yasushi, O. Analysis of multiple compound-protein interactions reveals novel bioactive molecules. Mol. Syst. Biol., 2011, 7, 472-483.

[5] J.W. Shen, J. Zhang, X.M. Luo, W.L. Zhu, K.Q. Yu, K.X. Chen, Y.X. Li, H.L. Jiang. Predicting protein-protein interactions based only on sequences information. Proc. Natl. Acad. Sci. 2007, 104, 4337-4341.

Appendix:

Table S1 List of PyDPI computed features for protein sequences

Feature group	Features	Number of descriptors
Amino acid composition	Amino acid composition	20
	Dipeptide composition	400
	Tripeptide composition	8000
Autocorrelation	Normalized Moreau-Broto autocorrelation	240[a]
	Moran autocorrelation	240 [a]
	Geary autocorrelation	240 [a]
CTD	Composition	21
	Transition	21
	Distribution	105
Conjoint Triad	Conjoint Triad	343
Quasi-sequence order	Sequence order coupling number	60
	Quasi-sequence order descriptors	100
Pseudo amino acid composition	Pseudo amino acid composition	50 [b]
	Amphiphilic pseudo amino acid composition	50[c]

[a] The number depends on the choice of the number of properties of amino acid and the choice of the maximum values of the *lag*. The default is use eight types of properties and *lag* = 30.

[b] The number depends on the choice of the number of the set of amino acid properties and the choice of the *lamda* value. The default is use three types of properties proposed by Chou et al and *lamda* = 30.

[c] The number depends on the choice of the *lamda* vlaue. The default is that *lamda* = 30.

Table S2 List of PyDPI computed descriptors for small molecules

Molecular descriptors		
	**Constitutional descriptors**	
1[a]	Weight	Molecular weight
2	nhyd	Count of hydrogen atoms
3	nhal	Count of halogen atoms
4[a]	nhet	Count of hetero atoms
5[a]	nhev	Count of heavy atoms
6	ncof	Count of F atoms
7	ncocl	Count of Cl atoms
8	ncobr	Count of Br atoms
9	ncoi	Count of I atoms
10	ncarb	Count of C atoms
11	nphos	Count of P atoms
12	nsulph	Count of S atoms
13	noxy	Count of O atoms
14	nnitro	Count of N atoms
15[a]	nring	Number of rings
16[a]	nrot	Number of rotatable bonds
17[a]	ndonr	Number of H-bond donors
18[a]	naccr	Number of H-bond acceptors

19	nsb	Number of single bonds
20	ndb	Number of double bonds
21	ntb	Number of triple bonds
22	naro	Number of aromatic bonds
23	nta	Number of all atoms
24	AWeight	Average molecular weight
25-30	PC1<br>PC2<br>PC3<br>PC4<br>PC5<br>PC6	Molecular path counts of length 1-6
	**Topological descriptors**	
1	W	Weiner index
2	AW	Average Wiener index
3[a]	J	Balaban's J index
4	T_{hara}	Harary number
5	T_{sch}	Schiultz index
6	Tigdi	Graph distance index
7	Platt	Platt number
8	Xu	Xu index
9	Pol	Polarity number
10	Dz	Pogliani index

11[a]	Ipc	Ipc index
12[a]	BertzCT	BertzCT
13	GMTI	Gutman molecular topological index based on simple vertex degree
14-15	ZM1<br>ZM2	Zagreb index with order 1-2
16-17	MZM1<br>MZM2	Modified Zagreb index with order 1-2
18	Qindex	Quadratic index
19	diametert	Largest value in the distance matrix
20	radiust	radius based on topology
21	petitjeant	Petitjean based on topology
22	Sito	the logarithm of the simple topological index by Narumi
23	Hato	harmonic topological index proposed by Narumi
24	Geto	Geometric topological index by Narumi
25	Arto	Arithmetic topological index by Narumi
	**Connectivity descriptors**	
1-11 [a]	${}^{0}\chi^{v}$<br>${}^{1}\chi^{v}$<br>${}^{2}\chi^{v}$<br>${}^{3}\chi_{p}^{v}$<br>${}^{4}\chi_{p}^{v}$<br>${}^{5}\chi_{p}^{v}$<br>${}^{6}\chi_{p}^{v}$<br>${}^{7}\chi_{p}^{v}$	Valence molecular connectivity Chi index for path order 0-10

	$^{8}\chi_p^{v}$ $^{9}\chi_p^{v}$ $^{10}\chi_p^{v}$	
12	$^{3}\chi^{v}_{c}$	Valence molecular connectivity Chi index for three cluster
13	$^{4}\chi^{v}_{c}$	Valence molecular connectivity Chi index for four cluster
14	$^{4}\chi^{v}_{pc}$	Valence molecular connectivity Chi index for path/cluster
15-18	$^{3}\chi^{v}_{CH}$ $^{4}\chi^{v}_{CH}$ $^{5}\chi^{v}_{CH}$ $^{6}\chi^{v}_{CH}$	Valence molecular connectivity Chi index for cycles of 3-6
19-29[a]	$^{0}\chi$ $^{1}\chi$ $^{2}\chi$ $^{3}\chi_p$ $^{4}\chi_p$ $^{5}\chi_p$ $^{6}\chi_p$ $^{7}\chi_p$ $^{8}\chi_p$ $^{9}\chi_p$ $^{10}\chi_p$	Simple molecular connectivity Chi indices for path order 0-10
30	$^{3}\chi_c$	Simple molecular connectivity Chi indices for three cluster
31	$^{4}\chi_c$	Simple molecular connectivity Chi indices for four cluster
32	$^{4}\chi_{pc}$	Simple molecular connectivity Chi indices for path/cluster
33-36	$^{3}\chi_{CH}$ $^{4}\chi_{CH}$ $^{5}\chi_{CH}$	Simple molecular connectivity Chi indices for cycles of 3-6

	${}^{6}\chi_{CH}$	
37	mChi1	mean chi1 (Randic) connectivity index
38	knotp	the difference between chi3c and chi4pc
39	dchi0	the difference between chi0v and chi0
40	dchi1	the difference between chi1v and chi1
41	dchi2	the difference between chi2v and chi2
42	dchi3	the difference between chi3v and chi3
43	dchi4	the difference between chi4v and chi4
44	knotpv	the difference between chiv3c and chiv4pc
	**Kappa descriptors**	
1	${}^{1}\kappa_{\alpha}$	Kappa alpha index for 1 bonded fragment
2	${}^{2}\kappa_{\alpha}$	Kappa alpha index for 2 bonded fragment
3	${}^{3}\kappa_{\alpha}$	Kappa alpha index for 3 bonded fragment
4	phi	Kier molecular flexibility index
5[a]	${}^{1}\kappa$	Molecular shape Kappa index for 1 bonded fragment
6[a]	${}^{2}\kappa$	Molecular shape Kappa index for 2 bonded fragment
7[a]	${}^{3}\kappa$	Molecular shape Kappa index for 3 bonded fragment
	**Burden Descriptors**	
1-16	bcutm1-16	Burden descriptors based on atomic mass
17-32	bcutv1-16	Burden descriptors based on atomic vloumes
33-48	bcute1-16	Burden descriptors based on atomic electronegativity
49-64	bcutp1-16	Burden descriptors based on polarizability

	Basak information descriptors	
1	IC0	Information content with order 0 proposed by Basak
2	IC1	Information content with order 1 proposed by Basak
3	IC2	Information content with order 2 proposed by Basak
4	IC3	Information content with order 3 proposed by Basak
5	IC4	Information content with order 4 proposed by Basak
6	IC5	Information content with order 5 proposed by Basak
7	IC6	Information content with order 6 proposed by Basak
8	SIC0	Complementary information content with order 0 proposed by Basak
9	SIC1	Structural information content with order 1 proposed by Basak
10	SIC2	Structural information content with order 2 proposed by Basak
11	SIC3	Structural information content with order 3 proposed by Basak
12	SIC4	Structural information content with order 4 proposed by Basak
13	SIC5	Structural information content with order 5 proposed by Basak
14	SIC6	Structural information content with order 6 proposed by Basak
15	CIC0	Complementary information content with order 0 proposed by Basak
16	CIC1	Complementary information content with order 1 proposed by Basak
17	CIC2	Complementary information content with order 2 proposed by Basak
18	CIC3	Complementary information content with order 3 proposed by Basak
19	CIC4	Complementary information content with order 4 proposed by Basak
20	CIC5	Complementary information content with order 5 proposed by Basak

21	CIC6	Complementary information content with order 6 proposed by Basak
	**E-state descriptors**	
1	S(1)	Sum of E-State of atom type: sLi
2	S(2)	Sum of E-State of atom type: ssBe
3	S(3)	Sum of E-State of atom type: ssssBe
4	S(4)	Sum of E-State of atom type: ssBH
5	S(5)	Sum of E-State of atom type: sssB
6	S(6)	Sum of E-State of atom type: ssssB
7	S(7)	Sum of E-State of atom type: sCH3
8	S(8)	Sum of E-State of atom type: dCH2
9	S(9)	Sum of E-State of atom type: ssCH2
10	S(10)	Sum of E-State of atom type: tCH
11	S(11)	Sum of E-State of atom type: dsCH
12	S(12)	Sum of E-State of atom type: aaCH
13	S(13)	Sum of E-State of atom type: sssCH
14	S(14)	Sum of E-State of atom type: ddC
15	S(15)	Sum of E-State of atom type: tsC
16	S(16)	Sum of E-State of atom type: dssC
17	S(17)	Sum of E-State of atom type: aasC
18	S(18)	Sum of E-State of atom type: aaaC
19	S(19)	Sum of E-State of atom type: ssssC
20	S(20)	Sum of E-State of atom type: sNH3

21	S(21)	Sum of E-State of atom type: sNH2
22	S(22)	Sum of E-State of atom type: ssNH2
23	S(23)	Sum of E-State of atom type: dNH
24	S(24)	Sum of E-State of atom type: ssNH
25	S(25)	Sum of E-State of atom type: aaNH
26	S(26)	Sum of E-State of atom type: tN
27	S(27)	Sum of E-State of atom type: sssNH
28	S(28)	Sum of E-State of atom type: dsN
29	S(29)	Sum of E-State of atom type: aaN
30	S(30)	Sum of E-State of atom type: sssN
31	S(31)	Sum of E-State of atom type: ddsN
32	S(32)	Sum of E-State of atom type: aasN
33	S(33)	Sum of E-State of atom type: ssssN
34	S(34)	Sum of E-State of atom type: sOH
35	S(35)	Sum of E-State of atom type: dO
36	S(36)	Sum of E-State of atom type: ssO
37	S(37)	Sum of E-State of atom type: aaO
38	S(38)	Sum of E-State of atom type: sF
39	S(39)	Sum of E-State of atom type: sSiH3
40	S(40)	Sum of E-State of atom type: ssSiH2
41	S(41)	Sum of E-State of atom type: sssSiH
42	S(42)	Sum of E-State of atom type: ssssSi

43	S(43)	Sum of E-State of atom type: sPH2
44	S(44)	Sum of E-State of atom type: ssPH
45	S(45)	Sum of E-State of atom type: sssP
46	S(46)	Sum of E-State of atom type: dsssP
47	S(47)	Sum of E-State of atom type: sssssP
48	S(48)	Sum of E-State of atom type: sSH
49	S(49)	Sum of E-State of atom type: dS
50	S(50)	Sum of E-State of atom type: ssS
51	S(51)	Sum of E-State of atom type: aaS
52	S(52)	Sum of E-State of atom type: dssS
53	S(53)	Sum of E-State of atom type: ddssS
54	S(54)	Sum of E-State of atom type: sCl
55	S(55)	Sum of E-State of atom type: sGeH3
56	S(56)	Sum of E-State of atom type: ssGeH2
57	S(57)	Sum of E-State of atom type: sssGeH
58	S(58)	Sum of E-State of atom type: ssssGe
59	S(59)	Sum of E-State of atom type: sAsH2
60	S(60)	Sum of E-State of atom type: ssAsH
61	S(61)	Sum of E-State of atom type: sssAs
62	S(62)	Sum of E-State of atom type: sssdAs
63	S(63)	Sum of E-State of atom type: sssssAs
64	S(64)	Sum of E-State of atom type: sSeH

65	S(65)	Sum of E-State of atom type: dSe
66	S(66)	Sum of E-State of atom type: ssSe
67	S(67)	Sum of E-State of atom type: aaSe
68	S(68)	Sum of E-State of atom type: dssSe
69	S(69)	Sum of E-State of atom type: ddssSe
70	S(70)	Sum of E-State of atom type: sBr
71	S(71)	Sum of E-State of atom type: sSnH3
72	S(72)	Sum of E-State of atom type: ssSnH2
73	S(73)	Sum of E-State of atom type: sssSnH
74	S(74)	Sum of E-State of atom type: ssssSn
75	S(75)	Sum of E-State of atom type: sI
76	S(76)	Sum of E-State of atom type: sPbH3
77	S(77)	Sum of E-State of atom type: ssPbH2
78	S(78)	Sum of E-State of atom type: sssPbH
79	S(79)	Sum of E-State of atom type: ssssPb
80-158	Smax1-Smax79	maxmum of E-State value of specified atom type
159-237	Smin1-Smin79	minimum of E-State value of specified atom type
	**Autocorrelation descriptors**	
1-8	ATSm1-ATSm8	Moreau-Broto autocorrelation descriptors based on atom mass
9-16	ATSv1-ATSv8	Moreau-Broto autocorrelation descriptors based on atomic van der Waals volume
17-24	ATSe1-ATSe8	Moreau-Broto autocorrelation descriptors based on atomic Sanderson electronegativity

25-32	ATSp1-ATSp8	Moreau-Broto autocorrelation descriptors based on atomic polarizability
33-40	MATSm1-MATSm8	Moran autocorrelation descriptors based on atom mass
41-48	MATSv1-MATSv8	Moran autocorrelation descriptors based on atomic van der Waals volume
49-56	MATSe1-MATSe8	Moran autocorrelation descriptors based on atomic Sanderson electronegativity
57-64	MATSp1-MATSp8	Moran autocorrelation descriptors based on atomic polarizability
65-72	GATSm1-GATSm8	Geary autocorrelation descriptors based on atom mass
73-80	GATSv1-GATSv8	Geary autocorrelation descriptors based on atomic van der Waals volume
81-88	GATSe1-GATSe8	Geary autocorrelation descriptors based on atomic Sanderson electronegativity
89-96	GATSp1-GATSp8	Geary autocorrelation descriptors based on atomic polarizability
	**Charge descriptors**	
1-4	Q_{Hmax}<br>Q_{Cmax}<br>Q_{Nmax}<br>Q_{Omax}	Most positive charge on H,C,N,O atoms
5-8	Q_{Hmin}<br>Q_{Cmin}<br>Q_{Nmin}<br>Q_{Omin}	Most negative charge on H,C,N,O atoms
9-10	Q_{max}<br>Q_{min}	Most positive and negative charge in a molecule
11-15	Q_{HSS}<br>Q_{CSS}	Sum of squares of charges on H,C,N,O and all toms

	Q_{NSS}<br>Q_{OSS}<br>Qass	
16-17	Mpc<br>Tpc	Mean and total of positive charges
18-19	Mnc<br>Tnc	Mean and total of negative charges
20-21	Mac<br>Tac	Mean and total of absolute charges
22	Rpc	Relative positive charge
23	Rnc	Relative negative charge
24	SPP	Submolecular polarity parameter
25	LDI	Local dipole index
	**Molecular property descriptors**	
1[a]	MREF	Molar refractivity
2[a]	logP	LogP value based on the Crippen method
3	logP2	Square of LogP value based on the Crippen method
4[a]	TPSA	Topological polarity surface area
5	UI	Unsaturation index
6	Hy	Hydrophilic index
	**MOE-type descriptors**	
1[a]	MTPSA	topological polar surface area based on fragments
2[a]	LabuteASA	Labute's Approximate Surface Area

3-14[a]	SLOGPVSA	MOE-type descriptors using SLogP contributions and surface area contributions
15-24[a]	SMRVSA	MOE-type descriptors using MR contributions and surface area contributions
25-38[a]	PEOEVSA	MOE-type descriptors using partial charges and surface area contributions
39-49[a]	EstateVSA	MOE-type descriptors using Estate indices and surface area contributions
50-60[a]	VSAEstate	MOE-type descriptors using surface area contributions and Estate indices
	**Fragment/Fingerprint-based descriptors**	
1[a]	FP2	(Topological fingerprint) A Daylight-like fingerprint based on hashing molecular subgraphs
2[a]	MACCS	(MACCS keys)Using the 166 public keys implemented as SMARTS
3	E-state	79 E-state fingerprints or fragments
4	FP4	307 FP4 fingerprints
5[a]	Atom Paris	Atom Paris fingerprints
6[a]	Torsions	Topological torsion fingerprints
7[a]	Morgan/Circular	Fingerprints based on the Morgan algorithm

Note: [a] indicates that these descriptors are from RDkit. In PyDPI, we wrapped most of molecular descriptors form RDkit. The other descriptors are independently coded by us.
